# Priming Immunization with DNA Augments Immunogenicity of Recombinant Adenoviral Vectors for Both HIV-1 Specific Antibody and T-Cell Responses

**DOI:** 10.1371/journal.pone.0009015

**Published:** 2010-02-02

**Authors:** Richard A. Koup, Mario Roederer, Laurie Lamoreaux, Jennifer Fischer, Laura Novik, Martha C. Nason, Brenda D. Larkin, Mary E. Enama, Julie E. Ledgerwood, Robert T. Bailer, John R. Mascola, Gary J. Nabel, Barney S. Graham, the VRC 009 and VRC 010 Study Teams

**Affiliations:** 1 Vaccine Research Center, Division of Clinical Research, National Institute of Allergy and Infectious Diseases, National Institutes of Health, Bethesda, Maryland, United States of America; 2 Biostatistics Research Branch, Division of Clinical Research, National Institute of Allergy and Infectious Diseases, National Institutes of Health, Bethesda, Maryland, United States of America; University of Sao Paulo, Brazil

## Abstract

**Background:**

Induction of HIV-1-specific T-cell responses relevant to diverse subtypes is a major goal of HIV vaccine development. Prime-boost regimens using heterologous gene-based vaccine vectors have induced potent, polyfunctional T cell responses in preclinical studies.

**Methods:**

The first opportunity to evaluate the immunogenicity of DNA priming followed by recombinant adenovirus serotype 5 (rAd5) boosting was as open-label rollover trials in subjects who had been enrolled in prior studies of HIV-1 specific DNA vaccines. All subjects underwent apheresis before and after rAd5 boosting to characterize in depth the T cell and antibody response induced by the heterologous DNA/rAd5 prime-boost combination.

**Results:**

rAd5 boosting was well-tolerated with no serious adverse events. Compared to DNA or rAd5 vaccine alone, sequential DNA/rAd5 administration induced 7-fold higher magnitude Env-biased HIV-1-specific CD8^+^ T-cell responses and 100-fold greater antibody titers measured by ELISA. There was no significant neutralizing antibody activity against primary isolates. Vaccine-elicited CD4^+^ and CD8^+^ T-cells expressed multiple functions and were predominantly long-term (CD127^+^) central or effector memory T cells and that persisted in blood for >6 months. Epitopes mapped in Gag and Env demonstrated partial cross-clade recognition.

**Conclusion:**

Heterologous prime-boost using vector-based gene delivery of vaccine antigens is a potent immunization strategy for inducing both antibody and T-cell responses.

**Trial Registration:**

ClinicalTrails.gov NCT00102089, NCT00108654

## Introduction

Most viral vaccines provide protection at least partially through the induction of neutralizing antibodies [1], [2]. For HIV, such antibodies have proven difficult to elicit [3], [4], and prior efficacy trials of products that did not stimulate neutralizing antibodies failed to show protection [5], [6], [7], [8]. Therefore, vaccine induction of potent, long-lived CD8^+^ T cells has become a major goal of current HIV-1 vaccine efforts [9]. This concept is supported by data showing that CD8^+^ T cell responses are associated temporally with reduction of viral load after acute infection [10], [11], specific MHC class I alleles are associated with slower progression of HIV/AIDS [12], [13], CD8^+^ T cells are largely responsible for controlling SIV viremia [14], [15], and mutation of dominant CD8^+^ T cell epitopes is a major mechanism of immune escape in HIV and SIV infection [16], [17].

Some vaccine platforms induce high frequencies of HIV-specific CD4^+^ and CD8^+^ T cells [18], [19], [20], [21]. SIV-specific T cell responses induced by such platforms do not protect monkeys against high dose SIV challenge, but do protect against high plasma viral burdens and loss of peripheral, and more importantly, gut-associated CD4^+^ memory T cells, leading to prolonged survival [22], [23]. While this protection has most often been demonstrated in monkeys challenged with homologous virus (a SIV strain that matches the vaccine insert), an HIV vaccine will need to protect against the wide diversity of circulating clades of HIV. It will therefore be important to demonstrate the breadth of the T cell response generated by a vaccine, not only in terms of the number of epitopes targeted, but also the ability of epitope-specific responses to accommodate clade-specific viral diversity.

T cells differ in their phenotype and function, and evidence suggests that these differences can impact protection against pathogens that are controlled by T cells. Non-progressive HIV infection is associated with CD8^+^ T cells that elaborate more simultaneous functions (termed polyfunctional) than is seen in progressive infection [24], and the surface phenotype of T cells may be linked to certain functions that may be important for protection. For example, expression of CD57 on CMV-specific CD4^+^ T cells is associated with MIP-1β production and direct cytolytic activity of these cells [25]. Therefore, it is important to consider both the phenotype and function of vaccine-induced T cells when evaluating their protective potential.

Here we describe the induction of HIV-1-specific antibody and T cell responses in subjects primed by DNA immunization with plasmids expressing envelope (*env*) genes from clades A, B, and C, and *gag*, *pol*, and *nef* genes from clade B [19], [20], and boosted with recombinant adenovirus serotype 5 (rAd5) vectors expressing matching genes but lacking *nef* [18]. We specifically address the phenotype, function, longevity, epitope breadth, and functional avidity of the vaccine-elicited immune response in order to better characterize the protective potential of a DNA prime, rAd5 boost vaccine regimen.

## Methods

### Ethics Statement

These studies were approved by the National Institute of Allergy and Infectious Diseases Institutional Review Board, and were performed in accordance with 45 CFR Part 46, U.S. Food and Drug Administration regulations, and principles expressed in the Declaration of Helsinki. All subjects signed written informed consent documents.

### Objectives

To characterize the magnitude, phenotype, function, breadth, and durability of the T cell response induced by DNA priming and rAd5 boosting compared to either vaccine modality given alone. A secondary objective was to characterize the antibody responses elicited by the DNA prime-rAd5 boost regimen. The protocols for this trial and supporting CONSORT checklist are available as supporting information; see Checklist S1, Diagram S1, Protocol S1 (VRC 009), and Protocol S2 (VRC 010).

### Participants

Prior recipients of candidate HIV DNA vaccines from VRC 004 (evaluation of a 4-plasmid DNA product) [20] and VRC 007 (evaluation of a 6-plasmid DNA product) [19] who consented to do so were assessed for eligibility to participate in a study evaluating a booster immunization with rAd5 [18]. Ultimately 10 subjects from VRC 004 enrolled in VRC 009 and 4 subjects from VRC 007 enrolled in VRC 010 (Table 1).

**Table 1:** Eligibility and enrollment process.

	N	
**Consented to be assessed for eligibility**		**20**
from VRC 004 (4-plasmid DNA recipients) [20]	11	
from VRC 007 (6-plasmid DNA recipients) [19]	9	
**Excluded**		**6**
not interested	1	
not eligible	5	
**Enrolled**		**14**
VRC 009	10	
VRC 010	4	

### Study Design

Studies VRC 009 (NIH 05-I-0081) and VRC 010 (NIH 05-I-0140) were Phase I, open-label, “rollover” studies of a booster injection of the VRC multiclade recombinant adenoviral vector serotype 5 (rAd5) vaccine, VRC-HIVADV014-00-VP, administered to HIV-uninfected, healthy subjects who had previously received three injections of the multiclade DNA vaccine, VRC-HIVDNA009-00-VP at either the 4 mg or 8 mg dosage in study VRC 004 (03-I-0022) [20] or of the multiclade DNA vaccine, VRC-HIVDNA016-00-VP at a 4 mg dosage in study VRC 007 (04-I-0254) [19], respectively. The interval between prior DNA immunization and rAd5 boosting varied greatly and was not used as a criterion to exclude anyone from enrollment in the roll-over studies. Subjects from both 4 mg and 8 mg dose cohorts in VRC 004 were included because earlier work had shown no significant differences in the frequency, magnitude or quality of response to primary DNA immunization between dose levels [20]. Subjects were administered a single 10^10^ PU rAd5 vector vaccination as a 1 mL intramuscular (deltoid) injection on the day of enrollment (Day 0). Studies were conducted at the National Institutes of Health (NIH) Clinical Center, Bethesda, MD by the Vaccine Research Center (VRC), NIAID, NIH, DHHS. Subjects from VRC 006 [18], which evaluated rAd5 only immunization, were used as a parallel cohort for comparison to subjects in the VRC 009 and 010 protocols. The 10 subjects in VRC 006 who received a single 10^10^ PU IM injection of the rAd5 vaccine were enrolled between 8/23/04 and 9/15/04. The same clinical staff enrolled subjects from all studies, and the same laboratory staff processed and analyzed samples with identical methodology. VRC 009 and VRC 010 were open-label and unblinded, while VRC 006 included placebo recipients and was double-blinded.

### Vaccine

The VRC-HIVADV014-00-VP vaccine is a 3∶1∶1∶1 ratio of recombinant replication-defective adenoviral vectors that encode for clade B HIV-1 Gag-Pol polyprotein and Env glycoproteins from clades A, B, and C, respectively. The vector is designed to be E1, E3, and E4 deleted, and express the recombinant gene from the E1 position in the adenovirus genome, and is produced in 293-ORF6 cells [26]. Study supplies were manufactured under current Good Manufacturing Practices (cGMP) and tested in compliance with current FDA guidance for safety, purity, potency, identity and quality before release [18].

### Peptides

Peptides (15-mers overlapping by 11) matching the sequences of the HIV-specific antigens expressed by the vaccines were used at >70% purity. They were pooled according to antigen (EnvA, EnvB, EnvC, Gag, Pol, Nef), or were pooled in a separate matrix format (see epitope mapping methods), and were used at a final concentration of 2.5 µg/ml to stimulated vaccine-induced T cells *in vitro*.

### Enzyme-Linked Immunospot Assays (ELISpot)

The frequency of antigen/vaccine-specific cells was determined as previously described [20]. Cryopreserved PBMCs were stimulated overnight by peptide pools representing the individual vaccine antigens. IFN-γ ELISpot was performed using a commercial kit (BD Biosciences), read on a CTL ELISpot image analyzer (Cellular Technology Ltd; Cleveland, OH), and expressed as mean spot-forming cells (SFC) per million PBMC.

### Flow Cytometric Analysis

Cryopreserved PBMCs were stimulated by peptide pools for 6 hours with brefeldin A. For routine analyses permeabilized fixed cells were evaluated by flow cytometry for expression of CD3, CD8, CD4, and IFN-γ and/or IL-2, then analyzed using FlowJo software (TreeStar; Ashland, OR) as previously described [20]. The following antibodies were used in various combinations to evaluate multiple functions and phenotypes of vaccine-induced T cells: CD3-Cy7APC, IFN-γ-FITC, IL-2-APC, TNFα-Cy7PE, MIP-1β-PE (BD Pharmingen; San Diego, CA); CD4-Cy5.5PE (Caltag; Burlingame, CA); CD127-PE, CD27-CY5PE, CD45RO-TRPE (Beckman Coulter; Miami, FL); PD-1-Biotin (R&D systems, Minneapolis, MN); V-amine dye (Invitrogen; Eugene, OR); CD14-PACBL, CD19-PACBL, CD8-QD705, CCR7-Alexa680, Streptavidin-QD655, and CD57-QD545 (conjugated according to standard protocols http://drmr.com/abcon/index.html). Unconjugated antibodies were obtained from BD Pharmingen and R&D Systems. Q-Dots, Alexa 680, and Pacific blue were obtained from Invitrogen; Cy5, Cy5.5, and Cy7 were obtained from Amersham Biosciences (Pittsburgh, PA) and PE and APC from ProZyme (San Leandro, CA). Cells were collected on an LSR II instrument (BD Immunocytometry Systems) configured to detect 18 fluorochromes, 350,000–1,000,000 events were collected per sample, and analysis was performed using FlowJo software version 8.4 (TreeStar). After gating, Boolean combinations of single functional gates were then created using FlowJo software to determine the frequency of each response based on all possible combinations of cytokine expression or all possible combinations of differentiation marker expression. Background responses detected in negative control tubes were subtracted from those detected in stimulated samples for every specific functional combination. The MFI of each functional parameter was also determined for all Boolean gate combinations using FlowJo software. Frequency and MFI data were processed using Pestle, version 1.5.4, Mario Roederer, Vaccine Research Center, NIAID, NIH.

### Epitope Mapping

The software program “Deconvolute This” was used to create sets of pooled peptides, such that each peptide from the overlapping 15-mers (derived from the three *envs*, *gag*, *pol*, *and nef* antigens contained in the vaccine) is present in five different pools. This 5-fold coverage is optimal for deconvolution of individual responses from this set of 869 individual peptides [27]. Peripheral blood samples were subjected to ELISpot assays using this peptide matrix to determine the number of 15-mer peptides targeted after the rAd5 boost. Targeting of two overlapping peptides was arbitrarily considered to represent a single epitope. Where cell numbers permitted, shorter peptides were used to map the minimal epitope, and ICS was used to determine the phenotype (CD4 vs CD8) of the response.

### Functional Avidity

Five serial ten-fold dilutions (5.0 to 0.0005 µg/ml) of optimally-defined peptide epitopes were used to stimulate responses in the IFN-γ ELISpot assay. Log-linear regression curves were used to determine the peptide concentration which gave half-maximal number of SFCs, and this concentration was taken as the functional avidity of the response.

### Measurement of Antibody Responses

Standardized research ELISAs were performed to delineate the antibody response to viral antigens encoded within the vaccine. End-point titers of antibodies were determined using 96-well Immulon2 (Dynex Technologies) plates coated with a preparation of purified recombinant HIV proteins derived from the same sequences as the vaccine antigens [20]. End-point titer was calculated as the most dilute serum concentration that gave an optical density reading of >0.2 above background. Subjects were screened via a commercial EIA (Abbott Laboratories HIV-1/HIV-2 rDNA) and Western blot (Mayo Laboratory, Genetic Systems Western blot kit by BioRad Laboratories, Inc).

Serum neutralizing antibody levels were measured using single round replication-defective Env-pseudoviruses and an engineered cell line that expresses luciferase upon viral infection. The methods and virus strains were previously described [28], [29].

### Data Analysis and Statistics

The statistical methods followed the same conventions that were used for the predecessor studies and were done post hoc. Measures of positive T-cell response are defined by both a statistical test and a minimum magnitude threshold. Specifically, for ELISpot, a positive response is defined as at least 50 SFC per million PBMC and a p-value of <.05 on a permutation test using a Westfall-Young correction for multiple comparisons [30]. For ICS, a positive response was defined as one with a p-value of <0.01 from a Fisher's Exact Test with a Holm adjustment for multiplicity and a background-subtracted magnitude of at least 0.0241 for CD4^+^ or 0.0445 for CD8^+^. The cutoff values for ELISpot and ICS were defined as the 99^th^ percentile of 34 HIV-negative samples stimulated with 8 different peptide pools (272 different stimulations) [18]. For ELISA, a positive response is defined as any measure with end-point titer ≥30. All paired comparisons were done using Wilcoxon Signed Rank tests (post-DNA response compared to rAd5 vector boosting). Unpaired comparisons were conducted using Wilcoxon Rank Sum tests (DNA/rAd5 recipients compared to historical controls immunized with rAd5 vector only).

The data analysis program, Simplified Presentation of Incredibly Complex Evaluations (SPICE, version 4.1.5, Mario Roederer, Vaccine Research Center, NIAID, NIH) was used to analyze and generate graphical representations of T cell responses detected by polychromatic flow cytometry. All values used for analyzing proportionate representation of responses are background-subtracted. Hence, these values can be less than zero, in cases where the background sample had more events in a particular functional gate than the positive.

## Results

### Study Design and Volunteer Demographics and Safety

Subjects from prior studies of experimental DNA vaccines, 10 of 32 (31%) available subjects from VRC 004 [20] enrolled in VRC 009 between 1/28/2005 and 7/25/05, including 5 subjects each from the 4 mg and 8 mg groups in VRC 004, and four of 14 (29%) available subjects from VRC 007 [19] enrolled in VRC 010 between 5/11/05 and 6/16//05. The demographics of the study subjects are shown in Table S1. The interval between the first dose of DNA vaccine and the rAd5 vector booster immunization ranged from 79–109 weeks (mean = 94 weeks) for the ten VRC 009 participants, and was 32–37 weeks (mean = 35 weeks) for the four VRC 010 participants. All subjects were followed for the complete 24 weeks of observation after the rAd5 vector boost. Demographic, reactogenicity, and HIV seroconversion data are recorded in Table S1, S2, and S3. There were no serious adverse events.

### T Cell Responses

**Figure 1:**
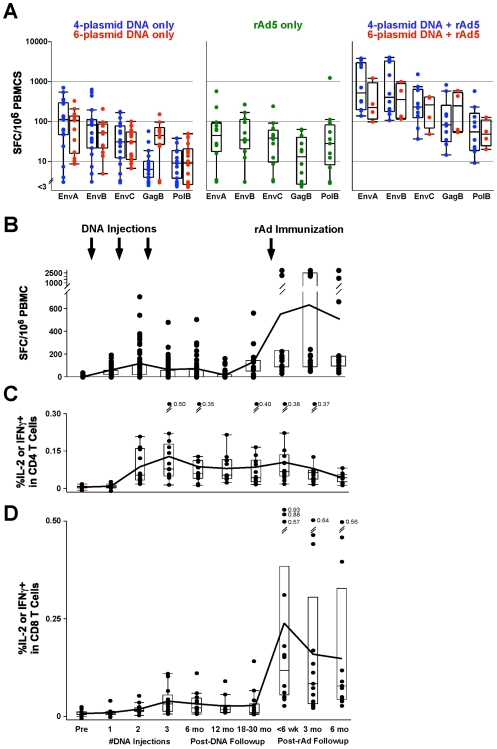
T cell responses to DNA are boosted by rAd immunization. (A) IFN-γ ELISpot responses in subjects 4 weeks after third dose of DNA or after a single dose of rAd5 vector only compared to peak response at 4–6 weeks following rAd5 vector boosting. The scale indicates spot-forming cells per million peripheral blood mononuclear cells. Individual subject responses are shown for recipients of 4-plasmid (VRC 004) or 6-plasmid (VRC 007) vaccines (left panel), the rAd5 vector (VRC 006) vaccine (center panel), and the combined DNA/rAd5 prime-boost (right panel). Results for peptide pool stimulation are shown for each peptide pool representing a single gene product in the vaccine shown on the x-axis. Boxplots represent the median and IQR. Longitudinal T cell responses to EnvA peptides were measured by IFN-γ ELISpot (B), CD4^+^ (C) and CD8^+^ (D) ICS and data are shown for all 14 subjects. The line graph shows the mean response at each time point. Some clinical time points were aggregated and are shown here as the mean value of the measurements for the grouped time points: The data following DNA injections #2 and #3 are the means of results from 2 and 4 weeks after the immunization time point; 6 month data post-DNA are the means of results from 16, 24, and 30 weeks after DNA injection #3; and the <6 week post-rAd5 time point data are means of results from 2, 4, and 6 weeks after rAd5 vector immunization.

All 14 subjects (100%) met the positivity criteria for ELISpot responses to at least one of 6 gene-specific peptide pools after rAd5 vector boost (EnvA, EnvB, EnvC, Gag, Pol, or Nef). One subject responded to 6 pools, 4 responded to 5 pools, and 5 responded to 4 pools. By combining the highest response to an Env with the responses to Gag, Pol, and Nef the total ELISpot response after rAd5 vector boost ranged from 195 to 4548 SFC/million PBMCs with a median of 891. The median magnitude of the total ELISpot response after rAd5 vector boosting was 4.5-fold higher than the total ELISpot response following DNA priming (p<.001) (Figure 1A) among the same set of volunteers, and 6-fold higher than rAd5 vector only immunized historical controls (p = .002) [18]. The total ELISpot response dropped to a median value of 370 SFC/million PBMCs (range 120–3371) by 3 months after rAd5 vector boost and then was sustained at a relatively constant level for the remainder of the 24 week observation period (data not shown). Overall the greatest response to single peptide pool was to EnvA with peak responses following the rAd5 vector boost ranging from 90 to 3682 SFC/million PBMCs. However, median Gag responses after the rAd5 vector boosting were also significantly higher than after DNA (p<.001) or rAd5 (p<.001) and ranged from 28 to 797 SFC/million PBMCs. Longitudinal plots demonstrate the increased magnitude of response post rAd5 vector boosting, followed by a reduction in magnitude to a stable response maintained beyond 6 months (Figure 1B). The peak EnvA-specific response post rAd5 vector boost is plotted as the mean of the 2, 4, and 6 week data.

CD4^+^ T cell responses measured by ICS for IFN-γ and/or IL2 were detected after stimulation with at least one peptide pool, each pool representing a single gene product, in 13/14 subjects after rAd5 vector boosting. Two subjects responded to 6 gene product pools, four responded to 5 pools, one responded to four peptide pools, and six responded to three (data not shown). Combining the responses to the distinct gene-specific peptide pools, the summed (best Env + Gag + Pol + Nef) CD4^+^ T cell ICS response after rAd5 vector boosting ranged from 0.065 to 0.69% of total CD4^+^ T cells, with a median of 0.22%. The median magnitude of the total CD4^+^ T cell ICS response 4 weeks following rAd5 vector boosting was about the same as detected in rAd5 vaccine-only immunized historical controls [18]. The magnitude of peak CD4^+^ T cell ICS response to EnvA after rAd5 vector boosting ranged from 0 to 0.45 percent of total CD4^+^ T cells and was stable and persisted at a constant level for the remainder of the 6 month follow-up period. Unlike the ELISpot and CD8^+^ T cell responses, the magnitude of CD4^+^ T cell responses was not elevated significantly after the rAd5 vector boosting (Figure 1C).

CD8^+^ T cell responses measured by ICS for IFN-γ and/or IL-2 were detected after stimulation with at least one gene-specific peptide pool in 10 subjects after rAd5 vector boosting. Two subjects responded to 5 pools, one responded to 4 pools, and four responded to 3 pools (data not shown). The summed CD8^+^ T cell ICS response after rAd5 vector boost ranged from 0.02 to 2.32% of total CD8^+^ T cells, with a median of 0.25%. The median magnitude of the total CD8^+^ T cell ICS response following rAd5 vector boosting was 7-fold higher than the total CD8^+^ T cell ICS response after DNA priming (p<.001) (Figure 1D), and 5-fold higher than rAd5 vector only immunized historical controls (p = .057) [18]. The total CD8^+^ T cell ICS response dropped to a median value of 0.15% total CD8^+^ T cells (range 0.003–2.04) by 3 months after rAd5 vector boost and then was sustained at a constant level for the remainder of the 6 month follow up period. The magnitude of peak response by CD8^+^ T cell ICS ranged from 0.01 to 1.53% of total CD8^+^ T cells to EnvA; longitudinal plots of the EnvA-specific response demonstrate that the increase in CD8^+^ T cell responses after rAd5 vector boosting correlates with the increase in ELISpot responses (Figure 1). The Gag-specific response after rAd5 boosting ranged from 0 to 0.57% of total CD8^+^ T cells and was significantly higher than after DNA alone (p = .004), and it trended higher than after rAd5 only, but did not reach statistical significance (p = .291).

The magnitude of the IFN-γ ELISpot response after rAd5 boost correlated with the magnitude of those measurements after DNA priming (p = .001 by Spearman non-parametric test). There was also a suggestion of a correlation between CD8^+^ T cell ICS responses after the rAd5 boost and after the DNA prime, but this did not reach statistical significance (p = .061) This suggests effective DNA priming was an important determinant of the response to rAd5 boosting.

### Phenotype of Responding Cells

**Figure 2:**
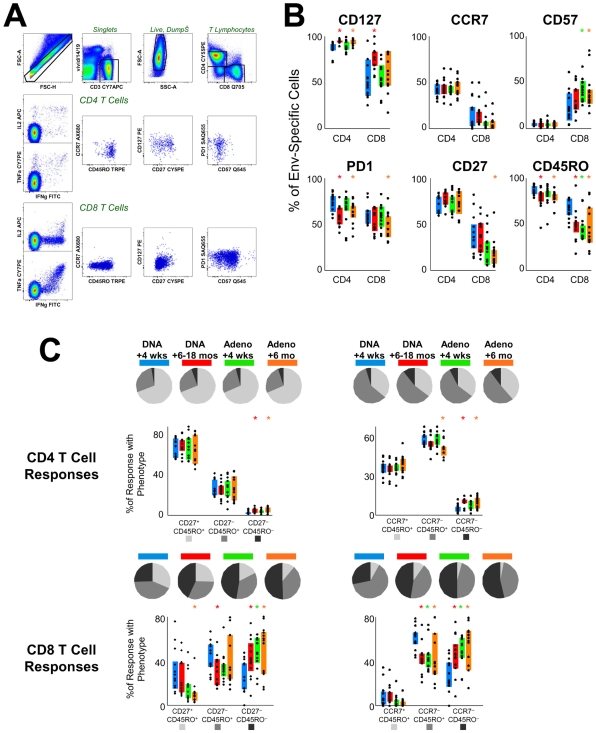
Phenotype of vaccine-induced T cells. (A) Flow cytometric analysis of antigen-specific T cells. The top row of graphs shows the progressive gating to identify live CD3^+^CD4^+^ or CD3^+^CD8^+^ lymphocytes. For either CD4 or CD8 T cells, antigen-responsive cells (following *in vitro* stimulation) are identified by the production of IL2, IFN-γ, or TNF. This example shows EnvA-stimulated cells from one of the highest responders in the study; the phenotyping graphs illustrate the distribution of cells that make any cytokine (blue) overlaid on the total CD4 or CD8 population (grey). (B) The distribution of expression of a variety of cell surface markers on antigen-specific CD4 or CD8 T cells following vaccination. The colored bars represent the IQR for the distribution at different time points: 4 weeks post-DNA (blue); 6–18 months post DNA (red); 4 weeks post rAd5 boost (green); and 6 months post-boost (orange). (C) Two different phenotyping schemas, based on either CD27 or CCR7 expression in combination with CD45RO, were used to characterize antigen-specific T cells. The bar charts show the individual data points and IQR for the four time points following immunization. The pie charts summarize these distributions, showing the average proportion of the CD4 or CD8 vaccine-specific T cell response that is T_CM_ (light grey), T_EM_ (medium grey), or T_EF_ (black). Asterisks indicate distributions that are different from the earliest time point (4 weeks post DNA) at p<0.05 (Student's T test).

We used a 13-color immunophenotyping panel to characterize the antigen-specific T cells induced by the vaccine. Cryopreserved specimens for all individuals at four different time points were assessed (4 weeks following the last DNA immunization; 6–18 months later, immediately prior to rAd5 boosting; 4 weeks after rAd5 boosting; and 6 months following rAd5 boosting). These represent the “effector” and “memory” time points following the priming and boosting stages. Included in the panel were reagents to identify T cell lineages (CD3^+^CD4^+^ or CD3^+^CD8^+^), responding cells (IFN-γ^+^ or IL-2^+^ or TNF^+^), as well as memory/differentiation stages (CD127, CCR7, CD57, PD-1, CD27, and CD45RO). Figure 2A illustrates the “gating tree” used to identify the responding cells, and a representative example of the phenotypes of these responding cells compared to the entire CD4^+^ or CD8^+^ T cell population. Responding cells had a phenotype associated with memory cells.

The expression of the six differentiation markers on vaccine-induced T cells is shown in Figure 2B, as a function of time. CD127 expression was uniform on Env-specific CD4^+^ T cells, and on most Env-specific CD8^+^ T cells, suggesting that the vaccine-induced T cells are long-lived memory cells capable of homeostatic expansion [31]. CD4^+^ T cells tended to express more markers associated with being “central memory” (CCR7 and CD27) [32], [33]; CD8^+^ T cells appear to be more differentiated. Indeed, within the CD8^+^ population, 20–30% expressed CD57, a phenotype consistent with terminal effector cells [34]. Within either lineage, about half of the cells expressed PD-1. Consistent with the fact that antigen stimulation induces PD-1 expression on activated T cells, the expression of this marker declined mildly over time after DNA or rAd vaccination. In contrast, there was little change in the expression of the other phenotypic markers on the antigen-specific cells over time.

We further characterized the differentiation stages of the responding T cells as falling into central memory (T_CM_), effector memory (T_EM_), or terminal effector (T_EF_) stages. The original definition of these differentiation stages is derived from the measurement of CCR7 and CD45RO [33]; however, others have used CD27 and CD45RO to assign these stages [32], [35]. In Figure 2C, both phenotypic characterizations are shown for the vaccine-induced T cells. Within the CD4^+^ subset, the cells are strongly biased towards T_CM_ cells; the representation within the different subsets does not change at all over time. Within CD8^+^ T cells, using either phenotyping model, there was a significant increase of T_EF_ and a concomitant decrease of T_CM_ (or T_EM_) over time.

### Quality of Responding Cells

**Figure 3:**
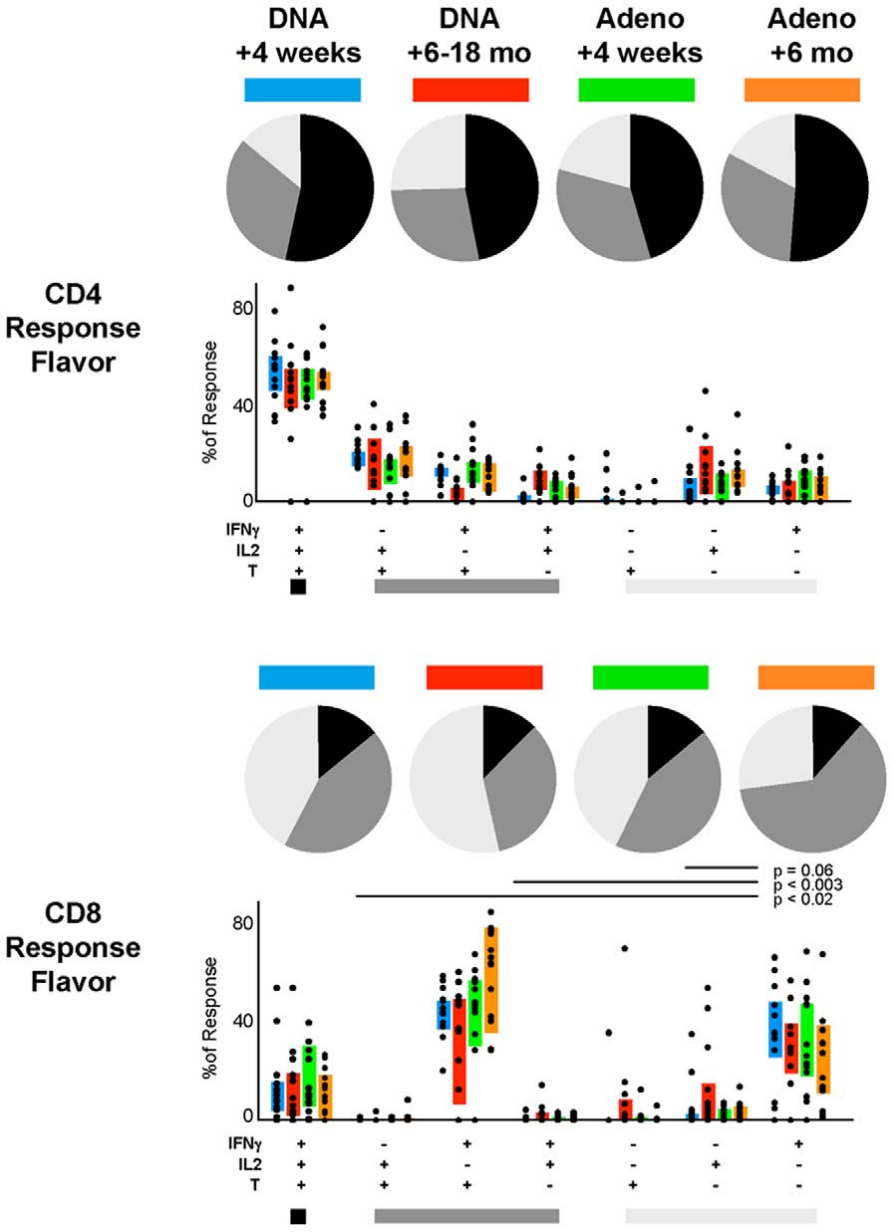
Function of vaccine-induced T cells. The quality of the vaccine-elicited CD4 or CD8 T cell response is characterized by the proportion of cells making every possible combination of the three measured cytokines. The bar charts show the individual data points and IQR for four time points following immunization. Pie charts show the average proportion of the CD4 or CD8 vaccine-specific T cell response that is polyfunctional (black), producing two functions (medium grey), or is monofunctional (light grey). The memory time point had a significantly different quality of CD8 T cell response than earlier time points (SPICE permutation analysis).

The quality of a T cell response can be characterized in part by the patterns of cytokine production. We have previously shown that T cells can be divided into those that make only one cytokine (monofunctional), two cytokines, or multiple cytokines simultaneously (polyfunctional). The vaccine regimen induced polyfunctional T cells in both the CD4^+^ and CD8^+^ lineages (Figure 3). The CD4^+^ T cells were highly polyfunctional, in that a majority produced all three cytokines, and less than 25% were monofunctional. CD8^+^ T cells were predominantly IFN-γ^+^ and either TNF^+^ or TNF^−^. There was a trend towards increasing functionality in the CD8^+^ T cells after rAd boosting, as evidenced by the decreasing proportion of monofunctional T cells.

Recent reports show that the amount of cytokine produced by individual antigen-specific T cells can vary dramatically depending on how they were elicited [36], [37]; those cells with the highest per-cell production are optimized for effector activity and protection. The cytokine production can be estimated by the median fluorescence intensity (MFI) of the cytokine staining for the responding cells. For the vaccine-induced T cells, we observe a consistent and dramatic increase in the IFN-γ and TNF MFIs for polyfunctional cells compared to less functional cells (Figure S1). In addition, we observe that the IFN-γ MFI for CD4^+^ polyfunctional cells increases substantially after rAd5 boosting (Figure S1B). This suggests that the polyfunctional T cells induced by the DNA/rAd5 prime/boost exhibit better effector function than cells primed by DNA alone.

### Quality and Phenotype

**Figure 4:**
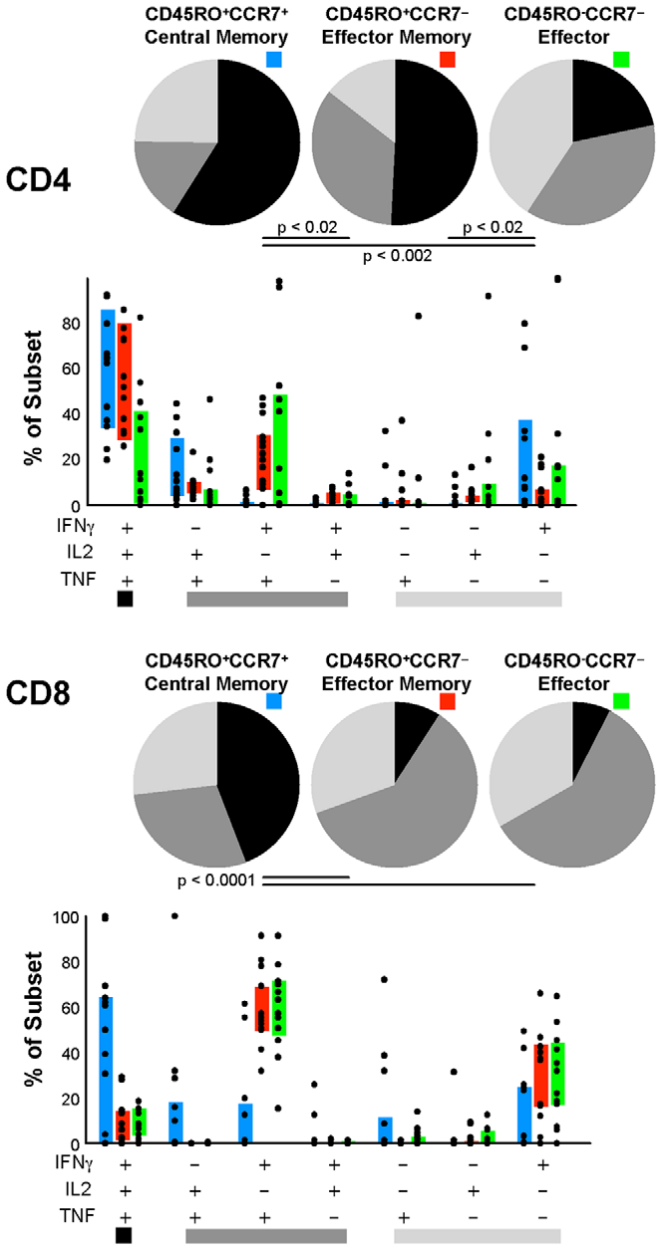
The quality of the response within T_CM_, T_EM_, or T_EF_ cells. Data for each subject is averaged over time points (there was no statistically significant change in quality over time within each subset). T_CM_ cells have significantly more polyfunctional T cells than T_EM_ or T_EF_ cells.

Since our immunophenotyping panel included both the functional measurements as well as the memory/differentiation measurements, we could begin to assess the relationship of function to differentiation stage. The complexity of this analysis is enormous, as the number of potential distinct phenotypes (2^6^ = 64) and functional states (2^3^ = 8) to be analyzed in combination, for both CD4^+^ and CD8^+^ T cells, well exceeds the statistical power of this relatively small sample set. We therefore restricted our current analysis to determine the quality of the response within T_CM_, T_EM_, and T_TE_ subsets (defined on the basis of CCR7 and CD45RO expression). As shown in Figure 4, the most polyfunctional subsets within both the CD4^+^ and CD8^+^ lineages belong to the T_CM_ subset. In contrast, the T_EM_ (and T_EF_) subsets are highly enriched for the IFN-γ^+^TNF^+^ or IFN-γ^+^ cells, particularly within CD8^+^ T cells.

### Breadth of the T Cell Response

Epitope mapping was carried out on nine of the volunteers from VRC 009. Between one and ten epitopes were mapped in each volunteer, with an average of 3.33 epitopes per volunteer (Table 2). Where cell numbers permitted, several further studies were done to characterize the targeted epitopes. Intracellular cytokine staining was performed to determine whether the response was mediated by CD4^+^ or CD8^+^ T cells, shorter peptides were used to map the minimum epitope, and peptide titrations were performed with the minimum epitope to determine the functional avidity of the response (peptide concentration giving half-maximal response). In many cases previously-described epitopes with known HLA restrictions were mapped in these vaccine volunteers. Among the known epitopes that were targeted were the A03-restricted responses to TVYYGVPVWK and RLRPGGKKKY in envelope (EnvTK10 and EnvRY10, respectively), the B57-restricted response to TSTLQEQIGW in Gag (GagTW10), and the A29 or A32-restricted response to RIKQIINMW in envelope (EnvRW9) [38].

**Table 2:** Epitope mapping.

Antigen	Peptide Sequence*	ELISpot (SFC/10^6^ PBMC)	Functional avidity (µg/ml)**	ICS***	HLA****
Subj A (A01, A26, B5601, B57, Cw1, Cw6)*****					
EnvA	**CAPAGFAIL**	1220	0.0135	CD8	ND
EnvB	MRVKEKYQHLWRWGW	3640		CD8	
EnvB	DAKAYDTEVHNVWAT	840			
EnvB	**RIKQIINMW**	2035	ND	CD8	ND
GagB	**TSTLQEQIGW**	425	0.548	CD8	B57
Subj C (A02, A03, B07, B15, Cw3, Cw7)					
EnvA	**AVYYGVPVWK**	2150	0.060	CD8	A03
EnvA	AMYPPPIQGVIRCES	550			
EnvB	**TVYYGVPVWK**	2550	0.0006	CD8	A03
Subj D (A02, A31, B15, B44, Cw3, Cw5)					
EnvA	AMYPPPIQGVIRCES	98			
EnvC	PGQTFYATGDIIGDI	126			
GagB	IVKCFNCGKEGHTAR	116			
Subj E (A01, A03, B07, B7301, Cw7, Cw15)					
EnvA	LWV***AVYYGVPVWK***DA	71			A03
Subj F (A03, A32, B07, B55, Cw3, Cw7)					
EnvBEnvB	KLWV***TVYYGVPVWK***E ***TVYYGCPVWK***EATTT	6835			A03
Subj G (A03, A24, B15, B35, Cw3, Cw4)					
EnvA	LWV***AVYYGVPVWK***DA	1315			A03
EnvAEnvA	CNTSAITQACPKVSFAITQACPKVSFEPIP	1670805			
EnvB	***TVYYGVPVWK***EATTT	1585			A03
Subj H (A01, A02, B52, B57, Cw6, Cw12)					
EnvA	MRVRGIQTSWQNLWR	191		CD8	
GagB	**TSTLQEQIGW**	75	ND	CD8	B57
Subj I (A02, A31, B4901, B51, Cw7, Cw14)					
EnvA	IRSENITNNAKTIIV	113		CD4	
EnvBEnvB	KLWVTVYYGVPVWKETVYYGVPVWKEATTT	133135		CD4	
Subj J (A03, A24, B07, B18, Cw7)					
EnvA	**AVYYGVPVWK**	2300	0.345	CD8	A03
EnvA	GIIGDIRQAHCHVSR	187		CD4	
EnvA	KQIINMWQKVGQAMY	67		CD4	
EnvAEnvA	LTVWGIKQLQARVLAGIKQLQARVLAVERY	9267		CD4	
EnvA	**NYTQIIYNL**	830	0.183	CD8	ND
EnvB	SATEKLWVTVYYGVP	3077		CD8	
EnvB	**TVYYGVPVWK**	2245	0.0033	CD8	A03
EnvBEnvB	CPKVSFEPIPNHYCASFEPIPNHYCAPAGF	257202			
EnvB	RIKQIINMWQKVGKA	137		CD4	
GagB	**RLRPGGKKKY**	275	0.611	CD8	A03

*Peptide sequence used to map epitope. Where a minimum epitope was determined, the sequence is shown in bold. Where a minimum epitope is presumed based upon the 15-mer peptide mapping and expression of the correct HLA, it is shown in bold italics. Otherwise, the individual 15-mer or two overlapping 15-mers to which the response was mapped are shown. Responses were mapped from a time point after the rAd boost.

**Functional avidity of response to the minimum epitope expressed as the peptide concentration giving half-maximal response by ELISpot. ND  =  not determined.

***CD4 or CD8 phenotype of the peptide response by intracellular cytokine staining. Empty boxes  =  undetermined.

****HLA restriction of known or presumed minimum epitopes. ND  =  not determined.

*****Two digit HLA typing on the volunteers.

Regions previously not known to contain CD4^+^ and CD8^+^ T cell epitopes were also identified, such as a CD8^+^ T cell epitope in envelope CAPAGFAIL (EnvCL9). It should also be noted that we often found a lower magnitude of response when we mapped down to the minimum epitope, suggesting that there may be other overlapping epitopes within some of our 15-mer responses that we have failed to further characterize. This suggests that our methods underestimate the number of epitopes targeted in these volunteers.

### Cross-Clade Recognition

**Figure 5:**
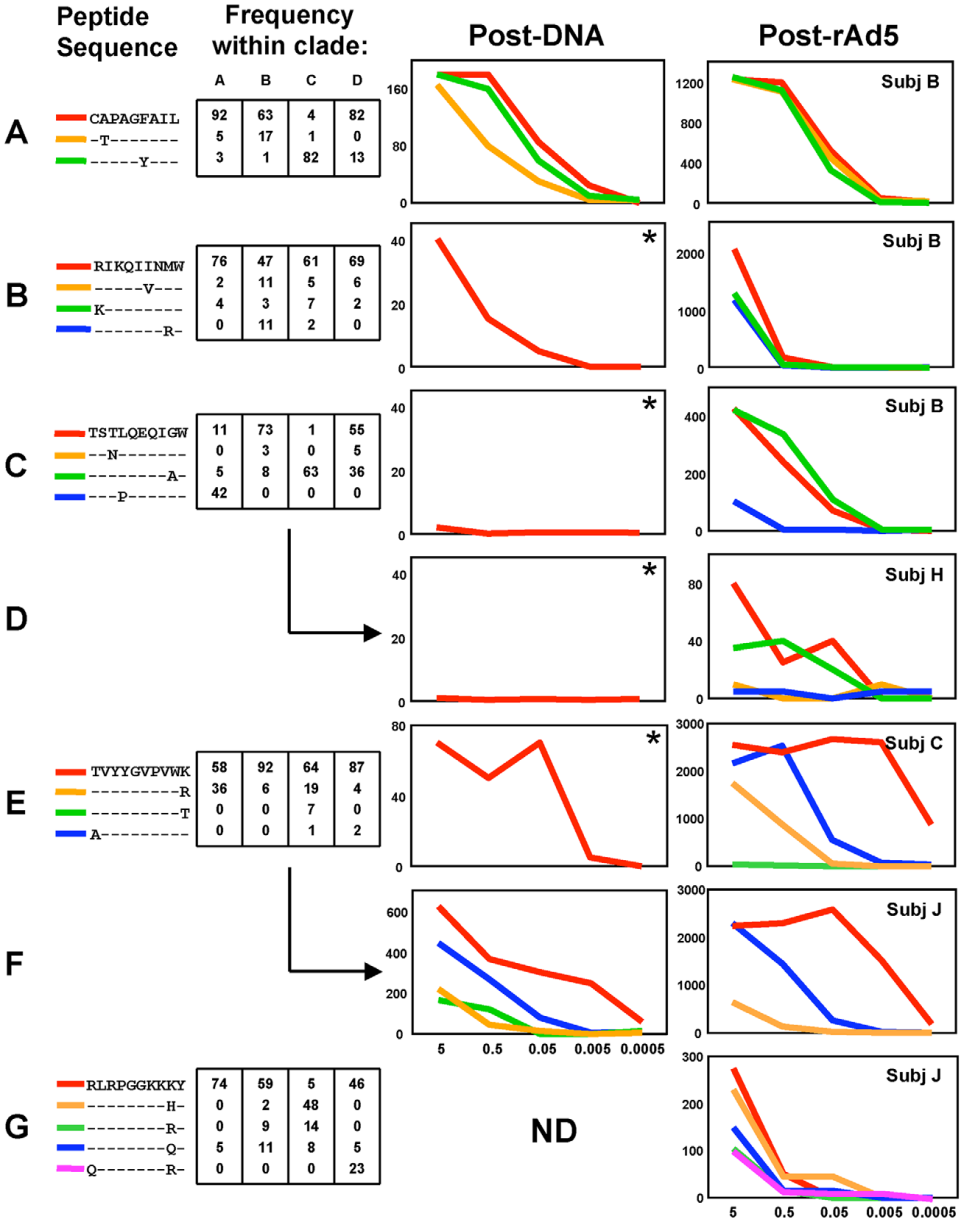
Clade coverage of epitope-specific responses. Seven responses (A through G) to five minimum CD8^+^ T cell epitopes as defined in Table 2 are shown for four VRC 009 volunteers. Volunteer identifiers are shown in far right panels. Left panel shows the minimum epitope and sequence variants that were tested. Left center panel shows the frequency of each variant as it occurs within HIV clades A, B, C, and D. ELISpot responses expressed as SFCs per million PBMC at multiple peptide dilutions (µg/ml) to the epitope variants are shown from 4 weeks after the third dose of DNA (right center panel) and 4 weeks after rAd boost (far right panel). *  =  only the major variant was tested due to cell limitations. ND  =  not done due to absence of cells for any peptide titrations.

We next investigated to what extent the epitope-specific responses elicited by the vaccine would cover the sequence variability within isolates from clades A, B, C and D. Peptides corresponding to the major clade A, B, C, and D variants were synthesized for five minimum epitopes in Gag and Env. These were used in ELISpot assays at multiple different concentrations to determine the recognition patterns for the vaccine-elicited responses to these five epitopes (Figure 5). In all except one instance, we were able to test the recognition (at least against the predominant sequence) after DNA priming, and again after rAd5 boosting.

A wide range of epitope-specific functional avidities was evident, with some epitopes (EnvCL9 and EnvRY10) inducing IFN-γ production only at the highest peptide concentration (5 µg/ml, Figure 5B and G) while others (EnvTK10) were active out to very low peptide concentrations (5 ng/ml, Figure 5E and F). Three of the four responses that were tested after DNA priming (EnvCP9, EnvRW9, and EnvTK10) were detectable at that time point (Figure 5A, B, E, and F), and in all cases the frequency of those responses increased after the rAd5 boost. While the boost augmented the frequency of the responses, it did not appear to have a major impact on either the functional avidity, or the recognition pattern of the epitope variants. The responses against the B57-restricted epitope GagTW10 were not detectable after the DNA prime in two volunteers (Figure 5C and D) but were readily detectable after the boost.

Where more than one volunteer had a detectable response against a given epitope, the functional avidities and patterns of response to the epitope variants were similar. Specifically, the functional avidities and pattern of recognition of epitope variants were similar in the two volunteers who generated a response to GagTW10 (Figure 5C and D). The same was true of the responses to EnvTK10 (Figure 5E and F). Of note is the fact that wherever we were able to map epitopes and test for recognition of clade variants, we routinely found broad recognition of most of the major variants. The notable exception was the absent response to the dominant clade A variant in GagTW10 (Figure 5C and D). However, the responses in these two volunteers were still able to strongly recognize the two sequences that covered the majority of clade B, C, and D variants. Concentrating on HLA-A and B alleles, we found that there were five HLA-A alleles and 3 HLA-B alleles that were present more than once within our nine volunteers. None of the mapped CD8^+^ T cell responses were restricted by HLA-A01, A02, A24, A31, B07, or B15. However, six responses restricted by HLA-A03 and two restricted by HLA-B57 were mapped. Every volunteer who expressed HLA-A03 (n = 5) generated a response to EnvTK10 and both volunteers who expressed HLA-B57 generated a response to GagTW10, suggesting strong recognition of these two epitopes in the antigens expressed by the vaccine.

### Antibody Responses

All subjects had detectable Env-specific antibody responses measured by ELISA (Table 3). The response was about equal to each of the EnvA, EnvB, and EnvC glycoproteins, but responses to Gag, Pol, and Nef were low or undetected. The median reciprocal titer of the 14 subjects to EnvC was 6,000, which is about 100-fold higher than the response following DNA priming (p<.001) or following the 10^10^ PU dose of rAd5 vector using historical data (p<.001) (Figure S2). Although the DNA/rAd5 generated moderately high binding antibody titers to Env, only low levels of serum neutralizing antibody activity were observed. Sera from five subjects neutralized selected viruses (SF162 and MW965.26) considered to be relatively easy to neutralize (Table 3) [39], [40]. Other primary virus isolates were not neutralized by the vaccinee sera.

**Table 3:** Antibody responses induced in subjects after priming with DNA and boosting with rAd5 vaccines.

ELISA Endpoint Titer				IC_50_ Neutralization Titer									
				Clade A			Clade B				Clade C		
EnvA	EnvB	EnvC	Gag	DJ263.8	MS208.A1	RW020.02	SF162.LS	HXB2	Bal.26	SS1196	MW965.26	DU156.12	Br025
12000	12000	12000	15	-	-	-	-	-	-	-	-	-	-
324000	2880	12000	5760	-	-	-	6	-	-	-	10	-	-
6000	2880	2880	270	-	-	-	16	-	-	-	-	-	-
1440	270	270	15	-	-	-	-	-	-	-	-	-	-
30720	30720	30720	270	-	-	-	-	-	-	-	-	-	-
36000	12000	12000	15	-	-	-	-	-	-	-	-	-	-
36000	6000	6000	3000	10	-	-	19	-	-	-	4164	-	-
96000	36000	48000	30	7	-	-	-	-	-	-	25703	-	-
12000	3750	12000	15	-	-	-	-	-	-	-	-	-	-
750	150	750	15	-	-	-	-	-	-	-	-	-	-
12000	3750	3750	15	-	-	-	-	-	-	-	-	-	-
12000	48000	48000	750	-	-	-	5	-	-	-	103	-	-
720	720	1440	15	-	-	-	-	-	-	-	-	-	-
750	750	3750	15	-	-	-	-	-	-	-	-	-	-

## Discussion

We evaluated safety and HIV-specific immune responses in healthy adult volunteers who received a priming immunization with a 4-plasmid or 6-plasmid candidate HIV-1 DNA vaccine and subsequently received a booster immunization with a matching candidate HIV-1 rAd5 vaccine. This was a non-randomized, exploratory Phase I evaluation using a roll-over design and provided the first opportunity to boost subjects with rAd5 who had been primed in prior studies with DNA vaccines. This small group of rollover study participants allowed an early evaluation of the DNA prime-rAd5 boost vaccination concept in humans. This study focused on the detailed evaluation of vaccine-induced immune responses. While interpretations should be tempered by the relatively small study size, exploratory nature of the analysis, and use of historical controls for the rAd5 only comparison, there are a number of significant observations that merit additional investigation. Larger studies are being conducted to provide a more robust statistical assessment of general vaccine immunogenicity, and to determine how the observations made in these studies relate to vaccine efficacy.

Both components of the vaccine had been well-tolerated and were immunogenic as single agents in previous Phase I clinical trials [18], [19], [20]. The combination vaccine was also well-tolerated, and the sequence of DNA priming and rAd5 vector boosting was far more immunogenic than either the DNA or rAd5 vaccine alone. HIV-specific T cell responses, detected by both ELISpot and ICS were present in 100% of the subjects and were 5-fold higher than in subjects immunized with rAd5 vector alone. The T cell responses were (i) biased toward CD8^+^ more than CD4^+^; (ii) more polyfunctional than T cell responses induced by DNA alone; (iii) sustained for >6 months; (iv) directed against multiple epitopes biased toward MHC class I restriction; and (v) specific for multiple antigens (Envelope > Gag > Pol/Nef). The relative response to Envelope antigens was also similar to that seen after DNA immunization alone (EnvA  =  EnvB > EnvC). Antibody responses were also induced in 100% of the subjects and were >100-fold higher than in subjects immunized with a comparable dose of rAd5 vector alone. The augmented antibody response did not have significant neutralizing activity to tier 2 viruses (Table 3) [40].

Two observations suggest that the improved response after rAd5 vector boosting was related to immunological priming by DNA. First, the pattern of the response after rAd5 vector boost of relatively dominant Env responses, intermediate Gag responses, and relatively lower Pol responses was similar to that observed after the 4-plasmid DNA immunization [20]. Secondly, multivariate correlates analysis showed the response measured after DNA priming was the best predictor of IFN-γ ELISpot and CD8^+^ T cell ICS magnitude after rAd5 vector boosting. In addition, preclinical data and recently reported human data using a different rAd5 vector suggest that augmented HIV-specific T cell responses are not seen in subjects immunized with a second dose of rAd5 [41].

The mechanisms underlying the benefit of prior DNA immunization are unknown. HIV-specific CD4^+^ T cell responses measured by ICS were persistent following DNA immunization, and their presence may have aided in the rapid expansion of the CD8^+^ T cell response during boosting. Interestingly, there was no apparent expansion of HIV-specific CD4^+^ T cells following rAd5 boosting (Figure 1C). Therefore, it is equally likely that the augmented CD8^+^ T cell responses after rAd5 vector boosting were simply a reflection of the expansion of DNA-induced CD8^+^ T cell responses. This conclusion is supported by the finding that CD8^+^ T cell responses were noted in subjects immediately after DNA priming, and that the Nef-specific responses were still detectable in some individuals more than 2 years later. Since *nef* was not included in the rAd5 vector, DNA immunization alone was responsible for these responses. Of note, there was no difference in the post-boost response patterns in subjects primed with 4 mg vs. 8 mg of DNA in VRC 004, which is consistent with the primary responses measured after DNA immunization [20].

The use of heterologous platforms to initially prime and subsequently boost an immune response has been shown to be effective for eliciting antibody responses [42], [43], [44], [45], [46] but is especially attractive for augmenting T cell responses [47], [48], [49], [50]. The combination of DNA priming and recombinant viral vector boosting has been shown to induce potent immune responses and protection against several pathogens, including Ebola and Malaria [51], [52], and has also been shown to reduce virus load and delay disease progression in macaques challenged with SHIV or SIV [22], [23], [53]. One reason for this success may be that combining two different modalities in a heterologous prime/boost regimen appears to induce responses that differ from those induced by repeated dosing of either component alone. Specifically, a recent report showed that T cell responses to an HIV candidate vaccine expressing Gag, Pol, and Nef in a heterologous DNA/rAd5 regimen were different from those induced by a homologous rAd5/rAd5 regimen in humans [54]. The heterologous DNA/rAd5 induced a greater Gag-specific CD4^+^ T cell response than the homologous rAd5/rAd5 regimen. In addition, both the Gag-specific CD4^+^ and CD8^+^ T cell responses induced by DNA/rAd5 had a greater frequency of IL-2 producing cells and more phenotypic diversity in the DNA/rAd5-induced T cells than in those induced by rAd5/rAd5. The homologous rAd5/rAd5 regimen also appeared to induce a greater degree of polyfunctional T cell responses than seen in the current study following a single dose of rAd5.

Another important consequence of using heterologous vector combinations is to focus the immune response on the recombinant gene product instead of the vector. Pre-existing vaccinia immunity is known to significantly diminish the response to recombinant replication-competent and replication-defective vaccinia-derived vectors [42], and pre-existing adenovirus immunity has been reported to diminish the magnitude of immune responses induced by adenovirus vectors [18], [55]. This effect potentially limits the ability to use multiple-dose regimens of homologous vectors. DNA immunization is not subject to anti-vector immunity and has the potential to establish an expanded precursor frequency of antigen-specific cellular immune responses in the majority of subjects. Therefore, by priming with DNA or other novel vectors it may be possible to lower the threshold for vector-based boosting of immune responses. The impact on modifying the threshold above which vector-based boosting occurs may be different for antibody responses compared to T cell responses, and this question will be addressed in future studies.

T cell subsets can be defined both by function and surface phenotype; the protective efficacy of a vaccine might be determined by which subsets are elicited. As we show, many of the T cell characteristics desirable for a vaccine against HIV were induced by DNA priming and rAd5 boosting. A high proportion of antigen-specific CD4 and CD8 T cells express CD127, suggesting that they are long-lived memory T cells capable of homeostatic maintenance – substantiated by the long term stability of the response. Further, the proportion of cells that express PD-1 declined over time (and the proportion co-expressing CD57^+^ was relatively low), consistent with maintenance of cells that can proliferate following antigenic challenge. Finally, we observed a mixture of T_CM_ and T_EM_/T_EF_, providing a balance of self-renewing cells and cells capable of a rapid effector response.

The combination of DNA priming and rAd5 boosting increased the polyfunctionality of the vaccine-induced T cell response (Figure 3). Although the biological significance of polyfunctional T cell responses in humans is unknown, *in vivo* animal model studies show that such qualitative immunological differences can impact upon vaccine-elicited protection [36]. Polyfunctional cells are thought to be more efficacious for two reasons: first, they bring to bear, on each target cell, a wider range of effector functions simultaneously (as opposed to requiring the simultaneous recruitment of multiple effector cells). Second, each polyfunctional cell expresses as much as an order of magnitude more of certain effector functions (such as IFN-γ or TNF; Figure S1). Ultimately, the value of vaccine-induced immune responses, and whether they achieve sufficient magnitude, functionality, breadth, and durability in the right location to impact the outcome of HIV infection, can only be determined in the setting of a clinical study evaluating efficacy.

An efficacious T cell vaccine against HIV will need to cover the extensive sequence diversity inherent in the current epidemic. While there are many ways to evaluate the breadth of a T cell response [56], [57], [58], we chose to take a rigorous approach by mapping individual epitopes targeted by the vaccine-induced response, and evaluating the ability of the epitope-specific T cells to recognize circulating clade variants. We found that there was wide variation in the number of epitopes targeted (1–10 epitopes, Table 2), most were in Env and Gag (Table 2); and the T cell responses against those epitopes recognized all or most major clade variants (Figure 5). It is unclear whether it is better for a vaccine to elicit multiple strain-specific T cell responses as opposed to fewer broadly cross-reactive responses to selected epitopes in which mutations incur a fitness cost to the virus. It is possible that the inclusion of three separate Env antigens in the vaccine focused the T cell response to common sequences within the three antigens. This could have decreased the number of epitopes targeted, but increased the likelihood of cross-clade recognition by the response.

Epitope-specific responses may vary in their *in vitro* and *in vivo* impact upon HIV replication [59]; a vaccine would ideally elicit responses to those epitopes known to be associated with better viral control. Most prominent among these is the HLA B57-restricted response to GagTW10 [12], [13], [60], [61]. It is encouraging that this response was elicited by the vaccine in both volunteers who express HLA B57 (Table 2). Indeed the GagTW10 functional avidity and pattern of clade variant recognition in these two volunteers was similar to what has been reported in HLA B57-expressing long-term non-progressors (Figure 5 and unpublished results). The basis for the relative response hierarchy between antigens (EnvA  =  EnvB > Gag  =  EnvC > Pol) is not known.

In the “Step” study (HVTN 502) evaluating the efficacy of repeated homologous boosting with the Merck rAd5 vector expressing Gag, Pol, and Nef, it was found that induction of Gag- and Pol-specific CD8^+^ T cells was not sufficient to prevent HIV infection or result in lower set-point viral load levels in men who became infected despite vaccination. Surprisingly, in men who were uncircumcised or had pre-existing neutralizing antibodies against Ad5, the repeated homologous rAd5 immunization increased the risk of HIV infection above that of placebo recipients [62]. The underlying biological mechanism for increased infection rates is unknown, but the results have called into question the potential role for rAd5 vectors in particular, and the value of vaccine-induced T cell mediated immunity for HIV in general. In contrast, a Phase III study (RV144) evaluating a recombinant canarypox vector expressing Env, Gag, and parts of Pol and Nef (vCP1521) in combination with a subunit glycoprotein (rgp120) in 16,402 volunteers in a general population cohort in Thailand showed a 31.2% reduction in acquisition [63]. The failure of the Merck rAd5 vector and partial success of the ALVAC/rgp120 prime-boost approach has highlighted the need for a greater depth of understanding of vector biology, as well as the importance of ongoing discovery efforts to define basic aspects of HIV immunity and pathogenesis [64], [65].

### Limitations and Conclusions

The major limitation of this study was its size. Only 14 subjects were evaluated by this intensive approach. The analysis required a large number of PBMCs and apheresis of subjects at key time points. The significant laboratory and personnel resources required also limited the study size. Another limitation was that boosting of subjects previously primed with DNA was opportunistic. Therefore, the study was not randomized and the prime-boost interval was variable. The interval was longer than that utilized in subsequent randomized trials.

In light of the concerning outcome of the Step study and encouraging results from the RV144 Phase III trial, it is important to expand our understanding of effector T cell function as it relates to HIV immunity. It will be critical to develop more sophisticated assays for future efficacy trials to assure that antibody and T cell functions associated with either favorable or adverse outcomes can be defined. Better assays are needed to assess proliferative capacity and kinetics of response, breadth and depth of response, functionality of response including both cytolytic and noncytolytic virus suppression activity, and localization of immune responses beyond just the blood compartment. These approaches in combination with more detailed assessment of antibody function, host genetics, breakthrough viral sequences, and a systems biology approach to analyze the transcriptional and proteomic profile associated with HIV immunity should be applied to future efficacy evaluations in humans to advance the goal of vaccine development.

The 6-plasmid DNA/rAd5 vector prime-boost combination has recently been evaluated in larger multicenter, international studies. Based on preclinical data showing a survival benefit in SIV-challenged macaques, favorable immunogenicity profile with different specificity and composition than that induced by the Merck vaccine, favorable safety profile in clinical studies to date, design features of the rAd5 vector backbone that differ from the Merck rAd5 vector, and the different antigen content and vaccine schedule, the heterologous prime-boost combination of DNA/rAd5 is being evaluated in a Phase II test-of-concept efficacy trial designed to determine whether the frequency, magnitude, functionality, breadth and durability of the vaccine-induced T cell response is sufficient to impact HIV-1 infection and/or disease progression.

## Supporting Information

Table S1 Subject demographics.
(0.04 MB DOC)

Table S2 Subjects were monitored throughout the study with physical and laboratory assessments by clinicians, as well as subject self-assessment for local (pain, swelling or redness) and systemic symptoms (fever, malaise, myalgia, headache, chills, nausea) by diary cards for 5 days after immunization. Adverse events were assessed for severity by using a pre-approved table using a 0–5 point grading scale and coded with the Medical Dictionary for Regulatory Activities (MedDRA). Thirteen of 14 subjects (93%) had “mild” local reactogenicity and 1 (7%) had a “moderate” reaction. One VRC 009 subject developed local erythema on Day 3 after the rAd5 vector boost, which peaked on Day 5 at 16×7 cm and resolved by Day 9. Five subjects (36%) had no systemic reactogenicity, 3 (21%) had mild symptoms, and 6 (43%) had moderate systemic reactions. A common symptom complex included malaise, headache and myalgia, sometimes with fever, within the first 24 hours after injection as previously described [18].
(0.06 MB DOC)

Table S3 Two subjects, who had previously received the 6-plasmid DNA, had a positive HIV EIA by an Abbott commercial diagnostic kit at the time of rAd5 vector boost; all other subjects tested negative. By six weeks after the booster rAd5 vector injection, all 14 subjects tested positive by the Abbott EIA; 6 (43%) were Western blot (WB) indeterminant and 8 (57%) were WB positive. All were confirmed uninfected by Roche RNA PCR testing, showing that the vaccine stimulated antibody responses to HIV gene products in approximately half of the subjects in the absence of infection. Seropositivity persisted through 24 weeks post rAd5 vector boost.
(0.03 MB DOC)

Figure S1 (A) Polyfunctional T cells are optimized for effector function, as shown by the amount of cytokine secreted on a per-cell basis. The top two graphs show histograms of IFN-gamma expression from cells producing only IFN-gamma (blue), or those that make two cytokines (green), three cytokines (orange), or four cytokines (red). Each Polyfunctional T cell elicited by the vaccine makes, on average, 30-fold more IFN-gamma than monofunctional T cells (MFI of 57,400 vs. 1,850). (B) The distribution of MFI for IFN-gamma (top) or TNF (bottom) showing that polyfunctional cells are highly optimized to produce both cytokines. MFIs were calculated only for subsets comprised of at least 10 events; hence the limited number of data points in some categories.
(0.55 MB TIF)

Figure S2 Envelope-specific ELISA antibody responses in subjects 4 weeks after the third dose of DNA or after a single dose of rAd5 vaccine only compared to peak response at 4–6 weeks following rAd5 vector boosting. Data for the rAd5 vaccine only group comes from protocol VRC 006 (18). Bars represent medians and one standard deviation.
(0.09 MB TIF)

Diagram S1 The Consort E-Flowchart VRC 009/010 studies.
(0.03 MB DOC)

Checklist S1 CONSORT Checklist
(0.19 MB DOC)

Protocol S1 Trial Protocol
(0.67 MB PDF)

Protocol S2 Trial Protocol
(0.56 MB PDF)
