# Supplementary material for: Priming Immunization with DNA Augments Immunogenicity of Recombinant Adenoviral Vectors for Both HIV-1 Specific Antibody and T-Cell Responses
Source: PLoS One. 2010 Feb 2;5(2):e9015. doi: 10.1371/journal.pone.0009015 (PMC2814848; doi:10.1371/journal.pone.0009015)
Supplement: Table S1 — Subject demographics. (0.04 MB DOC) [file pone.0009015.s001.doc]

**Table S**1. Subject Demographics

| **Category** | **Sub-category** | **VRC 009 (N=10)** | **VRC 010 (N=4)** | **VRC 006 (N=10)** |
| --- | --- | --- | --- | --- |
| GENDER | Male | 6 (60%) | 3 (75%) | 6 (60%) |
|  | Female | 4 (40%) | 1 (25%) | 4 (40%) |
| AGE | Mean (S.D.) | 30.0 (5.6) | 24.8 (6.9) | 27.4 (6.0) |
|  | Range | [23, 40] | [20, 35] | [21, 37] |
| RACE | American Indian/Alaskan Native | 0 | 0 | 0 |
|  | Asian | 0 | 0 | 0 |
|  | Black or African American | 0 | 0 | 0 |
|  | Native Hawaiian or other Pacific Islander | 0 | 0 | 0 |
|  | White | 9 (90%) | 4 (100%) | 9 (90%) |
|  | Multiracial | 1 (10%) | 0 | 1 (10%) |
| ETHNICITY | Non-Hispanic/Latino | 10 (100%) | 3 (75%) | 9 (90%) |
|  | Hispanic/Latino | 0 | 1 (25%) | 1 (10%) |
| BMI | Mean (S.D.) | 24.4 (4.2) | 32.2 (9.6) | 27.7 (6.8) |
|  | Range | [19.8, 33.2] | [23.4, 45.8] | [16.3, 37.9] |
