# Supplementary material for: Priming Immunization with DNA Augments Immunogenicity of Recombinant Adenoviral Vectors for Both HIV-1 Specific Antibody and T-Cell Responses
Source: PLoS One. 2010 Feb 2;5(2):e9015. doi: 10.1371/journal.pone.0009015 (PMC2814848; doi:10.1371/journal.pone.0009015)
Supplement: Table S2 — Subjects were monitored throughout the study with physical and laboratory assessments by clinicians, as well as subject self-assessment for local (pain, swelling or redness) and systemic symptoms (fever, malaise, myalgia, headache, chills, nausea) by diary cards for 5 days after immunization. Adverse events were assessed for severity by using a pre-approved table using a 0–5 point grading scale and coded with the Medical Dictionary for Regulatory Activities (MedDRA). Thirteen of 14 subjects (93%) had “mild” local reactogenicity and 1 (7%) had a “moderate” reaction. One VRC 009 subject developed local erythema on Day 3 after the rAd5 vector boost, which peaked on Day 5 at 16×7 cm and resolved by Day 9. Five subjects (36%) had no systemic reactogenicity, 3 (21%) had mild symptoms, and 6 (43%) had moderate systemic reactions. A common symptom complex included malaise, headache and myalgia, sometimes with fever, within the first 24 hours after injection as previously described [18]. (0.06 MB DOC) [file pone.0009015.s002.doc]

**Table S2. Local and systemic reactogenicity**

| **SYSTEMIC SYMPTOMS  Intensity** | **VRC 009/010 (N=14)**  **N (%)** |
| --- | --- |
| MALAISE |  |
| None | 7 (50) |
| Mild | 4 (28.6) |
| Moderate | 3 (21.4) |
| MYALGIA |  |
| None | 7 (50) |
| Mild | 3 (21.4) |
| Moderate | 4 (28.6) |
| HEADACHE |  |
| None | 8 (57.1) |
| Mild | 3 (21.4) |
| Moderate | 3 (21.4) |
| CHILLS |  |
| None | 8 (57.1) |
| Mild | 3 (21.4) |
| Moderate | 3 (21.4) |
| NAUSEA |  |
| None | 9 (64.3) |
| Mild | 5 (35.7) |
| Moderate | 0 |
| TEMPERATURE |  |
| None | 12 (85.7) |
| Mild | 2 (14.3) |
| Moderate | 0 |
| ANY SYSTEMIC SYMPTOM |  |
| None | 5 (35.7) |
| Mild | 3 (21.4) |
| Moderate | 6 (42.9) |

| **LOCAL SYMPTOMS  Intensity** | **VRC 009/010 (N=14)**  **N (%)** |
| --- | --- |
| PAIN/TENDERNESS |  |
| None | 0 |
| Mild | 13 (92.9) |
| Moderate | 1 (7.1) |
| SWELLING |  |
| None | 10 (71.4) |
| Mild | 4 (28.6) |
| Moderate | 0 |
| REDNESS |  |
| None | 10 (71.4) |
| Mild | 4 (28.6) |
| Moderate | 0 |
| ANY LOCAL SYMPTOM |  |
| None | 0 |
| Mild | 13 (92.9) |
| Moderate | 1 (7.1) |
