# Supplementary material for: Priming Immunization with DNA Augments Immunogenicity of Recombinant Adenoviral Vectors for Both HIV-1 Specific Antibody and T-Cell Responses
Source: PLoS One. 2010 Feb 2;5(2):e9015. doi: 10.1371/journal.pone.0009015 (PMC2814848; doi:10.1371/journal.pone.0009015)
Supplement: Table S3 — Two subjects, who had previously received the 6-plasmid DNA, had a positive HIV EIA by an Abbott commercial diagnostic kit at the time of rAd5 vector boost; all other subjects tested negative. By six weeks after the booster rAd5 vector injection, all 14 subjects tested positive by the Abbott EIA; 6 (43%) were Western blot (WB) indeterminant and 8 (57%) were WB positive. All were confirmed uninfected by Roche RNA PCR testing, showing that the vaccine stimulated antibody responses to HIV gene products in approximately half of the subjects in the absence of infection. Seropositivity persisted through 24 weeks post rAd5 vector boost. (0.03 MB DOC) [file pone.0009015.s003.doc]

**Table S3: Vaccine-induced seropositivity in commercial test kits**

| **Study Week** | **HIV-1 RNA PCR** | | **Abbott EIA Results** | | ***Commercial Western Blot Results** | | | |
| --- | --- | --- | --- | --- | --- | --- | --- | --- |
|  | Negative | Positive | Negative | Positive | Negative | Indeterminate | Positive | Uninterpretable |
| Week 0 | 14 | 0 | 12 | 2 | 1 | 1 | 0 | 0 |
| Week 6 | 14 | 0 | 0 | 14 | 0 | 6 | 8 | 0 |
| Week 24 | 14 | 0 | 0 | 14 | 0 | 6 | 5 | 3 |
| *Western Blot is done only for positive ELISA results  Two VRC 010 subjects began the study with reactive EIA from the DNA vaccine alone | | | | | | | | |
