# Supplementary figures and images for: Priming Immunization with DNA Augments Immunogenicity of Recombinant Adenoviral Vectors for Both HIV-1 Specific Antibody and T-Cell Responses
Source: PLoS One. 2010 Feb 2;5(2):e9015. doi: 10.1371/journal.pone.0009015 (PMC2814848; doi:10.1371/journal.pone.0009015)

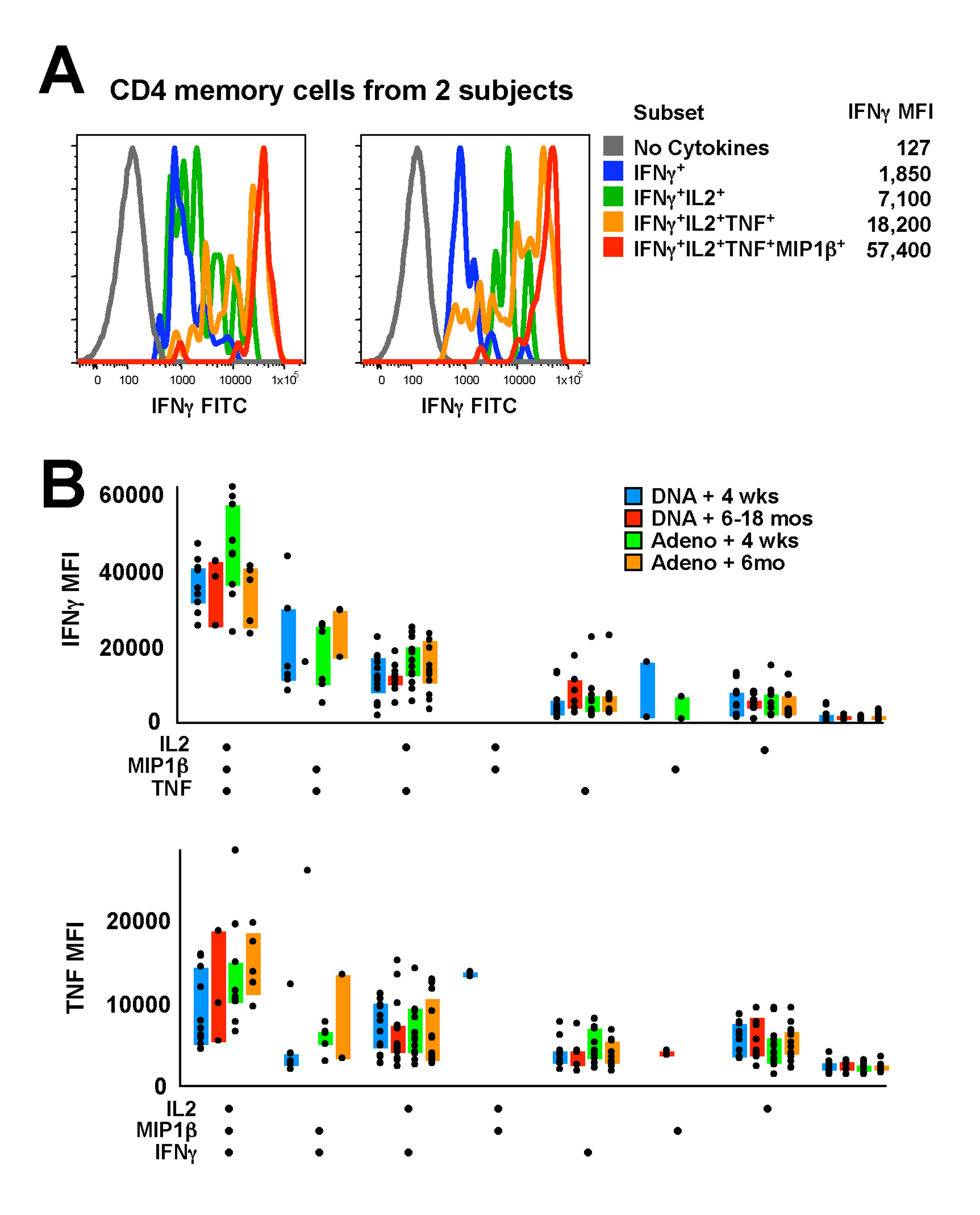

Supplement: Figure S1 — (A) Polyfunctional T cells are optimized for effector function, as shown by the amount of cytokine secreted on a per-cell basis. The top two graphs show histograms of IFN-gamma expression from cells producing only IFN-gamma (blue), or those that make two cytokines (green), three cytokines (orange), or four cytokines (red). Each Polyfunctional T cell elicited by the vaccine makes, on average, 30-fold more IFN-gamma than monofunctional T cells (MFI of 57,400 vs. 1,850). (B) The distribution of MFI for IFN-gamma (top) or TNF (bottom) showing that polyfunctional cells are highly optimized to produce both cytokines. MFIs were calculated only for subsets comprised of at least 10 events; hence the limited number of data points in some categories. (0.55 MB TIF) [file pone.0009015.s004.tif]

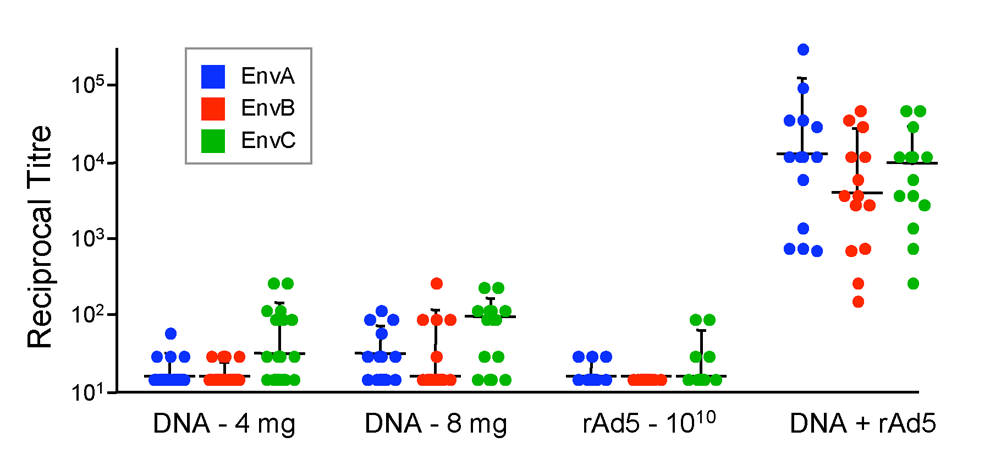

Supplement: Figure S2 — Envelope-specific ELISA antibody responses in subjects 4 weeks after the third dose of DNA or after a single dose of rAd5 vaccine only compared to peak response at 4–6 weeks following rAd5 vector boosting. Data for the rAd5 vaccine only group comes from protocol VRC 006 (18). Bars represent medians and one standard deviation. (0.09 MB TIF) [file pone.0009015.s005.tif]
