# Supplementary material for: Priming Immunization with DNA Augments Immunogenicity of Recombinant Adenoviral Vectors for Both HIV-1 Specific Antibody and T-Cell Responses
Source: PLoS One. 2010 Feb 2;5(2):e9015. doi: 10.1371/journal.pone.0009015 (PMC2814848; doi:10.1371/journal.pone.0009015)
Supplement: Diagram S1 — The Consort E-Flowchart VRC 009/010 studies. (0.03 MB DOC) [file pone.0009015.s006.doc]

**The Consort E-Flowchart VRC 009/010 Studies**

DNA Vaccine Studies:

VRC 004 (n=35) in 4 mg or 8 mg prime groups

VRC 007 (n=15) in 4 mg prime group

Consented to be assessed for rAd5 boost vaccine study eligibility (n=20)

n=11 from VRC 004 assessed for VRC 009

n=9 from VRC 007 assessed for VRC 010

**Allocation**

**Analysis**

**Follow-Up**

**Enrollment**

Excluded (n= 6)

Not meeting inclusion criteria (n=5)

Declined to participate (n=1)

Completed follow-up (n=14)

Lost to follow-up (n= 0)

Discontinued intervention (n= 0)

Allocated to intervention (n=14)

n=10 from VRC 004 allocated to VRC 009

n= 4 from VRC 007 allocated to VRC 010

Received allocated intervention (n=14)

Did not receive allocated intervention (n= 0)

Give reasons

Analyzed (n=14)

Excluded from analysis (n= 0)

Is it Randomized? No
