# Supplementary material for: Priming Immunization with DNA Augments Immunogenicity of Recombinant Adenoviral Vectors for Both HIV-1 Specific Antibody and T-Cell Responses
Source: PLoS One. 2010 Feb 2;5(2):e9015. doi: 10.1371/journal.pone.0009015 (PMC2814848; doi:10.1371/journal.pone.0009015)
Supplement: Protocol S2 — Trial Protocol (0.56 MB PDF) [file pone.0009015.s009.pdf]

Version 1.0
March 3, 2005

VACCINE RESEARCH CENTER

Protocol VRC 010
(NIH 05-I-0140)

A Phase I Clinical Trial to Evaluate the Safety and Immunogenicity of a Booster Dose of a Recombinant Multiclade HIV-1 Adenoviral Vector Vaccine, VRC-HIVADV014-00-VP, in Uninfected Subjects who were Previously Immunized with VRC-HIVDNA016-00-VP in VRC 007 (04-I-0254)

Vaccines Provided by
Vaccine Research Center/NIAID/NIH

Clinical Trial Sponsored by:
National Institute of Allergy and Infectious Diseases (NIAID)
Vaccine Research Center (VRC)
Bethesda, Maryland

IND Sponsored by:
National Institute of Allergy and Infectious Diseases
Division of AIDS (DAIDS)
Bethesda, Maryland

BB-IND 12326 - held by DAIDS

Principal Investigator:

Andrew Catanzaro, M.D.,
Vaccine Research Center, National Institute of Allergy and Infectious Diseases (NIAID)
National Institutes of Health (NIH)
Bethesda, MD 20892

IRB Initial Review Date: February 22, 2005
VRC Protocol: 010

TABLE OF CONTENTS

Page
Précis 5
1. INTRODUCTION AND RATIONALE 6
1.1 HIV-1: Etiology, Disease Course, and Epidemiology 6
1.2 Rationale for VRC 010: Adenoviral Vector Booster Vaccination in Subjects with DNA Vaccine Priming Vaccinations 6
1.3 Previous Human Experience with VRC-HIVADV014-00-VP 9
1.4 Measures of Immunogenicity 10
1.5 Apheresis Collection of PBMC for Week 4 Immunogenicity 11
2. STUDY VACCINE: VRC-HIVADV014-00-VP 11
2.1 Description of the Study Agent VRC-HIVADV014-00-VP 11
2.1.1 Production of the *gag-pol* Adenoviral Vector 13
2.1.2 Production of the *env* Adenoviral Vectors 13
2.2 Preparation of the Bulk Plasmid and Final Products 14
2.3 Release Criteria 14
2.4 Preclinical Safety Studies 15
2.4.1 Biodistribution Study of VRC-HIVADV014-00-VP in Rabbits 15
2.4.2 Toxicology Study of VRC-HIVADV014-00-VP Alone and in a Prime-Boost Regimen with the DNA Vaccine, VRC-HIVDNA009-00-VP 16
2.5 Preclinical Immunogenicity Studies 18
2.5.1 Immune Responses to VRC-HIVADV014-00-VP Alone in Mice 19
2.5.2 Immune Responses to VRC-HIVDNA009-00-VP Prime Followed by VRC-HIVADV014-00-VP Boost in Rabbits 19
2.5.3 Immune Responses to VRC-HIVADV014-00-VP Alone in Cynomolgus Macaques (VRC 03-060) 20
2.5.4 Immune Responses to VRC-HIVDNA009-00-VP Prime Followed by VRC-HIVADV014-00-VP Boost in Cynomolgus Macaques (VRC-02-035) 20
3. STUDY OBJECTIVES 21
3.1 Primary Objectives 21
3.2 Secondary Objectives 21
3.3 Exploratory Analyses 21
4. STUDY DESIGN AND METHODS 21
4.1 Study Population 22
4.1.1 Inclusion Criteria 22
4.1.2 Exclusion Criteria 24
4.2 Subject Monitoring: Schedule of Evaluations 25
4.2.1 Screening 26
4.2.2 Day 0 Through Week 24 Follow-Up 27
4.3 Monitoring for HIV Infection 29
4.4 Intercurrent HIV Infection 29
4.5 Concomitant Medications 29
4.6 Criteria for Withdrawing a Subject from Study Participation 30
4.7 Criteria for Stopping Study 30
5. ADVERSE EVENT REPORTING 31

5.1 Adverse Events 31
5.2 Serious Adverse Events 31
5.3 Adverse Event Reporting to the IND Sponsor....................31
5.4 Adverse Event Reporting to the Institutional Review Board....................33
5.5 Serious Adverse Event Reporting to the Institutional Biosafety Committee 33
6. STATISTICAL CONSIDERATIONS....................33
6.1 Overview....................33
6.2 Objectives 33
6.3 Endpoints 33
6.3.1 Safety 33
6.3.2 Immunogenicity 34
6.3.3 Social Impacts....................34
6.4 Sample Size and Accrual Guidelines....................34
6.4.1 Power Calculations for Safety 34
6.5 Statistical Analysis....................35
6.5.1 Analysis Variables 35
6.5.2 Safety Analysis 36
6.5.3 Immunogenicity Analysis 36
6.5.4 Social Impact analysis....................36
6.5.5 Interim analyses 36
7. PHARMACY PROCEDURES 37
7.1 Study Agent Supply 37
7.2 Study Agent Preparation....................37
7.3 Study Agent Labeling 37
7.4 Study Agent Accountability....................38
7.4.1 Documentation....................38
7.4.2 Disposition 38
8. HUMAN SUBJECT PROTECTIONS AND ETHICAL OBLIGATIONS....................38
8.1 Informed Consent....................38
8.2 Risks and Benefits....................38
8.2.1 Risks....................38
8.2.2 Benefits 40
8.3 Institutional Review Board 40
8.4 Subject Confidentiality 40
8.5 Plan for Use and Storage of Biological Samples 41
8.6 Subject Identification and Enrollment of Study Participants....................41
8.6.1 Participation of Children....................41
8.7 Compensation Plan for Subjects 41
8.8 Safety Monitoring 41
9. ADMINISTRATIVE AND LEGAL OBLIGATIONS....................41
9.1 Protocol Amendments and Study Termination....................41
9.2 Study Documentation and Storage....................42
9.3 Data Collection and Protocol Monitoring....................42
9.3.1 Data Collection 42
9.3.2 Protocol Monitoring Plan....................42
9.4 Language....................43

9.5 Policy Regarding Research-Related Injuries 43
10. REFERENCES 44

Appendices

I Informed Consent Form 48
II Contact Information 64
III Schedule of Evaluations 66
IV Table for Grading Severity of Adverse Events 68

Précis

Protocol VRC 010: A Phase I Clinical Trial to Evaluate the Safety and Immunogenicity of a Booster Dose of a Recombinant Multiclade HIV-1 Adenoviral Vector Vaccine, VRC-HIVADV014-00-VP, in Uninfected Subjects who were Previously Immunized with VRC-HIVDNA016-00-VP in VRC 007 (04-I-0254)

Study Design: This is a Phase I open-label study to examine safety, tolerability and immune response of a multiclade HIV adenoviral vector vaccine as a booster vaccination in uninfected adults. The hypothesis is that this vaccine will be safe as a booster vaccine and elicit immune responses to HIV. The primary objective is to evaluate the safety and tolerability of a VRC-HIVADV014-00-VP booster vaccination in uninfected subjects who previously received 3 injections of VRC-HIVDNA016-00-VP. The secondary objectives include immunogenicity evaluations, adenovirus serotype 5 (Ad5) antibody titers, and social impacts. Exploratory evaluations include epitope mapping and other immunogenicity evaluations.

Product Description: VRC-HIVADV014-00-VP (rAd) is a recombinant product composed of 4 adenoviral vectors (in a 3:1:1:1 ratio) that encode the HIV-1 Gag/Pol polyprotein from clade B and HIV-1 Env glycoproteins from clades A, B, and C, respectively.

Subjects: Healthy adult volunteers who previously received three injections of VRC-HIVDNA016-00-VP at a dosage of 4 mg in the VRC 007 study (04-I-0254). The subjects in these groups were between 20 and 36 years old at time of enrollment in VRC 007; those who participate in VRC 010 will be no more than 36 weeks older at the time of enrollment into VRC 010.

Study Plan: Up to fourteen volunteers will receive one 1 mL injection of the study agent at a dosage of 10^{10} particle units (PU) intramuscularly (IM) in a deltoid muscle. Safety and immunogenicity will be evaluated by follow-up visits over the subsequent 24 weeks. The peripheral blood mononuclear cell (PBMC) sample for immunogenicity studies collected at Week 4 after vaccination will be obtained by apheresis if the subject is willing and eligible for apheresis and an apheresis appointment can be conveniently scheduled in the interval specified; otherwise PBMCs will be obtained from 80 mL blood collected by phlebotomy.

Study Duration: Subjects will be evaluated at 6 or more clinical visits for 24 weeks after the study injection.

Study Endpoints: The primary endpoint is safety of the vaccine administered at a dose of 10^{10} PU by IM injection. Secondary endpoints are immunogenicity as indicated by HIV-specific antibody through Week 4, cellular immune responses through Week 6, Ad5 antibody titer at Week 0 and Week 4 and social impact at Week 24. Exploratory analyses include immunogenicity at Weeks 12 and 24, Ad5 antibody titer at Week 24 and epitope mapping of the $CD8^+$ and $CD4^+$ T cell responses at Week 4.

1. INTRODUCTION AND RATIONALE

1.1 HIV-1: ETIOLOGY, DISEASE COURSE, AND EPIDEMIOLOGY

The Centers for Disease Control and Prevention (CDC) estimate that in the United States, 850,000 to 950,000 people are living with human immunodeficiency virus (HIV) infection and approximately 25% are unaware of their infection [1]. Worldwide, the rate of new HIV infections continues to increase at an unacceptably high level. Although new AIDS diagnoses and deaths have fallen significantly in developed countries since the advent of highly active antiretroviral therapy (HAART), in the developing world the HIV/AIDS epidemic continues to accelerate. The global impact of the epidemic is staggering. According to the Joint United Nations Programme on HIV/AIDS and the World Health Organization, as of the end of 2002, 40-42 million people were estimated to be living with HIV/AIDS, with 95% of the global total residing in the developing world [2, 3]. Worldwide there were an estimated 2.5-3.5 million deaths due to HIV/AIDS in 2003 [2] and there have been as many as 30 million deaths as a result of HIV infection since the beginning of the epidemic [3]. Beyond the human tragedy of HIV/AIDS, the costs of the epidemic pose a significant impediment to the economic growth and political stability of many countries. In developing countries and in segments of the U.S. population, anti-HIV therapies are frequently beyond financial reach. Accordingly, effective, low-cost tools for HIV prevention, such as a vaccine, are urgently needed to bring the HIV epidemic under control. For this reason, the Vaccine Research Center (VRC) and Division of AIDS (DAIDS) at the National Institute of Allergy and Infectious Diseases (NIAID) of the National Institutes of Health (NIH) are committed to the development of safe and effective vaccines to prevent HIV infection and AIDS worldwide.

1.2 RATIONALE FOR VRC 010: ADENOVIRAL VECTOR BOOSTER VACCINATION IN SUBJECTS WITH DNA VACCINE PRIMING VACCINATIONS

VRC 010 will provide the first opportunity to evaluate the safety of a multiclade adenoviral vector (rAd) booster vaccination with VRC-HIVADV014-00-VP in subjects who have had three priming vaccinations with a 4 mg dose of a multiclade deoxyribonucleic acid (DNA) vaccine (VRC-HIVDNA016-00-VP) prior to the booster vaccination. This study will provide the first immunological data with regard to boosting subjects whose T cell responses to this DNA vaccine are already characterized. Also by obtaining a large number of Week 4 peripheral blood mononuclear cells (PBMCs) by apheresis this single site study will enable extensive phenotyping and epitope definition of the boosting response. These data will be compared to the phenotype of the response to selected epitopes after DNA priming (prior to the rAd boost). VRC 010 will provide booster vaccination safety and immunological data that are uniquely valuable in developing an understanding of a DNA prime-rAd boost vaccination strategy for prevention of HIV. VRC 010 data will also be complementary to the safety and immunogenicity data that will be provided by VRC 008, a prime-boost study of the same two vaccines in healthy subjects 18-50 years old who will be randomized to be boosted with either a 10^{10} or 10^{11} particle units (PU) dose of the adenoviral vector vaccine. VRC 007 includes 15 subjects and 14 of these completed the vaccination schedule with the DNA vaccine, VRC-HIVDNA016-00-VP. Although many fewer than these 14 VRC 007 subjects who completed the 3-injection vaccination schedule may be willing and eligible to participate in VRC 010, if even a few subjects enroll in VRC 010, this will provide early valuable preliminary data on the safety and immunogenicity of the prime-

boost regimen. About the same time that VRC 010 opens to accrual and the administration of booster vaccinations with the adenoviral vector vaccine begins, the subjects enrolling in study VRC 008 will just be starting to receive the priming vaccinations with the DNA vaccine. The information from these first few booster vaccinations in VRC 010 will be available about 6 months before the VRC 008 subjects get to the booster part of the study. These preliminary safety and immunogenicity data will help determine if any adjustments need to be made in the VRC 008 study plan, as well as other prime-boost protocols under development that will be conducted as extramural, multicenter trials.

By the time VRC 010 opens to enrollment, both an intramural study, VRC 009, and an extramural study of the HIV Vaccine Trials Network (HVTN), HVTN 057, will be providing the first safety and immunogenicity data on a prime-boost regimen that includes a similar DNA vaccine and the same adenoviral vector vaccine. The subjects who will be in VRC 009 were primed with 4 mg or 8 mg of a similar DNA plasmid vaccine (VRC-HIVDNA009-00-VP) and will be boosted with 10^{10} PU rAd vaccine more than a year later. The subjects who are in HVTN 057 were primed with either two or three injections of 4 mg of VRC-HIVDNA009-00-VP (or placebo) and are boosted with 10^{10} PU rAd vaccine (or placebo) 6-9 months later.

Preclinical immunogenicity studies have used a boost interval that is thought to be a timepoint where the primary immune response has reached the stage where memory T cells are well established. DNA priming is thought to establish a memory pool with enhanced Th1 immune response. One purpose of the adenoviral vectored vaccine boost is to effectively expand the memory $CD8^+$ T cell pool. In VRC 010, the booster vaccination will be administered as close as possible to 24 weeks after the first DNA priming vaccination was administered in VRC 007. However, a window of between 24 and 36 weeks after the first DNA priming vaccination is permitted in order to accommodate study subject personal schedules in conjunction with the schedule limitations that may occur related to study site facilities.

A major challenge in the design of HIV vaccines is to identify and target viral structures that are the critical determinants for protective humoral and cellular responses across the widest possible range of diversity. The use of multivalent vaccines, containing a defined mixture of immunogens from a number of prevalent HIV subtypes might be a feasible approach to achieve broadly protective HIV vaccines. The World Health Organization UNAIDS HIV Vaccine Advisory Committee has recommended that candidate HIV vaccines be designed based upon the strains prevalent in the country in which trials are to be conducted [4]. This approach is the foundation for the design of the investigational vaccine VRC-HIVADV014-00-VP, which is composed of a combination of four rAd that incorporate HIV *gag, pol* and *env* genes. The Gag protein along with the highly conserved Pol protein are derived from a clade B strain of HIV-1; the Env proteins are derived from clades A, B and C, which together represent the viral subtypes responsible for about 90% of new HIV infections in the world [5]. The Vaccine Research Center, NIAID, NIH and the World Health Organization-Joint United Nations Programme on HIV/AIDS organized a meeting focused on the genetic diversity of HIV and strategies to develop vaccine candidates. A consensus was reached that generation of multiclade candidate vaccines is a high international scientific priority [6].

The intended use for the study vaccine VRC-HIVADV014-00-VP is as a preventive HIV-1 vaccine. This recombinant adenoviral (rAd) vaccine product is based on the concept of immunization by gene delivery. Recombinant adenoviral vector vaccines offer the positive attributes of immune stimulation inherent in live attenuated vaccines, without adjuvant, and

without using HIV-1 as the attenuated virus.

Cellular immune responses play a vital role in containing HIV-1 replication, are temporally associated with declining HIV-1 viremia in acute infection and are often present at high levels during chronic HIV-1 infection [7-11]. VRC-HIVADV014-00-VP was designed with the rationale that an effective preventive vaccine would have to stimulate strong virus-specific $CD8^+$ cytotoxic T-lymphocyte (CTL) immune responses. The importance of $CD8^+$ CTL in controlling SIV replication in rhesus monkeys has been directly established by studies in which *in vivo* depletion of $CD8^+$ T-cells resulted in a rapid and dramatic increase in plasma viremia [12, 13]. The importance of $CD4^+$ helper T lymphocyte responses in controlling HIV-1 replication has also been established, however in contrast to robust CTL responses, HIV-1-specific $CD4^+$ T lymphocyte responses are typically weak or absent in most chronically HIV-1-infected individuals, partly because HIV-1 preferentially infects and destroys HIV-specific $CD4^+$ T cells [14]. A vaccine strategy that can stimulate a balanced $CD4^+$ and $CD8^+$ response is most likely to control HIV-1 infection upon challenge.

Preclinical evidence indicates that VRC-HIVADV014-00-VP elicits cellular immune responses against HIV-1 in mice, rabbits and monkeys by direct gene delivery of immunogen-expressing HIV genes via rAd vectors. The preclinical immunogenicity studies suggest that a rAd vaccine containing genes that express Gag and Pol in combination with HIV envelope proteins may induce a significant immune response in humans. Preclinical studies also indicate that the immunogenicity of the vaccine is enhanced when it is used as a booster vaccine in a prime-boost regimen. Data from the preclinical studies with the investigational vaccine are summarized briefly in Section 2.5 of the protocol and in greater detail in the Investigator Brochure.

The major advantage of adenoviral vector immunization appears to be its efficacy in the induction of $CD8^+$ CTL responses, considered an important element in controlling HIV-1 viral replication [10, 12, 13, 15-18]. There is an additional safety feature in that, following entry into the target cells, the HIV-1 gene products will be produced without the production of infectious adenovirus. These gene products can be produced in cells that are not actively dividing. Studies in non-human primates have shown that replication-deficient serotype 5 adenovirus vectors (Ad5) can generate cellular immune responses against several viruses including HIV-1, simian-human immunodeficiency virus (SHIV) and Ebola [19-23]. Baboons immunized with 10^{11} particles of replication-deficient Ad5 have been shown to have strong cellular immune responses evidenced by Gag-specific T-cells that were quantified by enzyme-linked immunospot (ELISPOT) assay [22]. Rhesus macaques immunized with SIV Gag-based Ad5 vectors showed potent CTL responses that correlated with protection (reduced $CD4^+$ loss, contained acute and chronic viremia and reduced morbidity and mortality when challenged with a pathogenic strain of SIV) [20].

There is concern that because of the prevalence of pre-existing neutralizing antibodies to adenoviruses in the adult population, the utility of adenoviral vectors in humans may be limited. Merck Research Laboratories report that preclinical studies show priming for HIV-1 specific immunity using an adjuvant-formulated DNA vaccine followed with Ad5 vaccine boost generates levels of T-cell immune response that are comparable to those in naive animals receiving multiple high doses of Ad5 HIV-1 vaccines [22]. Cynomolgus macaques immunized with a combination DNA plasmid (a mixture of four DNA plasmids encoding glycoproteins from three Ebola strains and nucleoprotein from one strain) and boosted with a replication-deficient adenoviral vector encoding the glycoprotein resisted lethal viral challenge [21]. These studies

suggest that replication-defective adenoviral vectors, as part of a carefully crafted vaccine strategy, can elicit potent and protective T-cell immune responses that may control HIV-1 and other viral infections.

The safety of VRC-HIVADV014-00-VP is currently being evaluated in a Phase I study [VRC 006 (04-I-0172)] in which it is administered as a single agent. Protocol VRC 010, as well as the extramural, multicenter protocol (HVTN 057) and the intramural study VRC 009, are part of the next step in the clinical development plans for VRC-HIVADV014-00-VP. This involves evaluating the vaccine in a prime-boost regimen of three DNA priming vaccinations followed by a single rAd vaccine booster vaccination. Combination modality regimens using a DNA vaccine prime followed by a viral vector boost have shown promise in non-human primate models of HIV infection. Such regimens have the potential for raising high levels of immune responses. DNA vaccine priming followed by a recombinant viral vector boost with a modified vaccinia Ankara (rMVA) [24] or replication-deficient Ad5 [20] have been shown to attenuate a pathogenic SHIV infection in rhesus macaques, most likely by the generation of a $CD8^+$ CTL response.

1.3 PREVIOUS HUMAN EXPERIENCE WITH VRC-HIVADV014-00-VP

The prior human experience with live adenovirus vaccines [25-29] and recombinant adenoviral vector vaccines [30-53] was extensively reviewed in the investigational new drug application (IND), BB-IND 11661, that was submitted for VRC-HIVADV014-00-VP with the clinical trial VRC 006 (04-I-0172), as well as in BB-IND 11894, which includes two studies of a similar prime-boost regimen (HVTN 057 and VRC 009).

The Vaccine Research Center (VRC) is conducting VRC 006 (04-I-0172), "A Phase I Clinical Trial to Evaluate the Safety and Immunogenicity of a Recombinant Multiclade HIV-1 Adenoviral Vector Vaccine, VRC-HIVADV014-00-VP, in Uninfected Adult Volunteers." This is a randomized, placebo-controlled, double-blinded, dose escalation study to examine safety, tolerability and immune response following a single injection of VRC-HIVADV014-00-VP at a dose of 10^9 PU, 10^{10} PU, or 10^{11} PU. Each group includes 12 subjects (10 vaccine; 2 placebo).

VRC 006 was initiated on July 19, 2004 and the study completed enrollment of 36 subjects on November 10, 2004. The NIAID Intramural Data and Safety Monitoring Board (DSMB) reviewed the preliminary safety data through 14 days of follow-up prior to each dose escalation. The preliminary data indicate that the vaccine appears to be safe for healthy subjects at the three dose levels evaluated. The 10^9 PU and 10^{10} PU dose levels are associated with less reactogenicity than the 10^{11} PU dose level. In both the 10^9 and 10^{10} PU dose groups the local and systemic parameters recorded on the 5-day diary card were none to mild in severity and none of the subjects experienced fever. In the 10^{11} PU dose group, four subjects reported fever on Day 1 (3 mild and 1 moderate in severity). Each of the four subjects with fever also reported moderate headache on Day 1 and three of these subjects also reported at least one other moderate systemic parameter (malaise, myalgia, chills). Two subjects without fever reported at least one moderate systemic symptom (malaise, myalgia, nausea). One subject in the 10^{11} PU dose group reported moderate injection site pain; injection site reactogenicity was otherwise none or mild. As of January 31, 2005, there has been one serious adverse event (grade 4 seizure) and three adverse events of grade 2 (moderate) severity that are possibly related to vaccination. The study remains blinded to vaccine vs placebo injection assignments. The seizure occurred 64 days after study injection in a healthy subject in the 10^{11} PU dose group who had a history of a single seizure

three years prior to study enrollment. Given history of a prior seizure and timing of event more than two months after study injection, it seems unlikely that recent seizure is related to study agent. The grade 2 adverse events possibly related to study agent include: 1) asymptomatic neutropenia noted 21 days after study injection in a subject known to sometimes have asymptomatic low neutrophil counts; 2) diarrhea (duration one day) in a different subject on the third day after study injection and 3) steatohepatitis (fatty liver) diagnosed after extensive evaluation to identify the cause of a persistent grade 1 ALT (alanine aminotransferase) elevation that was noted starting 25 days after the study vaccination in a clinically asymptomatic subject. The impression after consulting with a hepatologist is that the condition likely existed prior to study enrollment. Contributing factors to the persistent grade 1 ALT may be alcohol consumption and recent weight gain. A diagnosis of steatohepatitis is overall considered to be a grade 2 condition, but as of 1/31/05 the liver function tests have not been more than grade 1 in severity. The safety data are expected to remain blinded until June 2005. The VRC 010 study proposes to use the 10^{10} PU dose for the booster vaccination.

HVTN 057 was recently initiated. In this placebo-controlled Phase I study a single adenoviral vector boost of VRC-HIVADV014-00-VP at 10^{10} PU (or placebo) will be administered to participants who complete the injection regimen in HVTN 052 with the DNA vaccine, VRC-HIVDNA009-00-VP (or placebo). The rAd boost will be given at an interval of 6-9 months after the initial DNA vaccine in HVTN 052. The first participant was enrolled on November 22, 2004. As of December 21, 2004, 11 participants have received their boost injection. Of these participants, 6 have experienced mild pain and/or tenderness at the injection site. There have been no other reports of local reactogenicity events. Five participants reported either mild or moderate systemic symptoms including headache, malaise and nausea. There have been no reports of fevers, no grade 3 events, and no serious adverse events.

VRC 009: VRC 009 is the second Phase I study of rAd as a booster vaccine. The first subject was enrolled into this open label study on January 28, 2005. Subjects who completed three vaccinations with 4 mg or 8 mg of VRC-HIVDNA009-00-VP in VRC 004 (03-I-0022) and who are willing and eligible will be enrolled to receive a 10^{10} PU booster vaccination at about 18-28 months after their first DNA vaccine injection.

1.4 MEASURES OF IMMUNOGENICITY

In this Phase I study, employing assays that evaluate CTL responses, as well as assays that evaluate antibody responses will provide a preliminary assessment of vaccine immunogenicity. The intracellular cytokine staining (ICS) assay is based upon previously published methods [54] and quantitates the frequency of $CD4^+$ and $CD8^+$ cells that produce the cytokines interferon-gamma or interleukin-2 in response to pools of overlapping peptides representing HIV antigens (Gag, Pol or Env) from specific HIV clades. These clade-specific peptides will also be used to detect T-cell responsiveness by an enzyme-linked immunospot (ELISPOT) assay modified from a previously published method [55]. In addition, an extensive $CD4^+$ and $CD8^+$ T cell analysis by epitope mapping is planned for subjects from whom sufficient PBMC are available [54, 56, 57].

Antibody responses to the individual HIV gene products contained within the vaccine will be evaluated using an enzyme-linked immunosorbent assay (ELISA) [58]. The ability of the vaccine to elicit neutralizing antibody against HIV-1 strains from clades A, B, and C will be

evaluated by a flow cytometric assay that measures the capacity of sera to block single round infection of individual PBMC [59].

The pre-existing and post-vaccination presence of adenovirus serotype 5 neutralizing antibody in study volunteers will be evaluated from frozen serum samples using a previously published method [60].

1.5 APHERESIS COLLECTION OF PBMC FOR WEEK 4 IMMUNOGENICITY

In VRC 010, whenever possible, the PBMC sample for immunogenicity studies collected at Week 4 (Day 28±3 days) after vaccination will be obtained by apheresis. When apheresis is not possible in this timeframe, 80 mL of blood will be collected by phlebotomy. About 4 weeks after vaccination is anticipated to be the peak of T cell response. Epitope mapping of Week 4 PBMC will provide detailed characterization of the vaccine-induced T cell response post-boosting. Sufficient PBMC to accomplish epitope mapping, as well as the other immunology studies at this timepoint, can be collected by apheresis. A two-pass apheresis procedure yields about 1×10^9 (1 billion) PBMC with only a 20 mL blood loss from the procedure, whereas an 80 mL phlebotomy yields about 8×10^7 (80 million) PBMC. Although all subjects will provide valuable information about the immunological response to the adenoviral vector booster vaccination, more extensive immunological analysis of a booster vaccination will be possible for those subjects who are willing and able to undergo apheresis about 4 weeks after the study vaccination.

Each apheresis procedure will be carried out by trained staff of the NIH Clinical Center Department of Transfusion Medicine (DTM) Apheresis Clinic under the supervision of the DTM medical staff in order to harvest PBMC. Apheresis will be done using automated cell separator devices. Whole blood is withdrawn from a venipuncture site in an antecubital vein at a rate of 50 to 80 mL/min and directed into the cell separator, where cellular and plasma fractions are separated by centrifugation. Mononuclear cells are harvested into a component bag, and the remaining red cells, platelets and plasma are returned to the subject via the same vein. Anticoagulation is achieved using citrate (ACD-A) or equivalent at a whole blood to anticoagulant ratio of 12:1. Maximum extracorporeal blood volume during the procedure ranges from 400 mL to 800 mL, depending on the subject's hematocrit. These procedures are carried out in such a way that the extracorporeal volume is no more than 15% of the blood volume in adults. These volumes are calculated using standard formulae in the DTM Apheresis Clinic. In this study, the procedure will usually involve two discontinuous-flow passes. Each pass takes about 30 minutes, processes about 500 mL of whole blood, and yields about 0.5×10^9 mononuclear cells. Thus, one to two hours are required to process 1 to 2 liters of blood and obtain about 1×10^9 to 2×10^9 leukocytes. The cell differential in the product is about 65% lymphocytes and 20% monocytes, with the remainder being granulocytes. The packed red cell loss during the procedure is about 20 mL.

2. STUDY VACCINE: VRC-HIVADV014-00-VP

2.1 DESCRIPTION OF THE STUDY AGENT VRC-HIVADV014-00-VP

The recombinant adenoviral vector product VRC-HIVADV014-00-VP (rAd) is a replication-deficient, combination vaccine containing four recombinant serotype 5 adenoviral vectors that

was developed by the Vaccine Research Center, NIAID, NIH. These vectors contain gene sequences that code for clade B HIV-1 Gag and Pol as well as clade A, clade B, and clade C Env protein. *In vivo* expression by these vectors produces immunogens that induce an immune response against HIV. The envelope genes were chosen as representative primary isolates from each of the three clades.

The process for constructing the four VRC-HIVADV014-00-VP recombinant adenoviral vectors is based upon a rapid vector construction system (AdFAST™, GenVec, Inc.) used to generate adenoviral vectors that express the four HIV antigens gp140(A), gp140(B)dv12, gp140(C) and GagPol(B) driven by the cytomegalovirus (CMV) immediate-early promoter. Manufacturing is based upon production in a proprietary cell line (293-ORF6), yielding adenoviral vectors that are replication deficient. The vectors are purified using cesium chloride (CsCl) centrifugation. The product is formulated as a sterile liquid injectable dosage form for intramuscular injection.

The GV11 adenoviral backbone was chosen to reduce the risk of replication-competent adenovirus (RCA) generation during clinical production. The GV11 backbone contains deletions of two essential regions, E1 and E4, as well as a partial E3 deletion. These deletions render the vaccine product replication-deficient. The generation of RCA would require two independent recombination events in a single adenovirus genome, predicted to be an extremely rare event [61].

The Ad_{GV} (HIV).11D vectors contain HIV-1 antigen open reading frame (ORF) expression cassettes inserted to replace the deleted adenovirus E1 gene region. Other deleted adenovirus regions include a partial E3 and all of E4, which has been replaced with a transcriptionally inert spacer element (T1S1) that enhances production of the adenoviral vectors [62].

The 293-ORF6 cell line used to propagate these E1, E4 and partial E3 deleted vectors was developed at GenVec, Inc. These cells were constructed by stably transforming 293 cells (which are of human embryonic kidney origin) with an inducible E4-ORF6 expression cassette. This enables the cells to efficiently complement the E1-, E4-, and partial E3-deleted adenoviral vectors, provide increased transgene capacity and greatly reduce the potential to generate replication-competent adenovirus. The particular clone that has given rise to the cell line is the A232 clone. All references to the 293-ORF6 cell line refer to cells derived from the original A232 clone. This replication-deficient adenoviral vector system has been used to produce TNFerade, a TNF-alpha gene-based product [63]. An assay for replication-competent adenovirus is performed in the final release testing for all vectors; RCA has not been observed in this packaging system during the manufacture of multiple gene-based products.

The four vaccine adenoviral vectors are generated by introducing a DNA plasmid consisting of the adenoviral genome into the 293-ORF6 cells. The adenoviral vector in the lysate from the transfected cells is serially passaged to expand the titer of adenoviral vector. The identity and integrity of the passages is verified by polymerase chain reaction (PCR) assay and expression of the HIV-1 gene is confirmed by Western Blot analysis. Purified adenovector is produced by infecting the 293-ORF6 cells with the adenoviral vector in the lysate; after the infection of the cells is complete, the material is collected and the vector is purified from the cells. The four vaccine adenoviral vectors are purified using a CsCl gradient centrifugation process. CsCl is removed by dialyzing the virus preparation against the final formulation buffer (VRC-DILUENT013-DIL-VP). Purified adenoviral vector serves as a vector bank for subsequent production of the four vaccine adenoviral vectors. This vector bank is tested for sterility,

mycoplasma and other adventitious agents prior to its being used for manufacturing of clinical supplies.

2.1.1 Production of the *gag-pol* Adenoviral Vector

AdtGagPol(B).11D

The protein sequences of the Gag and Pol proteins from an HIV-1 clade B were used to create a synthetic polyprotein version of the *gag-pol* genes using codons optimized for expression in human cells. The synthetic *gag* gene is from HIV-1 clade B strain HXB2 (GenBank accession number K03455), and the synthetic *pol* gene (*pol/h*) is from HIV-1 clade B NL4-3 (GenBank accession number M19921). The *pol* gene is nonfunctional because it is present as a fusion protein. Mutations were introduced in the synthetic protease and reverse transcriptase genes. The protease modification prevents processing of the *pol* gene product, and reduces the potential for functional protease, reverse transcriptase and integrase enzymatic activity. The cDNA used to produce AdtGagPol(B).11D is similar to an HIV-1 DNA vaccine VRC-4302 (BBIND 9782) which was tested and shown to have no reverse transcriptase activity. No modifications were made to the *gag*. To construct the adenoviral vector, the HIV-1 DNA sequence was subcloned using standard recombinant DNA techniques into an expression cassette in an E1-shuttle plasmid.

2.1.2 Production of the *env* Adenoviral Vectors

Adgp140(A).11D

The protein sequence of the envelope polyprotein (gp160) from 92rw020 (CCR5-tropic, GenBank accession number U08794) was used to create a synthetic version of the gene (clade-A gp140delCFI) using codons altered for expression in human cells. Plasmids expressing the HIV-1 genes were made synthetically with sequences designed to disrupt viral RNA structures that limit protein expression by using codons typically found in human cells. To construct the adenoviral vector, the HIV-1 DNA sequence was subcloned using standard recombinant DNA techniques into an expression cassette in an E1-shuttle plasmid.

Adtgp140dv12(B).11D

The protein sequence of the envelope polyprotein (gp160) from HXB2 (X4-tropic, GenBank accession number K03455) was used to create a synthetic version of the gene (X4gp160/h) using codons optimized for expression in human cells. To produce a CCR5-tropic version of the envelope protein (R5gp160/h), the region encoding HIV-1 envelope polyprotein amino acids 275 to 361 from X4gp160/h (VRC3300) was replaced with the corresponding region from the BaL strain of HIV-1 (Genbank accession number M68893, again using human preferred codons). The full-length CCR5-tropic version of the envelope protein gene from pR5gp160/h (VRC3000) was terminated after the codon for amino acid 680. The truncated Env glycoprotein (gp140) contains the entire surface protein and the ectodomain of gp41 including the fusion domain, and regions important for oligomer formation, specifically two helical coiled coil motifs. The Env V1 and V2 loops were deleted to improve the stability and yield of the vector in the producer cell line (G. Nabel, personal communication). Two additional amino acids were incorporated immediately after the deletion due to creation of a restriction enzyme site. In order to construct the adenoviral vector, the HIV-1 DNA sequence was subcloned using standard recombinant DNA techniques into an expression cassette in an E1-shuttle plasmid.

Adgp140(C).11D

The protein sequence of the envelope polyprotein (gp140delCFI) from 97ZA012 (CCR5-tropic, GenBank accession number AF286227) was used to create a synthetic version of the gene (clade-C gp140delCFI) using codons optimized for expression in human cells. To construct the adenoviral vector, the HIV-1 DNA sequence was subcloned using standard recombinant DNA techniques into an expression cassette in an E1-shuttle plasmid.

All Four Adenoviral Vectors

The four E1-shuttle plasmids were recombined in *Escherichia coli* (*E. coli)* BjDE3 bacteria with the GV11 adenovector-based AdFAST^{TM} plasmid pAdE1(BN)E3(10)E4(TIS1) to generate the adenoviral vector plasmids. The replication-deficient adenoviral vectors AdtGagPol(B).11D, Adgp140(A).11D, Adtgp140dv12(B).11D, and Adgp140(C).11D were then generated by introducing the adenoviral vector plasmid into the packaging cell line, 293-ORF6.

2.2 PREPARATION OF THE BULK PLASMID AND FINAL PRODUCTS

The investigational vaccine, VRC-HIVADV014-00-VP, is manufactured by GenVec, Inc. (Gaithersburg, MD) at a contract manufacturer, Molecular Medicine (San Diego, CA). DNA plasmids produced by the Vaccine Research Center, NIAID, NIH (Bethesda, MD) are used to construct the adenoviral vector clinical seed stock. The Phase I clinical production for each adenoviral vector will be performed by Molecular Medicine from clinical seed stock produced by Bioreliance (Rockville, MD).

The multiclade adenoviral vector vaccine product, VRC-HIVADV014-00-VP, is a 3:1:1:1 ratio of the adenoviral vectors that encode for HIV-1 Gag/Pol polyprotein from clade B and HIV-1 Env glycoproteins from clades A, B, and C, respectively. Final product meeting all test specifications will be released for use in the proposed clinical study. Vials will be filled to 1.2 mL volume with 1 x10^{10} PU/mL.

The final formulation buffer (FFB) is custom manufactured by BioWhittaker (Frederick, MD) and identified as VRC-DILUENT013-DIL-VP. The FFB used in the manufacture of the vaccine is composed of sodium chloride, Tris buffer, trehalose•$2H_2O$ (low endotoxin), magnesium chloride•$6H_2O$, monooleate (Tween 80) and water for injection (WFI).

2.3 RELEASE CRITERIA

GenVec, Inc. will perform release testing for the final clinical trial vaccine product. The safety testing performed prior to release of the manufactured lots of adenoviral vectors includes, but is not limited to, the verification of sterility and absence of contaminating organisms by testing for mycoplasma and endotoxin, determination of the level of replication-competent adenovirus (RCA), determination of host cell DNA and protein levels and genetic structural integrity.

Additional viral safety testing is performed on the Master Vector Banks and the final product. This includes, but is not limited to, tests for *in vivo* and *in vitro* inapparent adventitious virus, HIV-1, HIV-2, human T-cell lymphotropic virus (HTLV) I, HTLV II, CMV, hepatitis B, hepatitis C, Epstein-Barr virus, adeno-associated virus (AAV), parvovirus B19, human herpes virus (HHV)- 6, HHV-7, HHV-8, and fluorescent product enhanced reverse transcriptase (F-PERT, a test for retroviruses). In addition to safety testing, the level of HIV-1 gene expression is also quantified from manufactured lots of the four adenoviral vectors by transfection and

Western Blot analysis.

The lot release form notes the number of particle units (PU) in the final product. The clinical protocols specify the dose in PU. Particle units are the number of viral particles, active or not, found in the product as determined by spectrophotometry. Particle units, rather than plaque forming units (pfu) or fluorescent forming units (ffu), are used to determine dose because of the potential toxicity and host immune response to the viral particle, regardless of its ability to infect the target cells. It is also a more accurate measure than either pfu or ffu, which are highly dependent on methodology, and thus, more variable. Furthermore, the Food and Drug Administration (FDA) and the Recombinant DNA Advisory Committee have recommended the use of "PU."

2.4 PRECLINICAL SAFETY STUDIES

The Investigator Brochure for the adenoviral vector vaccine provides more extensive information about the preclinical safety studies. Briefly described here are the biodistribution and toxicology studies conducted in rabbits with the rAd, VRC-HIVADV014-00-VP and the toxicology study in rabbit of a prime-boost regimen using VRC-HIVDNA009-00-VP for the prime and VRC-HIVADV014-00-VP for the booster vaccination. Because the intention is to use a single injection of the rAd in the human clinical studies, a two injection series was used in the rabbit toxicology studies.

2.4.1 Biodistribution Study of VRC-HIVADV014-00-VP in Rabbits

Gene Logic Inc. conducted a single-dose biodistribution study of the rAd vaccine VRC-HIVADV014-00-VP in New Zealand White rabbits under Good Laboratory Practices (GLP) using intramuscular injections delivered by a needle and syringe. Animals were treated with 0.5 mL of control Final Formulation Buffer (FFB, VRC-DILUENT013-DIL-VP) or 0.95×10^{11} PU of VRC-HIVADV014-00-VP in 0.5 mL on study day (SD) 1. Five animals per sex per timepoint in the test article group and one animal per sex per time point in the control group were sacrificed on SD 9, SD 61 and SD 91. All tissues were shipped to Althea Technologies, Inc. (San Diego, CA) and processed for the presence of the adenoviral vector in the tissues using a GLP validated Taqman™ Polymerase Chain Reaction (PCR), developed and qualified to detect a specific target sequence in each of the four different adenoviral vectors comprising VRC-HIVADV014-00-VP. The assay detects an amplicon from each of the adenoviral vectors. The 5’- PCR primers, 3’-PCR primers and fluorescently labeled probes span regions containing the insert, polylinker and promoter. The lower limit of detection for this assay is 10 copies of the target/µg of DNA, the lower limit of quantification for the assay is 50 copies of the target/µg of DNA.

Tissues analyzed included: blood, gonads, heart, lung, liver, kidney, lymph nodes, spleen, thymus, subcutis and thigh muscle (at injection site), bone marrow (from femur on side of injection) and brain. No treatment-related changes in mortality, clinical signs of toxicity, body weights or body weight changes were observed. Food consumption in the male group receiving the test article was decreased during the 24-hour period following the injection, but returned to normal after that period. The distribution profile (detailed in the table below) consisted of the VRC-HIVADV014-00-VP test article present at the injection site subcutis (5/10 animals, SD 9; 2/10 animals, SD 61) and muscle (4/10 animals, SD 9), and in the spleen (10/10 animals, SD 9; 6/10 animals, SD 61; 5/10 animals, SD 91) and liver (9/10 animals, SD 9; 2/10 animals, SD 61),

with sporadic findings in the bone marrow (1/10 animals, SD 9, counts below 200 copies/µg of DNA). The number of copies of the VRC-HIVADV014-00-VP test article decreased considerably from SD 9 to SD 61 in all tissues with positive findings, and between SD 61 and SD 91 for the liver and injection site.

Summary of Number of Rabbits with Positive Findings in Five Tissue Types and Average Number of Copies of Target/µg DNA

	Marrow	Liver	Spleen	Subcutis	Muscle
**Day 9**					
Number with positive reactions	1/10	9/10	10/10	5/10	4/10
Average number of copies	23	945	1934	8088	2751
**Day 61**					
Number with positive reactions	0/10	2/10	6/10	2/10	0/10
Average number of copies	N/A	118	113	232	N/A
**Day 91**					
Number with positive reactions	0/10	0/10	5/10	0/10	0/10
Average number of copies	N/A	N/A	124	N/A	N/A

2.4.2 Toxicology Study of VRC-HIVADV014-00-VP Alone and in a Prime-Boost Regimen with the DNA Vaccine, VRC-HIVDNA009-00-VP

Gene Logic, Inc. (Gaithersburg, MD) conducted the GLP toxicology studies. The objective was to assess the potential toxicity of VRC-HIVADV014-00-VP when administered alone or as a boost following a priming regimen with the DNA vaccine VRC-HIVDNA009-00-VP in New Zealand White rabbits.

For the VRC-HIVADV014-00-VP alone study, ten animals/sex/group received 1 mL of Final Formulation Buffer (FFB, VRC-DILUENT013-DIL-VP) or $1x10^{11}$ PU of VRC-HIVADV014-00-VP on SD 1 and SD 22. On SD 1, two 0.5 mL injections were administered into two sites in the right thigh muscle at injection site 1 and 2. On SD 2, two injections were administered into the left thigh muscle at injection site 3 and 4. Five animals per sex per group per timepoint were necropsied on SD 24 and SD 36.

For the VRC-HIVDNA009-00-VP DNA prime/VRC-HIVADV014-00-VP rAd boost study, ten animals/gender were dosed via intramuscular injection. DNA vaccine (4 mg per time point) and PBS control were administered four times (SD 1, 22, 43, and 64). The dosage was divided into 2 intramuscular injections spaced approximately 1 inch apart (0.5 mL/injection site). The dose volume for each injection was not adjusted for body weight. Injections were administered into the thigh muscle using a Biojector 2000 needle-free injection management system (Biojector 2000) and on alternate sides for each time point. The adenoviral vector vaccine, VRC-HIVADV014-00-VP, (10^{11} PU per time point) and the diluent control (VRC-DILUENT013-DIL-VP) injections were administered as two 0.5 mL injections into the thigh muscle approximately 1 inch apart per day of dosing (SD 85 and 106) into the hind thigh muscle with a needle and syringe. Injections were administered on alternate sides for each time point. 1.0 mL was administered regardless of body weight for DNA and adenovector doses and their respective

controls. One half of the animals (5/gender) were sacrificed on study day 108 and the remainder on study day 120. Injections were administered at a shaved/marked site. The sites were re-shaved and re-marked as needed in order to visualize the injection site.

Parameters evaluated included mortality, clinical signs of toxicity, Draize observations, body weights, body weight changes, food consumption, ophthalmologic examinations, clinical pathology (chemistry, hematology and coagulation), body temperatures, gross pathology, organ weights, and histopathology. Observations were also made of motor function and behavior during the study. Tissues analyzed included: adrenals, aorta, brain, cecum, colon, cervix, duodenum, epididymides, esophagus, eyes, femur, gall bladder, gross lesions, harderian glands, heart, ileum, injection sites, jejunum, kidneys, liver, lung, lymph nodes, mandibular salivary glands, mammary glands, optic nerves, ovaries, pancreas, pituitary, prostate, rectum, seminal vesicles, skeletal muscle, skin, spinal cord, spleen, sternum with marrow, stomach, sciatic nerve, testes, thymus, thyroid/parathyroid, trachea, tongue, urinary bladder, uterus, vagina.

All animals survived to sacrifice and necropsy. No adenovector alone treatment-related observations were made with regard to morbidity/clinical observations, body weights and changes, and ophthalmology. No prime-boost treatment-related observations were made with regard to morbidity/clinical observations and ophthalmology. However, possible prime-boost treatment effects were seen with body weights and changes, particularly in treated females. Differences began to be noted as early as study day 36, but became statistically significantly different from control females on study days 71, 78, 92, 99, and 108 for body weights and days 85-92 for body weight changes in prime-boost treated females. These animals continued to gain weight over the course of the study, but did not gain as much weight as the controls.

Minimal erythema was seen at the injection sites in a few treated males and one control female after the second adenovector injection when given alone. In contrast, in the prime-boost regimen, vaccination with the DNA prime resulted in Draize observations of minimal to moderate edema and erythema increasing in frequency and severity with repeated dosing. This was likely a result of the combination of injection with Biojector 2000 and the active vaccination, as these observations also occurred in the control animals but to a lower amount and lesser degree. These findings were consistent with previous toxicology studies performed with DNA vaccination alone. Boost (adenovector delivered by needle and syringe) injections did not increase the frequency or severity (minimal erythema and/or edema in a few treated animals) of the Draize observations seen at earlier timepoints (after priming doses).

Clear treatment-related (adenovector alone and prime-boost) observations were histopathological findings of inflammation in the perineural tissue surrounding the sciatic nerve (near the injection site). These latter lesions consisted of chronic inflammatory cells (small macrophages and lymphocytes) in the connective tissue around the sciatic nerve and in adjacent lymphatics and blood capillaries. This inflammation was likely the result of drainage of the distal injection sites toward proximal lymph nodes. The injection site reactions decreased in frequency and severity in the recovery sacrifice animals compared to the immediate sacrifice animals for both the adenovector alone and the prime-boost regimens, demonstrating the reversibility of the injection site reactions.

Fever was observed in the 24 hours subsequent to the initial, but not second, adenovector vaccination (adenovector only arm) – more striking in treated males than females. These resolved by 48 hours. Likewise, fever was seen in treated males and females in the 24 hours

subsequent to the initial treatment, but only in the first 3 hours after the second adenovector boost (only in treated females), in the prime-boost treated animals. These fevers resolved by 48 hours after the initial treatment and 24 hours after the second (treated females only) adenovector boost.

Food consumption was also decreased compared to controls in the 24 hours (adenovector alone and prime-boost) to 48 hours (prime-boost) following each adenovector vaccination, but resolved, and did not result in differences in body weights or changes in males or females inoculated with adenovector alone or treated males in the prime-boost regimen. Significant differences in body weight changes in treated females were noted in treated females in the prime-boost regimen (although these differences began prior to exposure to adenovector as discussed above).

There were additional observations, particularly in clinical chemistries and hematology parameters, which were unclear in their relationship to treatment because they either remained within the historical normal range for the species, even though there were statistically significant differences from matched control animals on study, or they were outside of the normal range and different from the control animals on study, but were not consistent between genders or across timepoints. None of these findings appeared to correlate with clinical observations or gross or histopathological findings. Of note among these, however, was the finding of statistically significant (from matched controls on study) elevated triglycerides on the second day subsequent to the initial adenovector inoculation in both treated males [mean was 1.5 times the upper limit of normal (ULN)] and females (mean was >1 but <1.5 times the ULN) receiving adenovector vaccination alone and on the day subsequent to the initial adenovector boost in treated males (mean was between 3-4 times the ULN), but not treated females, receiving the prime-boost regimen. There were no differences between groups in this parameter after the second inoculation with the adenovector, either when given alone or as a boost, in either gender. At day 86, several differences (statistical and not within normal historical range) were noted in Group 4 hematological values, including lower platelets and neutrophils (females), lower lymphocytes (males) and higher neutrophils (males). Lack of corresponding data in the opposite sex and over multiple timepoints makes any correlation to the vaccine unclear.

2.5 PRECLINICAL IMMUNOGENICITY STUDIES

The VRC-HIVADV014-00-VP Investigator's Brochure provides more extensive information about the preclinical immunogenicity studies. There is no adequate animal model of HIV-1 infection and, as a consequence, there can be no animal studies in which to test pharmacology and protective immunization with the prime-boost vaccine regimen. Non-clinical immunogenicity studies were conducted in mice and non-human primates by investigators at the Vaccine Research Center (VRC), National Institutes of Allergy and Infectious Diseases, National Institutes of Health (Bethesda, MD) and Beth Israel Deaconess Medical Center, Harvard Medical School (Boston, MA). In addition, humoral immune responses were assessed in rabbits as part of the GLP preclinical toxicity studies (Gene Logic Study # 1195-114). The immunogenicity studies in mice, rabbits and non-human primates are summarized below. The vaccines used were formulated exactly as the clinical trial material.

Several assays were used to evaluate immune responses elicited by the vaccine. Cellular immune responses were tested by the interferon gamma (IFN-γ) ELISPOT assay and the flow cytometry-based intracellular cytokine staining (ICS) assay. The IFN-γ ELISPOT quantitatively

measures the production of IFN-γ by peripheral blood mononuclear cells (PBMC) from immunized animals. The cells are exposed *in vitro* to HIV-1 antigens (a series of short, overlapping peptides that span the length of the protein expressed in the vaccine). IFN-γ produced by antigen-sensitized T-lymphocytes is bound to an antibody-coated assay plate and may be counted colormetrically as spot forming cells (SFC) by using an alkaline phosphatase conjugated read-out system. The results are expressed as SFCs per million PBMC. Similarly, the ICS assay uses a flow cytometry based system to measure IFN-γ (and sometimes additional cytokines) produced by antigen-stimulated cells. In this system, the stimulated cells are further characterized by phenotypic lymphocyte markers, allowing for precise quantification of the type of cells (for example $CD4^+$ or $CD8^+$ T-lymphocytes) responding to the vaccine antigens.

Humoral immune responses were measured using ELISA assays or a modified assay where the vaccine proteins were bound to the test plate using a lectin-capture system.

2.5.1 Immune Responses to VRC-HIVADV014-00-VP Alone in Mice

A non-GLP study, "Immune responses to vaccination with VRC-HIVADV014-00-VP in mice" was conducted at the Vaccine Research Center, National Institutes of Health (Bethesda, MD). This study was designed to examine humoral and cellular immune responses. Two groups of mice were vaccinated once by intramuscular injection. In each group five mice received empty plasmid (control) injections and ten mice received an injection of 10^{11} PU of test article (VRC-HIVADV014-00-VP). Immune responses were tested 10 days after the injection.

HIV-1-specific cellular immune responses in vaccinated mice were demonstrated by intracellular flow cytometry. Assuming that a frequency of >0.1% cytokine producing cells represents a positive result, $CD4^+$ responses were observed in 3/10 (Gag), 7/10 (Pol), 8/10 (Env-A), 10/10 (Env-B) and 9/10 (Env-C) mice. $CD8^+$ responses were observed in 9/10 (Gag), 10/10 (Pol), 6/10 (Env-A), 6/10 (Env-B) and 7/10 (Env-C) mice. All mice also had demonstrable antibody titers (measured by ELISA) to HIV-1 proteins, following immunization with VRC-HIVADV014-00-VP.

2.5.2 Immune Responses to VRC-HIVDNA009-00-VP Prime Followed by VRC-HIVADV014-00-VP Boost in Rabbits

This immunogenicity study was performed at the Vaccine Research Center, National Institutes of Health (Bethesda, MD) as part of the GLP Repeated-Dose Toxicology study of VRC-HIVADV014-00-VP.

All serum samples from rabbits in Group 1 and prebleeds for Group 2 (rAd alone article vaccinated) had low raw optical densities (OD), with the average OD ± standard deviation = 0.159 ± 0.105 (n= 480) at dilutions of 1:100 and 1:1000. All rabbits in Group 2 for the day 24 samples had evidence of seroconversion at serum dilutions of 1:1000, with raw optical densities for all antigens > 0.21 with the average OD ± standard deviation = 2.71 ± 1.07 (n= 160). All rabbits in the vaccinated group (Group 2) had detectable antibody concentrations for HIV Env-A, Env-B, Env-C and Gag. Therefore, this study demonstrated vaccine-induced immunogenicity to all components of the vaccine formulation.

All samples from rabbits in Group 3 (Control) and prebleeds for Group 4 (DNA prime + rAd boost) had low raw OD, with the average prevaccination OD ± standard deviation = 0.099 ±

0.065 (Group 3, n=160 samples) and 0.129 ± 0.138 (Group 4, n = 160 samples). Further, there were very high OD values for all antigens post vaccination for rabbits in Group 4. While there are a few rabbits in Group 4 with higher OD values in the pre-vaccination sample, they did have elevated OD values at day 108 (OD = 3.529 ± 0.812), indicative of induced immune responses.

2.5.3 Immune Responses to VRC-HIVADV014-00-VP Alone in Cynomolgus Macaques (VRC 03-060)

A non-GLP study in Cynomolgus macaques was conducted at Beth Israel Deaconess Medical Center, Harvard Medical School (Boston, MA) and the Vaccine Research Center (Bethesda, MD). This study was designed to investigate the magnitude and breadth of cellular immune responses in rhesus monkeys that were elicited by the recombinant adenovirus (rAd) immunizations. The study included six outbred Cynomolgus macaques. Immunizations with VRC-HIVADV014-00-VP were administered at 10^{11} PU (delivered as two 0.5 mL injections in the quadriceps muscles) IM by needle and syringe. The macaques were bled Week 2 and Week 4 post-immunization.

ELISPOT assays were utilized to monitor the emergence of vaccine-elicited T cell immune responses to multiple viral antigens. All six macaques generated responses to clades A, B and C Env peptide pools. The study data demonstrated that the clinical rAd product is immunogenic and induces cellular immune responses against clades A, B, C Env, as well as against Gag and Pol.

2.5.4 Immune Responses to VRC-HIVDNA009-00-VP Prime Followed by VRC-HIVADV014-00-VP Boost in Cynomolgus Macaques (VRC-02-035)

Non-GLP studies were conducted at Beth Israel Deaconess Medical Center, Harvard Medical School (Boston, MA) and the Vaccine Research Center (Bethesda, MD) to investigate the magnitude and breadth of cellular and humoral immune responses in Cynomolgus macaques that were elicited by different DNA prime/rAd boost immunization regimens using the DNA plasmid and rAd vaccines intended for use in human trials. GLP-grade DNA plasmid expressing clade B Gag-Pol-Nef fusion protein [produced by Althea Technologies, Inc. (San Diego, CA)] and the multiclade A, B and C HIV-1 Env plasmids contained in VRC-HIVDNA009-00-VP (BB-IND 10681) were used for the prime immunization. GMP-grade VRC-HIVADV014-00-VP was used as the rAd boost. Groups of six outbred adult Cynomolgus macaque monkeys were immunized in this study. This summary describes results for the group that was immunized with 8 mg of the DNA vaccine delivered IM at weeks 0, 4, and 8 by Biojector, and 10^{11} PU of the rAd vaccine construct delivered IM by needle and syringe at week 38. In each case, plasmid vaccine was delivered as two 0.5 mL injections in the quadriceps muscles using a No. 3 Biojector syringe. Cynomolgus macaques receiving DNA prime/rAd boost immunizations elicited potent and broad cellular immune responses simultaneously to all viral antigens. In addition, they elicited potent antibody responses to these envelope antigens and Gag antigen as measured by ELISA. Monkeys that received the DNA plasmid vaccine prime and rAd boost generated responses to clades A, B and C Env peptide pools in all six animals following the DNA prime immunizations, as well as following the rAd boost.

Five of six animals developed antibody responses to all three envelope antigens. One animal (# 629) developed antibody responses only to clades A and C envelope antigens. All six monkeys had strong Env antibody responses after adenovirus boost.

These data demonstrate that the clinical rAd product is immunogenic, when given with a DNA prime, and induces cellular immune responses against clades A, B, and C Env, as well as Gag and Pol, and antibody responses against clades A, B, and C Env, as well as Gag. Adenoviral vector boosting increases the immune responses several fold.

3. STUDY OBJECTIVES

3.1 PRIMARY OBJECTIVES

- To evaluate the safety and tolerability of VRC-HIVADV014-00-VP when administered intramuscularly at 1 x 10^{10} PU by needle and syringe to uninfected adult subjects who previously received three injections of the HIV multiclade DNA vaccine, VRC-HIVDNA016-00-VP, at a dose of 4 mg.

3.2 SECONDARY OBJECTIVES

- To evaluate the immunogenicity of VRC-HIVADV014-00-VP at Week 0, Week 2 (for cellular responses only), Week 4 and Week 6 when administered intramuscularly at 1 x 10^{10} PU by needle and syringe to subjects who previously received three injections of the HIV multiclade DNA vaccine, VRC-HIVDNA016-00-VP, at a dose of 4 mg. HIV-1 Gag-Pol and Env-specific immune responses will be assessed by cellular immune function assays (intracellular cytokine analysis and ELISPOT), as well as by measures of humoral immunity (vaccine antigen-specific ELISA and neutralization assays).

- To evaluate adenovirus serotype 5 neutralizing antibody titers at Week 0 and Week 4.

- To monitor the social impact of participating in an HIV-1 vaccine clinical trial using a questionnaire completed at Week 24.

3.3 EXPLORATORY ANALYSES

- To evaluate the immunogenicity of VRC-HIVADV014-00-VP at Week 12 and Week 24 when administered intramuscularly at a dose of 1 x10^{10} PU by needle and syringe to subjects who previously received three injections of the HIV multiclade DNA vaccine VRC-HIVDNA016-00-VP at a dose of 4 mg. The HIV-1 Gag-Pol and Env-specific immune responses will be assessed by cellular immune function assays (intracellular cytokine analysis and ELISPOT) at Week 12 and Week 24, as well as by measures of humoral immunity (ELISA and neutralization assays) at Week 24.

- To evaluate adenovirus serotype 5 neutralizing antibody titer at Week 24.

- To perform epitope mapping of the $CD8^+$ and $CD4^+$ T cell responses using peptide pool matrices, as well as detailed evaluation of the phenotype of the responding cells collected at Week 4. The patterns of expression for cytokines, chemokines, surface proteins, and markers of degranulation in individual cells will be determined using multi-color flow cytometric analysis.

4. STUDY DESIGN AND METHODS

This is a Phase I study to examine safety and tolerability of and immune response to a multiclade

HIV adenoviral vector vaccine, VRC-HIVADV014-00-VP, when administered as a booster vaccine to healthy adults previously vaccinated with the multiclade DNA vaccine VRC-HIVDNA016-00-VP. It is hypothesized that this vaccine will be safe as a booster vaccine and elicit immune responses to HIV. The National Institutes of Health will conduct the clinical trial. The study will be initiated after all regulatory requirements are met.

Subjects who completed 3 injections of 4 mg of vaccine in VRC 007 (04-I-0254) and indicated willingness to participate in a booster vaccine study will be invited to screen for this study. Subjects who meet eligibility criteria and enroll will be vaccinated with VRC-HIVADV014-00-VP at 1 x 10^{10} PU IM administered in a deltoid muscle using a needle and syringe. Adverse reactions will be evaluated at scheduled study visits and by study subject report. Specimens to evaluate immunogenicity will be taken at baseline and at specified timepoints (see Appendix III). The PBMC samples collected at Week 4 (Day 28±3 days) will be obtained by an apheresis procedure, if the subject is able and willing to undergo apheresis and if the procedure can be scheduled within the visit window; otherwise a routine blood draw will be done to obtain a smaller number of PBMC for research studies. The Department of Transfusion Medicine at the NIH Clinical Center will perform the apheresis procedure.

4.1 STUDY POPULATION

To be eligible for VRC 010, a potential study participant must have received three injections at the 4 mg dosage of the investigational vaccine VRC-HIVDNA016-00-VP in VRC 007 (04-I-0254). Fourteen of the 15 subjects who enrolled in VRC 007 completed three injections. Therefore, there are 14 people who may be eligible to participate in this study. The VRC 007 study participants ranged from 20 to 36 years of age at the time of VRC 007 enrollment and these enrollments occurred between 8/17/04 and 10/28/04. Subjects were comprised of 67% males and 37% females; with 11 (73.3%) identified as white, not Hispanic/Latino and 4 (26.6%) identified as Hispanic/Latino or multiracial. The VRC 007 consent noted that subjects may be invited to participate in a booster study. Subjects from VRC 007 who, by study participation history, appear to meet VRC 010 eligibility requirements will be notified when recruitment is ready to begin. It is estimated that for this subject population, the interval between the first priming DNA vaccination in VRC 007 and the boosting adenoviral vector vaccination in VRC 010 will be kept as close as possible to 24 weeks but may range up to 36 weeks if needed to accommodate study subject schedules or a delay in the initiation of the study.

Prior to signing the VRC 010 informed consent form, eligible volunteers will take a short "Assessment of Understanding" quiz to test their understanding of this vaccine study. Incorrect answers will be explained to the volunteer and they will sign the informed consent document only after the study coordinator is satisfied with their understanding of the study.

4.1.1 Inclusion Criteria

A participant must meet all of the following criteria:

1. Enrolled into VRC 007 no more than 36 weeks prior to VRC 010 enrollment and completed three injections of 4 mg of study vaccine in VRC 007 (04-I-0254) without experiencing a serious adverse event (SAE) that was possibly, probably or definitely related to study vaccine.

2. Available for clinical follow-up for 24 weeks after enrollment into VRC 010.

3. Completion of an Assessment of Understanding prior to enrollment and able to verbalize understanding of all questions answered incorrectly.

4. Able and willing to complete the informed consent process.

5. Willing to receive HIV test results and willing to abide by NIH guidelines for partner notification of positive HIV results.

6. Willing to donate blood for sample storage to be used for future research.

7. Willing to discuss HIV infection risks and amenable to risk reduction counseling.

8. In good general health without clinically significant medical history and satisfactory completion of the screening process.

***Laboratory Criteria within 28 days prior to enrollment**:*

9. Hemoglobin ≥ 11.5 g/dL for women; ≥13.5 g/dL for men

10. WBC = 3,300-12,000 cells/mm^3
11. Differential either within institutional normal range or accompanied by site physician approval

12. Total lymphocyte count ≥ 800 cells/mm^3
13. Platelets = 125,000 – 550,000/mm^3
14. ALT (SGPT) ≤ 1.25 x upper limit of normal

15. Serum creatinine ≤ 1 x upper limit of normal (≤ 1.3 mg/dL for females; ≤1.4 mg/dL for males)

16. Normal urinalysis defined as negative glucose, negative or trace protein, and no clinically significant blood in the urine

17. Negative HIV PCR (i.e., less than the lower limit of detection or <50 RNA copies/mL)

18. Negative Hepatitis B surface antigen

19. Negative anti-HCV (hepatitis C virus antibody) and negative HCV PCR

***Female-Specific Criteria**:*

20. Negative β-HCG pregnancy test (urine) on day of study enrollment for women presumed to be of reproductive potential.

21. A female participant must meet one of the following criteria:

    No reproductive potential because of menopause [one year without menses] or because of a hysterectomy, bilateral oophorectomy, or tubal ligation,

    or

    Participant agrees to be heterosexually inactive at least 21 days prior to enrollment and through Week 24 of the study,

    or

    Participant agrees to consistently practice contraception at least 21 days prior to enrollment and through Week 24 of the study by one of the following methods:
    - condoms, male or female, with or without a spermicide
    - diaphragm or cervical cap with spermicide
    - intrauterine device
    - contraceptive pills or patch, Norplant, Depo-Provera or other FDA-approved contraceptive method
    - male partner has previously undergone a vasectomy for which there is documentation.

4.1.2 Exclusion Criteria

A volunteer will be excluded if one or more of the following conditions apply:

Women:

1. Breast-feeding or planning to become pregnant during the 24 weeks of study participation.

Volunteer has received any of the following substances:

2. Immunosuppressive or cytotoxic medications or inhaled corticosteroids within the past six months (with the exception of corticosteroid nasal spray for allergic rhinitis or topical corticosteroids for an acute uncomplicated dermatitis).
3. Blood products within 120 days prior to HIV screening.
4. Immunoglobulin within 60 days prior to HIV screening.
5. Live attenuated vaccines within 30 days prior to initial study vaccine administration.
6. Investigational research agents within 30 days prior to study vaccine administration.
7. Medically indicated subunit or killed vaccines, e.g. influenza, pneumococcal, or allergy treatment with antigen injections, within 14 days of study vaccine administration.
8. Current anti-TB prophylaxis or therapy.

Volunteer has a history of any of the following clinically significant conditions:

9. Serious adverse reactions to vaccines such as anaphylaxis, hives, respiratory difficulty, angioedema, or abdominal pain.

10. Autoimmune disease or immunodeficiency.

11. Asthma that is unstable or required emergent care, urgent care, hospitalization or intubation during the past two years or that requires the use of oral or intravenous corticosteroids.

12. Diabetes mellitus (type I or II), with the exception of gestational diabetes.

13. History of thyroidectomy or thyroid disease that required medication within the past 12 months.

14. Serious angioedema episodes within the previous 3 years or requiring medication in the previous two years.

15. Hypertension that is not well-controlled by medication or is more than 145/95 at enrollment.

16. Bleeding disorder diagnosed by a doctor (e.g. factor deficiency, coagulopathy, or platelet disorder requiring special precautions) or significant bruising or bleeding difficulties with IM injections or blood draws.

17. Syphilis infection that is active or a positive serology due to a syphilis infection treated less than six months ago.

18. Malignancy that is active or treated malignancy for which there is not *reasonable* assurance of sustained cure or malignancy that is likely to recur during the period of the study.

19. Seizure disorder other than: 1) febrile seizures under the age of two, 2) seizures secondary to alcohol withdrawal more than 3 years ago, or 3) a singular seizure not requiring treatment within the last 3 years.

20. Asplenia or any condition resulting in the absence or removal of the spleen.

21. Psychiatric condition that precludes compliance with the protocol; past or present psychoses; past or present bipolar disorder; disorder requiring lithium; or within five years prior to enrollment, history of a suicide plan or attempt.

22. Any medical, psychiatric, social condition, occupational reason or other responsibility that, in the judgment of the investigator, is a contraindication to protocol participation or impairs a volunteer’s ability to give informed consent.

4.2 SUBJECT MONITORING: SCHEDULE OF EVALUATIONS

Evaluation of the safety of the booster vaccination will include laboratory studies, physical assessment by clinicians, and subject self-assessment recorded on a diary card for 5 days after

the injection. Blood tests for cellular and humoral immune responses will be performed. The schedule for study evaluation is described in this section and shown in table format in Appendix III. Total blood volume drawn from each subject will not exceed the NIH Clinical Center Guidelines of 450 mL in any 6-week period.

4.2.1 Screening

VRC 007 study participants who are potentially eligible for VRC 010 and who indicate willingness to consider a booster vaccine study will be screened for this study through the screening protocol VRC 000 (02-I-0127). Enrollment into VRC 010 is targeted to occur at VRC 007 study Week 24 whenever possible, but may occur as late as 36 weeks after the date of VRC 007 enrollment. Test results obtained at regularly scheduled VRC 007 study visits may be used for VRC 010 eligibility screening or a separate screening visit may be scheduled.

VRC 007 subjects who roll over into VRC 010 at the interval in VRC 007 from Week 24 (visit 06) through Week 32 (visit 07 and last VRC 007 visit) will have the data normally collected for VRC 007 entered into the VRC 007 study database, as well as the VRC 010 study database. Any visit from Week 24 through Week 32 in which a VRC 007 subject rolls over in VRC 010, will be noted in the VRC 007 database as the last study visit for VRC 007. At the point of enrollment into VRC 010, the subject will be noted to be “off-study” for VRC 007 and “on-study” for VRC 010. If the subject does not enroll into VRC 010 exactly at what would be the Week 24 visit for 007, then the VRC 007 Week 24 blood draw must be considered by the Study Coordinator in selecting a date for rollover enrollment into VRC 010 so as not to exceed the total of 450 mL in the first 6 weeks of VRC 010 study participation.

For reference in planning this study it is noted that the projected target dates for VRC 007 visits 06 (Week 24) range from 2/1/05 through 4/14/05 and the target dates for VRC 007 last visits (Week 32) range from 3/29/05 through 6/9/05. The eligibility window for rolling over into VRC 010 is 36 weeks from date of VRC 007 enrollment. Therefore, the last eligibility date for VRC 010 enrollments will range from 4/26/05 (for subject 007001) through 7/7/05 (for subject 007015).

Evaluations required to establish eligibility for VRC 010 include the following:

- Medical history, including questions regarding sexual behavior and other practices that relate to HIV risk
- Physical examination
- Hematology: complete blood count (CBC) including differential and platelets; also $CD4^+$ and $CD8^+$ cell counts
- Chemistry: creatinine, alanine aminotransferase (ALT)
- Urinalysis
- Infectious diseases screening: rapid plasma reagin (RPR), hepatitis B surface antigen, anti-HCV, HCV PCR, HIV ELISA (Western Blot, if indicated), HIV PCR
- Pregnancy test (for females of reproductive potential)

It should be noted that VRC 007 includes HIV screening tests and some of the other eligibility tests listed. At screening for VRC 010, potential participants may have vaccine-induced positive ELISA and indeterminate or positive Western blots from the priming vaccinations with the DNA vaccine in VRC 007. Eligibility will depend on having a negative HIV PCR result at screening.

The informed consent document for VRC 010 will be reviewed and counseling related to HIV prevention and the potential risks of becoming pregnant during study participation will be provided. Multiple screening visits may be needed to complete the eligibility screening and pre-enrollment counseling. As the final step in the screening process, an Assessment of Understanding of VRC 010 is completed on the day the subject is scheduled to enroll in VRC 010, but prior to enrollment.

4.2.2 Day 0 Through Week 24 Follow-Up

The evaluations and windows for scheduling visits are outlined below and shown in the Appendix III Schedule of Evaluations.

In VRC 010, Day 0 is defined as the day of study enrollment and study vaccination. Study eligibility is reviewed on Day 0 as part of the enrollment process. A negative pregnancy test result for a woman of reproductive potential must be obtained on the enrollment day prior to completing the enrollment and study injection. Following the study injection, subjects will be observed for a minimum of 30 minutes. Vital signs (temperature, blood pressure, pulse and respiratory rate) will be completed between 30 and 45 minutes post-injection. The injection site will be inspected for evidence of local reaction. In keeping with the NIH Clinical Center policy and good medical practice, acute medical care will be provided to subjects for any immediate allergic reactions or other injury resulting from participation in this research study. Day 0 evaluations obtained prior to the study injection are the baseline for subsequent safety assessments.

Subjects will be given a Diary Card on which to record temperature and symptoms daily for 5 days. Follow-up on subject well-being will be performed by telephone on the first or second day following injection. Events reported in the telephone interview that will require a clinic visit include rash, urticaria, fever of 38.7˚C (Grade 2) or higher, or significant impairment in the activities of daily living (ADL). At the Week 2 visit (14 days± 3 days after injection) study subjects will return for a clinic visit and laboratory tests and completed diary cards will be reviewed with a clinician.

Study subjects will have samples collected for immunologic assays on Day 0 and at Week 2 ± 3 days, Week 4± 3 days, Week 6± 3 days, Week 12± 14 days and Week 24± 14 days.

On Week 4± 3days the PBMC for immunological analysis will be collected by apheresis, unless the subject is unwilling, ineligible by DTM standards, or is unable to get a suitable appointment. If apheresis is not performed, PBMC will be obtained from 80 mL of blood collected by standard phlebotomy. Any cells, serum or plasma not used will be stored for future virological and immunological assays. HLA type was recorded for all VRC 007 subjects and may be used in the immunological analysis of VRC 010 also, but will not be repeated. Subjects will be asked to consent to include their HLA type in the VRC 010 database.

Study visit procedures and tests from Day 0 through Week 24 are as follows:

- VRC 010 "Assessment of Understanding" Quiz (Day 0)
- Signature of study participation informed consent form for VRC 010 (Day 0)
- Clinical evaluations: axillary lymph node exam, vital signs and weight (every visit); targeted physical exam on any visit if indicated by interim complaints or laboratory findings.

- Interim medical history (every visit).
- Counseling on HIV and avoidance of pregnancy (Day 0; offered every subsequent visit)
- Study injection (Day 0)
- Post-injection vital signs and assessment of injection site completed 30 to 45 minutes post-injection.
- Diary Card: Baseline on Day 0 prior to injection and 5-day diary card for self- assessment of symptoms to be completed following the injection. The diary card will include the parameters: unusually tired/feeling unwell, muscle aches (at other than injection site), headache, chills, nausea, pain/ tenderness at injection site, highest measured temperature and measurement of perpendicular diameters for redness and swelling at injection site. Completed diary cards are collected at the Week 2 visit.
- Pregnancy test for females of reproductive potential (Day 0, Week 24); obtain results prior to injection on Day 0; counseling on avoidance of pregnancy Day 0 and as needed throughout study participation.
- CBC, differential, platelet count (Day 0 and Weeks 2, 6, 12 and 24)
- Blood chemistry (creatinine and ALT) (Day 0 and Weeks 2, 6, 12 and 24)
- Urinalysis (Day 0 and Weeks 2, 6, 12, and 24)
- T lymphocyte subsets (CD4/CD8) (Day 0 and Week 24)
- HIV testing: ELISA/Western blot (Day 0, Week 6 and Week 24)
- HIV-1 RNA PCR (Day 0, Week 6 and Week 24)
- Vaccine antigen-specific ELISA and neutralizing antibody titers for specific vaccine responses (Day 0 and Weeks 4 and 24). These assays are not performed immediately, but rather are batch run from frozen samples at intervals.
- Intracellular cytokine analysis and ELISPOT (Day 0 and Weeks 2, 4, 6, 12 and 24). Note: The assays will not be performed immediately, but rather completed using frozen samples at a later date. PBMC and plasma for storage will be saved from the blood collected for these assays. Whenever possible, the Week 4 PBMC will be collected by apheresis and frozen plasma sample obtained from a tube collected prior to starting the apheresis. Week 4 PBMC will also be used for epitope mapping and other exploratory immunological studies. If apheresis is not performed at Week 4 then blood samples will be collected by phlebotomy. To be eligible for apheresis at Week 4, the subject must weigh at least 110 pounds, the hemoglobin value at Week 2 must be ≥12.5 g/dL, and the subject must meet DTM criteria for a research apheresis procedure on the day of apheresis.
- Serum for archiving (Day 0 and Weeks 2, 4, 6, 12 and 24); some of the Day 0, Week 4 and Week 24 serum will also be used for adenovirus serology.
- Social Impact Assessment (Week 24)

4.3 Monitoring for HIV Infection

It is possible that this vaccine will induce immunologic responses that are detected by standard HIV screening techniques, even though the vaccine itself will not cause HIV infection. Some subjects enrolling in VRC 010 may already have vaccine-induced HIV antibody from prior participation in VRC 007. The following steps will be taken to ensure detection of HIV infection and to protect participants from adverse consequences associated with an HIV antibody test that indicates an antibody response to the vaccine.

- Study participants will receive regularly scheduled counseling regarding avoidance of HIV infection according to the most recent CDC HIV Counseling Guidelines.

- Study participants will be screened for HIV infection periodically while participating in the study (see Appendix III for schedule of testing).

- If there is any clinical or laboratory indication of HIV infection, any test required to make a definitive diagnosis, including Western blot analysis, viral load measurement (PCR), or other tests will be performed.

- Confirming tests will be performed as soon as possible once a positive antibody response is identified. Participants will be promptly informed if they are HIV-infected. Participants who are found to have vaccine-induced antibody responses, but with no evidence of HIV infection, will be informed that they are not HIV-infected. Written documentation describing any vaccine-induced antibody response and confirming data will be provided as it becomes available during conduct of the study. Participants with vaccine-induced antibody will be provided with the opportunity for HIV antibody testing annually for five years after study completion to monitor their serological status. Participants will be counseled regarding the potential for antibody responses and the implications of such responses prior to participation in the study.

4.4 Intercurrent HIV Infection

The vaccine cannot cause HIV infection. Subjects who become HIV infected due to other causes while participating in the study will be referred for medical care for treatment and management of the disease. Policies regarding partner notification, as noted in the study consent, will be followed. The subjects may be given the opportunity to enroll in an appropriate study of acute HIV infection or a long-term follow-up study, if one is available. The NIH investigators will not be responsible for providing ongoing medical care or antiretroviral medications in the event of HIV-1 infection.

4.5 Concomitant Medications

Concomitant medications are recorded at screening and every study visit. If an FDA-approved subunit or killed vaccine is required for a medical need, then it must be given at least 14 days before or 14 days after the study injection. If it will not imperil a subject's health, FDA-approved vaccines should be deferred until at least 30 days after the study injection. Any subject who receives a study injection will continue with the clinical and laboratory evaluations specified by the study.

4.6 CRITERIA FOR WITHDRAWING A SUBJECT FROM STUDY PARTICIPATION

In VRC 010 there is only one administration of the study vaccine, which occurs on the day of enrollment. Once a subject has received the study vaccine injection, completion of all follow-up visits will be encouraged. However, subjects may choose to discontinue study participation. A subject may be discontinued from participation by the Principal Investigator for repeated failure to comply with protocol requirements or if the IRB, NIAID or the FDA require that all study visits be discontinued.

4.7 CRITERIA FOR STOPPING STUDY

The Principal Investigator will closely monitor and analyze study data as they become available and will make determinations regarding the presence and severity of adverse events. The administration of study injections and new enrollments will be halted and the IND sponsor promptly notified if any of the following criteria are met:

- **One** (or more) subject experiences a Grade 4 adverse event that is assessed as possibly, probably or definitely related to the vaccination;

  OR

- **One** (or more) subject experiences a Grade 3 adverse event assessed as possibly, probably or definitely related to the vaccination: this criterion applies to erythema, induration, fever, vomiting, laboratory abnormalities or other clinical adverse experiences, EXCEPT that **Two** (or more) subjects must experience the **same** Grade 3 adverse event assessed as possibly, probably or definitely related to vaccination for the subjective local or systemic symptoms of pain/tenderness, malaise, fatigue, headache, chills, nausea, myalgia, or arthralgia;

  OR

- **One** (or more) subject experiences Grade 2 erythema or induration at the injection site;

  OR

- **Two** (or more) subjects experience the **same** Grade 2 or higher adverse event assessed as possibly, probably or definitely related to the vaccination: this criterion applies to fever, vomiting, laboratory abnormalities or other clinical adverse experiences, but does not apply to the subjective local or systemic symptoms of pain/tenderness, malaise, fatigue, headache, chills, nausea, myalgia, or arthralgia.

The study injections and enrollments would resume only if review of the adverse events that caused the halt resulted in a recommendation to permit further study injections and study enrollments. The reviews to make this decision will occur as follows:

The IND Sponsor, in consultation with the Principal Investigator, will conduct the review and make the decision to resume or close the study for any Grade 2 events leading to a halt and for the Grade 3 subjective adverse events of pain/tenderness, malaise, fatigue, headache, chills, nausea, myalgia and arthralgia that lead to a halt.

The IND Sponsor, with participation by the Principal Investigator, will consult with the FDA to conduct the review and make the decision to resume or close the study for the Grade 3 adverse events of all other types and all Grade 4 adverse events that meet the criteria for halting the study.

Safety data reports and changes in study status are submitted to the IRB promptly in accordance with Section 5.4 and institutional policy.

5. ADVERSE EVENT REPORTING

5.1 ADVERSE EVENTS

An adverse event is any unfavorable or unintended change in body structure, body function or laboratory result associated temporally with the use of study treatment, whether or not considered related to the study treatment.

5.2 SERIOUS ADVERSE EVENTS

The term "Serious Adverse Drug Experience" is defined in 21 CFR 312.32 as follows:

"Any adverse drug experience occurring at any dose that results in any of the following outcomes: Death, a life-threatening adverse drug experience, inpatient hospitalization or prolongation of existing hospitalization, a persistent or significant disability/incapacity, or a congenital anomaly/birth defect. Important medical events that may not result in death, be life-threatening, or require hospitalization may be considered a serious adverse drug experience when, based upon appropriate medical judgment, they may jeopardize the subject or require medical or surgical intervention to prevent one of the outcomes listed in this definition. Examples of such medical events include allergic bronchospasm requiring intensive treatment in an emergency room or at home, blood dyscrasias or convulsions that do not result in inpatient hospitalization, or the development of drug dependency or drug abuse."

In Section 5.3 the term "Expedited Adverse Event (EAE) encompasses the events that would be considered an SAE by the 21 CFR 312.32 definition.

5.3 ADVERSE EVENT REPORTING TO THE IND SPONSOR

Information on adverse events (AEs) is collected by Study Nurses and other clinic staff and entered into a computer database. The Principal Investigator and the Study Coordinator review these data on an ongoing basis.

The expedited adverse event (EAE) reporting requirements and definitions for this study and the methods for expedited reporting of AEs to the DAIDS Regulatory Compliance Center (RCC) Safety Office are defined in "The Manual for Expedited Reporting of Adverse Events to DAIDS" (DAIDS EAE Manual), dated May 6, 2004. The DAIDS EAE Manual is available on the RCC website: http://rcc.tech-res-intl.com/.

AEs reported on an expedited basis must be documented on the DAIDS Expedited Adverse Event Reporting Form (EAE Reporting Form) available on the RCC website: http://rcc.tech-res-intl.com. RCC contact information is provided in Appendix II.

EAE Reporting Level:

This study uses the Standard Level of expedited AE reporting as defined in the DAIDS EAE Manual. Briefly summarized, Standard Level reporting requires completion of an EAE report form for the following types of AEs occurring after exposure to study agent:

- Result in death regardless of relationship to study agent.
- Are congenital anomalies, birth defects or fetal losses regardless of relationship to study agent.
- Result in persistent or significant disabilities or incapacities regardless of relationship to study agent.
- Are a suspected adverse drug reaction (i.e. definitely, probably, possibly, or probably not related to study agent) that requires hospitalization or prolongs existing hospitalization OR requires intervention to prevent significant/permanent disability or death.
- Are life-threatening (including all grade 4 adverse events) suspected adverse drug reactions (i.e., assessed as definitely, probably, possibly or probably not related to study agent).

In addition, any event, regardless of grade, which in the judgment of a site investigator represents a serious adverse event, may be reported to the IND sponsor as an EAE.

<u>EAE Reporting Period</u>:

AEs must be reported on an expedited basis at the Standard Level during the protocol-defined EAE Reporting Period, which for this study is the entire study duration for an individual subject (from study enrollment until study completion or discontinuation of the subject from study participation for any reason).

After the end of the protocol-defined EAE reporting period stated above, the site must report serious, unexpected, clinical suspected adverse vaccine reactions if the study site staff becomes aware of the event on a passive basis, i.e. from publicly available information.

<u>Study Agents for Expedited Reporting to DAIDS</u>:

The study agent that must be considered when determining relationships of AEs requiring expedited reporting to DAIDS is VRC-HIVADV014-00-VP.

<u>Grading Severity of Events</u>:
The Table for Grading the Severity of Adult Adverse Events is: "The Division of AIDS Table for Grading the Severity of Adult and Pediatric Adverse Events, Version 1.0, Dec 2004" (see Appendix IV).

The EAE report must be reported on the EAE form and submitted by the clinical site to the IND sponsor (DAIDS) through the Regulatory Compliance Center (RCC) Safety Office as soon as possible, but no later than 3 working days after the clinical site becomes aware of events meeting these criteria. The IND sponsor is responsible for submitting IND safety reports to the FDA, as necessary, per 21 CFR 312.32. DAIDS submits IND safety reports as soon as possible, but no later than 15 days after initial receipt of the information.

5.4 Adverse Event Reporting to the Institutional Review Board

- Investigators will submit a completed serious adverse event (SAE) report to the NIAID IRB within 7 days after becoming aware of a subject death, a potentially life-threatening (Grade 4) serious adverse event that is possibly, probably or definitely related to investigational agent, an inpatient hospitalization (other than elective), a persistent or significant disability/incapacity, or a congenital anomaly/birth defect.
- Investigators will submit a completed serious adverse event report to the NIAID IRB within 15 days after becoming aware of any severe (Grade 3) serious adverse event that is possibly, probably or definitely related to investigational agent.
- Investigators will report within 15 days on any other event or condition regardless of grade, which in their judgment represents an event reportable to the IRB.
- Investigators will forward all IND safety reports and related FDA communications to the IRB within 15 days of receipt.
- A summary of all adverse events will be reported to the NIAID IRB with submission of a request for continuing review.

5.5 Serious Adverse Event Reporting to the Institutional Biosafety Committee

The NIH Institutional Biosafety Committee (IBC) has a responsibility to review research using recombinant DNA for compliance with NIH Guidelines. In keeping with IBC requirements, any SAE reports sent to the IRB will be provided to the IBC at the same time.

6. STATISTICAL CONSIDERATIONS

6.1 Overview

This study is a single-center trial to assess the safety and tolerability of a single booster injection of a multiclade HIV adenoviral vector vaccine expressing HIV-1 Gag/Pol polyproteins from clade B and Env glycoproteins from clades A, B, and C, in HIV-uninfected adults who were previously primed with three injections of 4 mg of VRC-HIVDNA016-00-VP. A preliminary assessment of immunogenicity will also be performed.

6.2 Objectives

The primary objective of this trial concerns safety. The secondary objectives concern immunogenicity and social impacts. The study also includes exploratory analyses for immunological parameters.

6.3 Endpoints

6.3.1 Safety

Assessment of product safety will include clinical observation and monitoring of hematological and chemical parameters. Safety will be monitored after injection and evaluated by six or more clinical visits in the first 24 weeks. See Section 4.2 and Appendix III for details and specified time points.

The following parameters will be assessed:

- Local reactogenicity signs and symptoms
- Systemic reactogenicity signs and symptoms
- Laboratory measures of safety
- Adverse and serious adverse experiences

6.3.2 Immunogenicity

The principal immunogenicity endpoints are measured at Week 0 (baseline), Week 2 (for cellular immune responses only), Week 4 and Week 6. These consist of HIV-1-specific T cell responses, as measured by intracellular cytokine staining (ICS) assays and ELISPOT and HIV-1-specific humoral immune responses as measured by vaccine antigen-specific ELISA and neutralizing antibody assays. An additional endpoint, Ad5-specific immunity, will be measured by neutralizing antibody assay at Week 0 and Week 4.

6.3.3 Social Impacts

Social impact variables, as measured by questionnaire at Week 24, include any negative experiences or problems the participant experienced due to his/her participation in this study. The following social impacts will be followed during the course of the study: personal relationships, travel or immigration, employment, education, medical or dental care, health insurance, life insurance, housing, military/other government agency and other impacts identified by a participant.

6.4 SAMPLE SIZE AND ACCRUAL GUIDELINES

Recruitment is limited to subjects who previously participated in VRC 007 and received three injections of VRC-HIVDNA016-00-VP at 4 mg. All subjects from this study who are eligible and willing to participate may enroll. Maximum enrollment would be 14 subjects, but fewer subjects may enroll. The study is open-label and all participants receive one injection of the study vaccine.

6.4.1 Power Calculations for Safety

The goal of the safety evaluation for this study is to identify safety concerns associated with injection. Sample size calculations for safety are expressed in terms of the ability to detect serious adverse experiences. Although up to 14 subjects may be eligible to participate, many fewer may choose to participate.

The ability of the study to identify serious adverse experiences is best expressed by the maximum true rate of events that would be unlikely to be observed and the minimum true rate of events that would very likely be observed. The chance of observing at least one serious event depends on both the true underlying rate and the number of subjects who choose to participate. Probabilities of observing 0 or at least 2 serious adverse experiences are presented in Table 6.1.

Table 6-1: Probability of 0 or at least 2 events for different rates and sample sizes

	n=14		n=12		n=9	
True Event rate	Pr(0/14)	Pr(2+/14)	Pr(0/12)	Pr(2+/12)	Pr(0/9)	Pr(2+/9)
0.005	0.932	0.002	0.942	0.002	0.956	.001
0.010	0.869	0.008	0.886	0.006	0.914	0.003
0.035	0.607	0.084	0.652	0.064	0.726	0.037
0.050	0.488	0.153	0.540	0.118	0.630	0.071
0.100	0.229	0.415	0.282	0.341	0.387	0.225
0.150	0.103	0.643	0.142	0.557	0.232	0.401
0.200	0.044	0.802	0.069	0.725	0.134	0.564
0.300	0.007	0.953	0.014	0.915	0.040	0.804
0.400	0.001	0.992	0.002	0.980	0.010	0.929

6.5 STATISTICAL ANALYSIS

The primary analysis will consist of constructing an exact 95% confidence interval for the rate of serious adverse events. Table 6-2 gives the upper and lower bounds for 95% exact binomial confidence intervals for 0, 1 and 2 events for total participant numbers. If 14 participants enroll and none experience serious adverse experiences to the vaccine, the 95% exact 2-sided upper confidence bound for the rate of such reactions in the population is 0.232.

Table 6-2: 95% Confidence Intervals for 0, 1, or 2 observed events

		95% Confidence Interval	
N	Observed Proportion	Lower Bound	Upper Bound
14	0/14	0	0.232
	1/14	0.002	0.339
	2/14	0.018	0.428
12	0/12	0	0.265
	1/12	0.002	0.385
	2/12	0.021	0.484
9	0/9	0	0.336
	1/9	.003	0.482
	2/9	0.028	0.600

6.5.1 Analysis Variables

The analysis variables consist of baseline variables, safety variables, immunogenicity and social impact variables for primary and exploratory analyses.

6.5.2 Safety Analysis

Reactogenicities
The number and percentage of participants experiencing each type of reactogenicity sign or symptom will be tabulated by severity and vaccine regimen. For a given sign or symptom, each participant's reactogenicity will be counted once under the maximum severity for all assessments.

Adverse Experiences
Adverse experiences are coded into MedDRA preferred terms. The number and percentages of participants experiencing each specific adverse event will be tabulated by severity and relationship to treatment. For the calculations in these tables, each participant's adverse experience will be counted once under the maximum severity or strongest recorded causal relationship to treatment.

A complete listing of adverse experiences for each participant will provide details including severity, relationship to treatment, onset, duration and outcome.

Local laboratory values

Boxplots of local laboratory values by treatment will be generated for baseline values and for values measured during the course of the study. Each boxplot will show the 1st quartile, the median, and the 3rd quartile. Outliers, or values outside the boxplot, will also be plotted. If appropriate, horizontal lines representing boundaries for abnormal values will be plotted.

6.5.3 Immunogenicity Analysis

Immunological data from enrolled participants will be used in exploratory analysis only. Discrete measures will be summarized with exact binomial confidence intervals and compared between the dose groups using Fisher's Exact Test and logistic regression; continuous data will be summarized graphically, transformed if necessary and modeled as appropriate for the exploratory analyses. Data from subjects who do not elect or are not eligible for apheresis may not be included in all analyses, depending on the amount of blood obtained. Data from HIV-infected participants at or post infection will be excluded.

6.5.4 Social Impact analysis

Social impacts will be tabulated by type of event and impact on quality of life. The number and percentage of participants experiencing each type of social impact will also be tabulated by impact on quality of life. For this calculation multiple events of the same type for a participant will be counted once under the maximum impact for all post-vaccination visits.

In addition, a listing will be generated of all participants who experienced a major disturbance of their quality of life due to study participation. The listing includes all social impacts experienced by these participants, descriptions of each impact, impact on quality of life and whether or not there was a resolution.

6.5.5 Interim analyses

An interim analysis of immunogenicity data will be performed after all assays up to and including Week 6 have been completed on all participants. Reports will be provided to the

Principal Investigator and other key VRC investigators solely for the purpose of informing future trial-related decisions in a timely manner. The results will remain confidential and should in no way influence the conduct of the VRC 010 trial in terms of early termination or the safety or immunogenicity endpoint assessments that occur at later timepoints.

7. PHARMACY PROCEDURES

7.1 STUDY AGENT SUPPLY

GenVec, Inc. (Gaithersburg, MD) is the manufacturer of the investigational vaccine, VRC-HIVADV014-00-VP. It was produced under current Good Manufacturing Practices (cGMP) conditions by a contractor, Molecular Medicine (San Diego, CA). The multiclade adenoviral vector vaccine product, VRC-HIVADV014-00-VP, is a 3:1:1:1 ratio of the adenoviral vectors that encode for HIV-1 Gag/Pol polyprotein from clade B and HIV-1 Env glycoproteins from clades A, B, and C, respectively.

Single use vials will be sent unblinded to the NIH Clinical Center pharmacy. The vial label notes a storage temperature of -10° C to -25° C. The product may be stored in a freezer that has temperatures as low as -30°C. However, if deviations in storage temperature below -30°C or above -10° C occur, the site pharmacist must report the storage temperature deviation promptly to the IND sponsor. The product is shipped on dry ice, during which the product temperature is maintained at ≤ -60°C. Prior to shipping, the vials are sealed in Mylar bags to prevent CO_2 from inactivating the adenoviral vector product. The product vials should not be kept on dry ice without this protection. Once the VRC-HIVADV014-00-VP product is received at the clinical site it should be removed promptly from the dry ice package and the Mylar bag and stored as noted above.

The investigational vaccine vials will be provided at a concentration of 1 x 10^{10} PU/mL and each vaccine vial will contain 1.2 mL/vial. No diluent is needed to prepare study injections. Vials may be stored for the duration of the study (not to exceed two years).

7.2 STUDY AGENT PREPARATION

The pharmacy will record receipt of the vaccine vials containing VRC-HIVADV014-00-VP and will store the vials at the designated temperatures until use. Vials of vaccine will be allowed to equilibrate to room temperature prior to injection.

The study pharmacist will prepare an injection of the 1 x 10^{10} PU dose. The vial will be thawed at room temperature and a 1 mL injection prepared. No dilution is needed. The injection must be administered within 4 hours after removing the vial from the freezer.

Individual syringes will be prepared by the pharmacy and labeled with the subject identifier for transport to the clinic. The clinician administering the injection will select a 21-gauge needle, with a length of 1.0 or 1.5 inch (depending on subject arm size) in order to ensure intramuscular injection.

7.3 STUDY AGENT LABELING

Vials of study agent will be individually labeled with the name of the material, dosage, pH, volume, lot number, concentration, storage instructions, Investigational Use Statement

("Caution: New Drug – Limited by Federal Law to Investigational Use"), and manufacturer information. If necessary, additional lots of vaccine will be produced.

7.4 STUDY AGENT ACCOUNTABILITY

7.4.1 Documentation

The study pharmacist will be responsible for maintaining an accurate record of the codes, inventory, and an accountability record of vaccine supplies for this study. Electronic documentation as well as paper copies will be used.

7.4.2 Disposition

The empty vials and the unused portion of a vial will be discarded in a biohazard containment bag and incinerated. Any unopened vials that remain at the end of the study will be returned to the production facility or discarded at the discretion of the sponsor. Additional details regarding the vaccine are included in the Investigator's Brochure. Partially used vials will not be administered to other subjects or used for *in vitro* experimental studies. They will be disposed of in accordance with institutional or pharmacy policy.

8. HUMAN SUBJECT PROTECTIONS AND ETHICAL OBLIGATIONS

8.1 INFORMED CONSENT

The study informed consent is provided in Appendix I. It describes the investigational product to be used and all aspects of protocol participation.

Before a subject's participation in the study, it is the investigator's responsibility to obtain written informed consent from the subject, after adequate explanation of the aims, methods, anticipated benefits, and potential hazards of the study and before any protocol-specific procedures or study medications are administered.

The acquisition of informed consent will be documented in the subject's medical records, as required by 21 CFR 312.62. The informed consent form will be signed and personally dated by the subject and the person who conducted the informed consent discussion. The original signed informed consent form will be retained in the medical chart and a copy will be provided to the subject.

8.2 RISKS AND BENEFITS

8.2.1 Risks

Risks of Vaccination**:** Potential side effects resulting from intramuscular injection include stinging, arm discomfort, redness or induration of the skin at vaccine injection sites. Study subjects can self-administer medications such as acetaminophen, nonsteroidal anti-inflammatory drugs (NSAIDs), or antihistamines as required. Glucocorticoids will not be used in these study subjects unless clinically indicated.

Subjects may exhibit general signs and symptoms associated with administration of a vaccine injection, including fever, chills, rash, aches and pains, nausea, headache, dizziness and fatigue. These side effects will be monitored, but are generally short term and do not require treatment.

As with all vaccines, an allergic reaction is possible.

There is limited human experience with VRC-HIVADV014-00-VP. The study vaccine has previously been administered at the NIH Clinical Center in the first Phase I study in humans. One subject with a history of intermittent low neutrophil count had a neutrophil count that was moderately below normal shortly after vaccination. This returned to normal without any symptoms of illness. One subject had moderate diarrhea for a day. A different subject was noted to have persistent grade 1 ALT starting 25 days after study injection. An evaluation diagnosed fatty liver (steatohepatitis). There are no clinical symptoms. The condition may have existed prior to study enrollment and the subject's alcohol use and recent weight gain may be contributing factors. One subject with a history of a single seizure 3 years prior to study enrollment, experienced a seizure 64 days after study injection. This subject now has a diagnosis of epilepsy and is on anticonvulsant therapy. Given the medical history and timing of the seizure, a relationship to study vaccine seems unlikely, but cannot be ruled out. Other subjects have had mild temporary changes in blood or urine tests. It is unknown whether the lab test changes, diarrhea, fatty liver, or seizure were due to vaccine or to other factors or to a combination of the vaccine with other factors. At the 10^{10} PU dose, which will be used in this study, none of the subjects in the first Phase I study had fever and the other reactogenicity was mild or none. At the next higher dose (10^{11} PU) four subjects had a flu-like set of symptoms with fever, headache, muscle aches, malaise and chills starting 12-16 hours after vaccination and lasting a few hours. Some of these symptoms were moderate in severity. A few subjects have had nausea. Some subjects have had injection site pain in the first few days after a vaccination. These symptoms improve after treatment with over-the-counter medicine. The first extramural study using this vaccine opened in November 2004.

In preclinical testing in rabbits, this investigational vaccine was associated with fever the day after injection and decreased food consumption 1-2 days after injection. After a second injection, inflammation in the muscle around the injection site was noted, including tissue around capillaries and lymphatics near the sciatic nerve, however no nerve damage was noted. Transient and asymptomatic changes in cholesterol and triglycerides were not associated with clinical pathology and transient increase in creatine phosphokinase (CPK) was possibly related to the muscle inflammation.

The effect of this vaccine on a fetus or nursing baby is unknown, so female subjects of child bearing potential will be required to agree to use birth control for sexual intercourse beginning 21 days prior to enrollment and continuing through the last protocol visit at Week 24. Women who are pregnant or nursing will be excluded from the study.

The vaccine may cause a positive HIV antibody test using the standard screening test. A positive or indeterminate test may have a negative employment or social impact. Western blot analysis and HIV PCR or other testing will be done to either exclude or confirm HIV infection.

The vaccine may cause a positive adenovirus serotype 5 antibody test. There is a theoretical risk that this may cause the subject to be ineligible for future adenoviral vectored products or have a reduced response to such products.

<u>Risks of Blood Drawing</u>: Blood drawing may cause pain, bruising, and, rarely, infection at the site where the blood is taken.

Risks of Apheresis: Certain adverse events during apheresis procedures are expected, such as vasovagal episodes (lightheadedness, dizziness, syncope, nausea, vomiting) related to needle insertion, and cutaneous paresthesias, chills, nausea, and heartburn caused by the citrate anticoagulant used during the procedure. Hematoma formation and transient cutaneous neurological complaints related to needle insertion might also be seen. These events occur in 1-2% of donations from healthy volunteer blood donors. Vasovagal reactions are handled by postural manipulation and fluid administration. Citrate reactions are usually relieved by slowing the rate of the anticoagulant infusion and by administering oral calcium carbonate tablets (such as Tums®). Rarely, machine malfunction may result in loss of as much as a single unit (500 mL) of whole blood.

Both as reported by others and as observed at the NIH Clinical Center, subjects may rarely sustain a drop in total lymphocyte count (and $CD4^+$ T cell count) when lymphapheresis is performed frequently over a short period of time. However, participants in this study will have no more than one apheresis procedure so this is not expected to occur following the single apheresis in this study.

Other Risks: Subjects may believe that this vaccine provides protection, and therefore practice riskier behavior. Subjects will receive extensive counseling throughout the study to address this potential problem.

8.2.2 Benefits

It is unknown if any benefit will result from study participation. Others may benefit from knowledge gained in this study that may aid in the development of an HIV vaccine.

8.3 INSTITUTIONAL REVIEW BOARD

A copy of the protocol, proposed informed consent form, other written subject information, and any proposed advertising material will be submitted to the IRB for written approval.

The investigator must submit and, where necessary, obtain approval from the IRB for all subsequent protocol amendments and changes to the informed consent document. The investigator will notify the IRB of deviations from the protocol and serious adverse events.

The investigator will be responsible for obtaining IRB approval of the annual Continuing Review throughout the duration of the study.

8.4 SUBJECT CONFIDENTIALITY

The investigator must ensure that the subject's anonymity is maintained. Subjects will not be identified in any publicly released reports on this study. All records will be kept confidential to the extent provided by federal, state and local law. Medical records are made available for review when required by the Food and Drug Administration or other authorized users, such as the vaccine manufacturer, only under the guidelines set by the Federal Privacy Act. Direct access includes examining, analyzing, verifying, and reproducing any records and reports that are important to the evaluation of the study. The investigator is obligated to inform the subjects that the above-named representatives will review their study-related records without violating the confidentiality of the subjects.

8.5 Plan for Use and Storage of Biological Samples

Stored study research samples are labeled by a code (such as a number) that only the VRC Clinic team can link to the subject. All stored research samples are logged into the VRC Laboratory Information Management System (LIMS) database and uses of these samples are documented in the LIMS. The requirement to maintain subject confidentiality is included in the study informed consent document.

8.6 Subject Identification and Enrollment of Study Participants

All study activities will be carried out at the Clinical Center at the National Institutes of Health. Study subjects will be contacted on the basis of consent granted during participation in study VRC 007 (04-I-0254). Effort will be made to include all subjects who are willing and eligible to participate.

8.6.1 Participation of Children

Children are not eligible to participate in this clinical trial because eligibility depends on prior participation in VRC 007 (04-I-0254) and no children participated in that study.

8.7 Compensation Plan for Subjects

Subjects will be compensated for time and inconvenience in accordance with the standards for compensation of the Clinical Research Volunteer Program. The compensation per visit will be $100 for the visit that includes a study injection, $140 if an apheresis is performed at the Week 4 visit and $70 for other clinic visits. The approximate total compensation for the subject will be $450 if the schedule is completed without apheresis and $520 if an apheresis is included. These estimates are based on the number of study visits, one study injection, and the possibility that one apheresis procedure may be completed.

8.8 Safety Monitoring

Close cooperation between the designated members of the Protocol Team will occur to evaluate and respond to individual adverse events in a timely manner. Designated team members (Principal Investigator, Medical Officer, Protocol Specialist, Study Coordinator and other study clinicians) will review the summary study safety data reports on a weekly basis until all subjects have completed 4 weeks vaccination follow-up and then on a monthly basis until study completion. This monitoring is conducted in order to be certain that the vaccine booster injection has an acceptable safety profile.

9. ADMINISTRATIVE AND LEGAL OBLIGATIONS

9.1 Protocol Amendments and Study Termination

Protocol Amendments must be made only with the prior approval of the National Institute of Allergy and Infectious Diseases' Division of AIDS and Vaccine Research Center. Agreement from the investigator must be obtained for all protocol amendments and amendments to the informed consent document. All study amendments will be submitted to the IRB for approval.

The Division of AIDS, National Institute of Allergy and Infectious Diseases, the Vaccine Research Center, the Principal Investigator and the Food and Drug Administration reserve the right to terminate the study. The investigator will notify the IRB in writing of the study's

completion or early termination.

9.2 Study Documentation and Storage

The investigator will maintain a list of appropriately qualified persons to whom trial duties have been delegated.

Source documents are original documents, data, and records from which the subject's data are obtained. These include but are not limited to hospital records, clinical and office charts, laboratory and pharmacy records, diaries, microfiches, radiographs, and correspondence.

The investigator and staff are responsible for maintaining a comprehensive and centralized filing system of all study-related (essential) documentation, suitable for inspection at any time by representatives from the National Institute of Allergy and Infectious Diseases' Division of AIDS and Vaccine Research Center, IRB, FDA, and/or applicable regulatory authorities. Elements include:

- Subject files containing completed informed consent forms, and supporting copies of source documentation (if kept)
- Study files containing the protocol with all amendments, Investigator Brochures, copies of all correspondence with the IRB and the National Institute of Allergy and Infectious Diseases' Division of AIDS and Vaccine Research Center

In addition, all original source documentation must be maintained and be readily available.

All essential documentation should be retained by the institution for the same period of time required for medical records retention. The FDA requires study records to be retained for up to two years after marketing approval or refusal (21 CFR 312.62). No study document should be destroyed without prior written agreement between the National Institute of Allergy and Infectious Diseases' Division of AIDS and Vaccine Research Center and the investigator. Should the investigator wish to assign the study records to another party or move them to another location, they must notify the National Institute of Allergy and Infectious Diseases' Division of AIDS and Vaccine Research Center in writing of the new responsible person and/or the new location.

9.3 Data Collection and Protocol Monitoring

9.3.1 Data Collection

VRC Clinic staff will collect clinical research data in a secure electronic data management system through a contract research organization, EMMES (Rockville, MD). Extracted data without patient identifiers will be sent to the Protocol Statistician for statistical analysis.

9.3.2 Protocol Monitoring Plan

The Division of AIDS, as the IND Sponsor and the Vaccine Research Center, as the protocol sponsor and developer of the study agents, are both part of the National Institute of Allergy and Infectious Diseases. Regulatory authority inspectors or authorized representatives of the study sponsors are responsible for contacting and visiting the investigator for the purpose of inspecting the facilities and, upon request, inspecting the various records of the trial, provided that subject confidentiality is respected.

Site investigators will allow the Study Monitors, the NIAID IRB, and the FDA to inspect study documents (e.g., consent forms, drug distribution forms, case report forms) and pertinent hospital or clinic records for confirmation of the study data.

Site visits by study monitors will be made in accordance with the IND Sponsor (DAIDS) policy to monitor the following: study operations, the quality of data collected in the research records, the accuracy and timeliness of data entered in the database, and to determine that all process and regulatory requirements are met. Study monitoring site visits occur at initiation of the study, at intervals determined by the IND sponsor during conduct of the study, and at completion of the study.

9.4 LANGUAGE

All written information and other material to be used by subjects and investigative staff must use vocabulary and language that are clearly understood.

9.5 POLICY REGARDING RESEARCH-RELATED INJURIES

The Clinical Center will provide short-term medical care for any injury resulting from participation in this research. In general, the National Institutes of Health, the Clinical Center, or the Federal Government will provide no long-term medical care or financial compensation for research-related injuries.

10. REFERENCES

1. CDC, Increases in HIV diagnoses--29 States, 1999-2002. *MMWR Morb Mortal Wkly Rep*, 2003. **52**(47): 1145-8.
2. UNAIDS, AIDS Epidemic Update December 2003. 2003.
3. WHO, *Treating 3 Million by 2005: The WHO Strategy*. 2003, World Health Organization: Geneva, Switzerland. p. 1-53.
4. Approaches to the development of broadly protective HIV vaccines: challenges posed by the genetic, biological and antigenic variability of HIV-1: Report from a meeting of the WHO-UNAIDS Vaccine Advisory Committee Geneva, 21-23 February 2000. *AIDS*, 2001. **15**(6): W1-W25.
5. Osmanov S, et al., Estimated global distribution and regional spread of HIV-1 genetic subtypes in the year 2000. *J Acquir Immune Defic Syndr*, 2002. **29**(2): 184-90.
6. Nabel G, W Makgoba, and J Esparza, HIV-1 Diversity and Vaccine Development. *Science*, 2002. **296**(5577): 2335.
7. Borrow P, et al., Virus-specific CD8+ cytotoxic T-lymphocyte activity associated with control of viremia in primary human immunodeficiency virus type 1 infection. *J Virol*, 1994. **68**(9): 6103-10.
8. Koup RA, et al., Temporal association of cellular immune responses with the initial control of viremia in primary human immunodeficiency virus type 1 syndrome. *J Virol*, 1994. **68**(7): 4650-5.
9. Pantaleo G, et al., Major expansion of CD8+ T cells with a predominant V beta usage during the primary immune response to HIV. *Nature*, 1994. **370**(6489): 463-7.
10. Musey L, et al., Cytotoxic-T-cell responses, viral load, and disease progression in early human immunodeficiency virus type 1 infection. *N Engl J Med*, 1997. **337**(18): 1267-74.
11. Altman JD, et al., Phenotypic analysis of antigen-specific T lymphocytes. *Science*, 1996. **274**(5284): 94-6.
12. Jin X, et al., Dramatic rise in plasma viremia after CD8(+) T cell depletion in simian immunodeficiency virus-infected macaques. *J Exp Med*, 1999. **189**(6): 991-8.
13. Schmitz JE, et al., Control of viremia in simian immunodeficiency virus infection by CD8+ lymphocytes. *Science*, 1999. **283**(5403): 857-60.
14. Douek DC, et al., HIV preferentially infects HIV-specific CD4+ T cells. *Nature*, 2002. **417**(6884): 95-8.
15. Yang O, et al., Efficient lysis of human immunodeficiency virus type 1-infected cells by cytotoxic T lymphocytes. *J Virol*, 1996. **70**(9): 5799-806.
16. Klein MR, et al., Kinetics of Gag-specific cytotoxic T lymphocyte responses during the clinical course of HIV-1 infection: a longitudinal analysis of rapid progressors and long-term asymptomatics. *J Exp Med*, 1995. **181**(4): 1365-72.
17. Moss PA, et al., Persistent high frequency of human immunodeficiency virus-specific cytotoxic T cells in peripheral blood of infected donors. *Proc Natl Acad Sci U S A*, 1995.

92(13): 5773-7.

18. Nixon DF, et al., HIV-1 gag-specific cytotoxic T lymphocytes defined with recombinant vaccinia virus and synthetic peptides. *Nature*, 1988. **336**(6198): 484-7.

19. Shiver JW. *Merck's HIV vaccine program: research approaches and critical issues*. in *AIDS Vaccine 2001*. 2001. Philadelphia, PA.

20. Shiver JW, et al., Replication-incompetent adenoviral vaccine vector elicits effective anti-immunodeficiency-virus immunity. *Nature*, 2002. **415**(6869): 331-5.

21. Sullivan NJ, et al., Development of a preventive vaccine for Ebola virus infection in primates. *Nature*, 2000. **408**(6812): 605-9.

22. Casimiro DR, et al., Vaccine-induced immunity in baboons by using DNA and replication-incompetent adenovirus type 5 vectors expressing a human immunodeficiency virus type 1 gag gene. *J Virol*, 2003. **77**(13): 7663-8.

23. Sullivan NJ, et al., Accelerated vaccination for Ebola virus haemorrhagic fever in non-human primates. *Nature*, 2003.

24. Amara RR, et al., Control of a mucosal challenge and prevention of AIDS by a multiprotein DNA/MVA vaccine. *Science*, 2001. **292**(5514): 69-74.

25. Plotkin SA and WA Orenstein, *Vaccines*. 3rd ed. 1999, Philadelphia: W.B. Saunders Co. xix, 1230.

26. Schwartz AR, Y Togo, and RB Hornick, Clinical evaluation of live, oral types 1, 2, and 5 adenovirus vaccines. *Am Rev Respir Dis*, 1974. **109**(2): 233-9.

27. Rosenbaum MJ, JW Poudstone, and EA Edwards, Acceptability and antigenicity of adjuvant soluble sub-unit adenovirus vaccines in navy recruits. *Mil Med*, 1976. **141**(6): 383-8.

28. Rosenbaum MJ, EA Edwards, and DC Hoeffler, Recent experiences with live adenovirus vaccines in navy recruits. *Mil Med*, 1975. **140**(4): 251-7.

29. Gaydos CA and JC Gaydos, Adenovirus vaccines in the U.S. military. *Mil Med*, 1995. **160**(6): 300-4.

30. Harvey BG, et al., Airway epithelial CFTR mRNA expression in cystic fibrosis patients after repetitive administration of a recombinant adenovirus. *J Clin Invest*, 1999. **104**(9): 1245-55.

31. Bellon G, et al., Aerosol administration of a recombinant adenovirus expressing CFTR to cystic fibrosis patients: a phase I clinical trial. *Hum Gene Ther*, 1997. **8**(1): 15-25.

32. Hay JG, et al., Modification of nasal epithelial potential differences of individuals with cystic fibrosis consequent to local administration of a normal CFTR cDNA adenovirus gene transfer vector. *Hum Gene Ther*, 1995. **6**(11): 1487-96.

33. Harvey BG, et al., Cellular immune responses of healthy individuals to intradermal administration of an E1-E3- adenovirus gene transfer vector. *Hum Gene Ther*, 1999. **10**(17): 2823-37.

34. Rosengart TK, et al., Six-month assessment of a phase I trial of angiogenic gene therapy

for the treatment of coronary artery disease using direct intramyocardial administration of an adenovirus vector expressing the VEGF121 cDNA. *Ann Surg*, 1999. **230**(4): 466-70; discussion 70-2.

35. Rosengart TK, et al., Angiogenesis gene therapy: phase I assessment of direct intramyocardial administration of an adenovirus vector expressing VEGF121 cDNA to individuals with clinically significant severe coronary artery disease. *Circulation*, 1999. **100**(5): 468-74.

36. Sterman DH, et al., Adenovirus-mediated herpes simplex virus thymidine kinase/ganciclovir gene therapy in patients with localized malignancy: results of a phase I clinical trial in malignant mesothelioma. *Hum Gene Ther*, 1998. **9**(7): 1083-92.

37. Clayman GL, et al., Adenovirus-mediated wild-type p53 gene transfer as a surgical adjuvant in advanced head and neck cancers. *Clin Cancer Res*, 1999. **5**(7): 1715-22.

38. Swisher SG, et al., Adenovirus-mediated p53 gene transfer in advanced non-small-cell lung cancer. *J Natl Cancer Inst*, 1999. **91**(9): 763-71.

39. Gahery-Segard H, et al., Phase I trial of recombinant adenovirus gene transfer in lung cancer. Longitudinal study of the immune responses to transgene and viral products. *J Clin Invest*, 1997. **100**(9): 2218-26.

40. Tursz T, et al., Phase I study of a recombinant adenovirus-mediated gene transfer in lung cancer patients. *J Natl Cancer Inst*, 1996. **88**(24): 1857-63.

41. Crystal RG, et al., Analysis of risk factors for local delivery of low- and intermediate-dose adenovirus gene transfer vectors to individuals with a spectrum of comorbid conditions. *Hum Gene Ther*, 2002. **13**(1): 65-100.

42. Rajagopalan S, et al., Phase I study of direct administration of a replication deficient adenovirus vector containing the vascular endothelial growth factor cDNA (CI-1023) to patients with claudication. *Am J Cardiol*, 2002. **90**(5): 512-6.

43. Harvey BG, et al., Safety of local delivery of low- and intermediate-dose adenovirus gene transfer vectors to individuals with a spectrum of morbid conditions. *Hum Gene Ther*, 2002. **13**(1): 15-63.

44. Assessment of adenoviral vector safety and toxicity: report of the National Institutes of Health Recombinant DNA Advisory Committee. *Hum Gene Ther*, 2002. **13**(1): 3-13.

45. Raper SE, et al., A pilot study of in vivo liver-directed gene transfer with an adenoviral vector in partial ornithine transcarbamylase deficiency. *Hum Gene Ther*, 2002. **13**(1): 163-75.

46. Yang Y and JM Wilson, Clearance of adenovirus-infected hepatocytes by MHC class I-restricted CD4+ CTLs in vivo. *J Immunol*, 1995. **155**(5): 2564-70.

47. Yang Y, et al., Cellular immunity to viral antigens limits E1-deleted adenoviruses for gene therapy. *Proc Natl Acad Sci U S A*, 1994. **91**(10): 4407-11.

48. Nunes FA, et al., Gene transfer into the liver of nonhuman primates with E1-deleted recombinant adenoviral vectors: safety of readministration. *Hum Gene Ther*, 1999. **10**(15): 2515-26.

49. Lozier JN, et al., Toxicity of a first-generation adenoviral vector in rhesus macaques. *Hum Gene Ther*, 2002. **13**(1): 113-24.

50. Morral N, et al., Lethal toxicity, severe endothelial injury, and a threshold effect with high doses of an adenoviral vector in baboons. *Hum Gene Ther*, 2002. **13**(1): 143-54.

51. Varnavski AN, et al., Preexisting immunity to adenovirus in rhesus monkeys fails to prevent vector-induced toxicity. *J Virol*, 2002. **76**(11): 5711-9.

52. Harvey BG, et al., Variability of human systemic humoral immune responses to adenovirus gene transfer vectors administered to different organs. *J Virol*, 1999. **73**(8): 6729-42.

53. Harvey BG, et al., Host responses and persistence of vector genome following intrabronchial administration of an E1(-)E3(-) adenovirus gene transfer vector to normal individuals. *Mol Ther*, 2001. **3**(2): 206-15.

54. Betts MR, JP Casazza, and RA Koup, Monitoring HIV-specific CD8+ T cell responses by intracellular cytokine production. *Immunol Lett*, 2001. **79**(1-2): 117-25.

55. Helms T, et al., Direct visualization of cytokine-producing recall antigen-specific CD4 memory T cells in healthy individuals and HIV patients. *J Immunol*, 2000. **164**(7): 3723-32.

56. Roederer M, et al., Flow cytometric analysis of vaccine responses: how many colors are enough? *Clin Immunol*, 2004. **110**(3): 199-205.

57. Betts MR, et al., Putative immunodominant human immunodeficiency virus-specific CD8(+) T-cell responses cannot be predicted by major histocompatibility complex class I haplotype. *J Virol*, 2000. **74**(19): 9144-51.

58. Malenbaum SE, D Yang, and C Cheng-Mayer, Evidence for similar recognition of the conserved neutralization epitopes of human immunodeficiency virus type 1 envelope gp120 in humans and macaques. *J Virol*, 2001. **75**(19): 9287-96.

59. Mascola JR, et al., Human immunodeficiency virus type 1 neutralization measured by flow cytometric quantitation of single-round infection of primary human T cells. *J Virol*, 2002. **76**(10): 4810-21.

60. Sprangers MC, et al., Quantifying adenovirus-neutralizing antibodies by luciferase transgene detection: addressing preexisting immunity to vaccine and gene therapy vectors. *J Clin Microbiol*, 2003. **41**(11): 5046-52.

61. Seth P, *Adenoviruses: basic biology to gene therapy*. Medical intelligence unit. 1999, Austin, TX: R.G. Landes Co.

62. Brough DE, et al., A gene transfer vector-cell line system for complete functional complementation of adenovirus early regions E1 and E4. *J Virol*, 1996. **70**(9): 6497-501.

63. Rasmussen H, et al., TNFerade Biologic: preclinical toxicology of a novel adenovector with a radiation-inducible promoter, carrying the human tumor necrosis factor alpha gene. *Cancer Gene Ther*, 2002. **9**(11): 951-7.

APPENDIX I
INFORMED CONSENT FORM

INSTITUTE: Vaccine Research Center, National Institute of Allergy and Infectious Diseases

STUDY NUMBER: VRC 010 PRINCIPAL INVESTIGATOR: Andrew Catanzaro, M.D.

STUDY TITLE: A Phase I Clinical Trial to Evaluate the Safety and Immunogenicity of a Booster Dose of a Recombinant Multiclade HIV-1 Adenoviral Vector Vaccine, VRC-HIVADV014-00-VP, in Uninfected Subjects who were Previously Immunized with VRC-HIVDNA016-00-VP in VRC 007 (04-I-0254)

Latest IRB Review:
Latest Amendment Approved:

INTRODUCTION

We invite you to take part in a research study at the National Institutes of Health (NIH).

First, we want you to know that:

> Taking part in NIH research is entirely voluntary.
>
> You may choose not to take part, or you may withdraw from the study at any time. In either case, you will not lose any benefits to which you are otherwise entitled. However, to receive care at the NIH, you must be taking part in a study or be under evaluation for study participation.
>
> You may receive no benefit from taking part. The research may give us knowledge that may help people in the future.

Second, some people have personal, religious or ethical beliefs that may limit the kinds of medical or research treatments they would want to receive (such as blood transfusions). If you have such beliefs, please discuss them with your NIH doctors or research team before you agree to the study.

Now we will describe this research study. Before you decide to take part, please take as much time as you need to ask any questions and discuss this study with anyone at NIH, or with family, friends or your personal physician or other health professional.

PURPOSE OF THE STUDY

You are invited to participate in this study, called VRC 010. You may enroll because you were in a study called VRC 007 (04-I-0254). In VRC 007 you received an experimental HIV vaccine called VRC-HIVDNA016-00-VP. This is a “DNA vaccine.” In VRC 010 you will get a different experimental HIV vaccine called VRC-HIVADV014-00-VP. This is an “adenoviral vector vaccine.” The STUDY VACCINE section of the consent describes the vaccine.

"Experimental" means that the study vaccine has not been approved by the Food and Drug Administration (FDA) for treating or preventing HIV infection. The FDA allows its use in research only. The experimental vaccine in this study has been given to about 100 people in previous studies.

This is one of two studies going on at the same time in which people will get both of these experimental vaccines. These two studies will be the first time these two vaccines will be used in a combined vaccination schedule in people. The purpose of the study is to see if the experimental vaccination schedule with the DNA vaccine given first and then the adenoviral vector vaccine is safe and if there are any side effects. Research laboratories will do many tests from blood samples to see how the body responds to the vaccines. Information about whether there is any social harm that results from participating in a study of an HIV vaccine will also be collected. Social harms are such things as problems with insurance, health care, friends, family, employment, housing, education, or government agencies.

This study will not answer the question of whether this experimental vaccine works to prevent HIV infection. It is unknown if the study vaccine works.

You are eligible to participate in this study because:

- You were enrolled in the study VRC 007
- You received three vaccinations in VRC 007.
- You did not have any serious side effects caused by vaccinations in VRC 007.
- You have completed screening and are able to attend clinic visits over the next 24 weeks.
- Your screening blood tests do not include any findings that prevent your participation.
- Your physical exam does not include any findings that prevent your participation.
- Your medical history of recent treatments and past medical conditions do not include any findings that prevent your participation.
- You are not HIV-infected, are willing to have HIV tests, are willing to have HIV risk-reduction counseling and are willing to follow NIH guidelines for partner notification if you have a diagnosis of HIV infection.
- You are willing to donate blood samples for future research
- You have completed an assessment of understanding of the study.
- If you are a woman: you are not pregnant or breast-feeding a child. You are not planning to become pregnant in the next 24 weeks.

In this study, one injection (shot) of the study vaccine will be given in your upper arm. Up to 14 people may participate in this study. The study will be done at the NIH Clinical Center. The study will last about 24 weeks. During the study you will be checked for vaccine side effects. You will be treated at the National Institutes of Health if any side effects occur.

You will be told of any new information learned during this study that may be important to your health. At the end of the study, you will be told when study results may be available and how to learn about the study results.

STUDY VACCINE

Vaccines are substances used to create immune responses (resistance) to a disease. The hope is that the immune response to a vaccine will prevent the disease. It is not known if the study vaccine prevents HIV infection.

The study vaccine is a modified adenovirus that carries DNA into the muscle cells. DNA is the material from which genes are made. The DNA provides the code (instructions) that allows a cell to produce proteins. The DNA in the study vaccine was manufactured in a laboratory. It has codes for parts of three HIV proteins called Gag, Pol, and Env. The manufactured DNA has been packaged in an adenovirus shell that is missing some of the usual adenovirus genes. The adenovirus used in this vaccine has been changed so that it cannot reproduce in a human body.

Adenovirus is a common virus that causes upper respiratory infections (such as the common cold), eye infection (conjunctivitis), urine infection or diarrhea. You cannot get an adenovirus infection from the study vaccine because of the way it has been changed. You cannot infect someone else with the study vaccine adenovirus. An adenovirus with inserted DNA in a laboratory is called a vector or adenoviral vector.

Many vaccines are made of proteins injected directly into muscle. An immune response happens when the muscle cells meet the foreign protein. The study vaccine allows Gag, Pol, and Env protein to be made in the muscle cell. This is an important feature of the study vaccine. The adenoviral vector vaccine allows the muscle cells to make the Gag, Pol and Env proteins for several days. This mimics the way the immune system sees virus proteins during a virus infection. The investigators will study your immune response to these three HIV proteins by using the blood samples collected during the study.

You cannot get an HIV infection from the study vaccine. This is because there are only incomplete codes for 3 HIV proteins made from the vaccine. A live HIV virus would have complete codes for 9 proteins.

In order to create the adenoviral vector in the study vaccine, cultures derived from human fetal kidney cells were used. The cell culture was started in the 1980s. Production of the study vaccine does not require getting new fetal kidney cells. There are not any human cells in the study vaccine injection.

The National Institutes of Health, including some members of the Vaccine Research Center scientific staff, developed the investigational vaccine being used in this research study. The results of this study could play a role in whether the FDA will approve the vaccine for sale at some time in the future. If approved, the future sale of the vaccine could lead to payments to NIH and some NIH scientists. By U.S. law, government scientists are required to receive such payments for their inventions. You will not receive any money from development of the product.

STUDY PROCEDURES

If you agree to take part in the study, you will be injected with 10^{10} particle units (PU) of the study vaccine. If you get 10^{10} PU it means you will be getting ten billion (10,000,000,000) units of the vaccine.

You will be injected on the first day of your enrollment in the study. This is called Day 0. The volume of the injection is 1 mL. It will be given as a single injection in an upper arm (deltoid) muscle. The injection will be given using a syringe and needle.

The clinic staff will observe you for at least 30 minutes after the vaccination. You will be asked to complete a diary card at home for 5 days. This will require that you record your temperature and symptoms. You must also look at your arm and record information about the injection site each day for 5 days. You must call a study nurse one or two days after the injection. This is a required telephone appointment. If you do not contact a study nurse for the required follow-up by the second day, a study nurse will call you. You will have to come to the clinic if you are having a rash, hives or fever of 38.7˚C (101.7˚F) or higher. You will be asked to come in if you are having great difficulty in the activities of daily living (such as going to work or taking care of yourself). You will also need to come to the clinic for any problem which the nurse or doctor thinks should be checked by exam or blood or urine testing. It is very important that you follow the instructions given to you by the clinic staff.

You will have about 6 clinic visits during the 24 weeks you are in this study. The Day 0 visit will take about 4 hours. Most other clinic visits will usually take about 2 hours. The Week 4 visit may take longer if you have an apheresis procedure. This procedure is explained in detail in the next section of this consent. The telephone report will take about 15 minutes. The following table shows the study schedule:

	Day 0	Day 1 or 2	Week 2	Week 4	Week 6	Week 12	Week 24
Assessment of Understanding & Informed Consent	X						
Vaccine Injection	X						
Telephone Report		X					
5-day Diary Card	**(take home)**		**(Return card)**				
Clinical Evaluation (Vital Signs, lymph node exam, weight); Physical exam if needed	X		X	X	X	X	X
Medical history	X		X	X	X	X	X
Blood Sample collection	X		X	Regular Blood Draw **or** Apheresis	X	X	X
Urine Sample	X		X		X	X	X
Pregnancy Test (for females)	X						X
Counseling as needed about HIV and pregnancy avoidance	X		X	X	X	X	X
HIV Tests	X				X		X
Social Impact Questionnaire							X

At each visit, you will be checked for any health changes or problems since your last visit. You will be asked how you are feeling and what medications you are taking. Blood will be drawn at visits for testing of your health and your immune system. The amount of blood drawn will vary from about 5 tablespoons (75 mL) to 10 tablespoons (150 mL), depending on the visit. You might also be asked to have laboratory tests between regular visits if needed to evaluate a change in your health. The total amount of blood drawn during the 24 weeks of study participation will be about 3 cups (675 mL). No more than a total of about two cups (450 mL) will be drawn over any six-week period during the study. You will have to give a urine sample at some visits.

You will be informed promptly if any health concerns are identified by the tests. You may get a copy of routine blood and urine test results by asking the study nurse on your next visit. You will have HIV testing done three or more times and will be asked questions about your sexual behavior and drug use. You will also be asked to complete a "Social Impact" questionnaire during the last clinic visit at Week 24.

Apheresis

At the Week 4 visit, we would like to collect your blood sample by apheresis. In this procedure, blood will be removed through a needle in the vein of one arm. The blood will be spun in a

machine that permits separation of the desired blood component (white blood cells). Then the remainder will be returned through the same needle. Citrate, a medication to prevent blood from clotting, will be added to the blood while in the machine to prevent it from clotting. This procedure will be done at the Department of Transfusion Medicine in the NIH Clinical Center.

The purpose of the apheresis in this study is to allow the investigator to obtain a larger number of white blood cells than can be collected by simple blood drawing. Getting a lot of white blood cells will allow more laboratory tests to be done to see how the immune system responds to the study vaccine. The number of white blood cells collected is a small fraction of the total amount in your body. The body quickly replaces removed cells. Similar procedures are used daily in Blood Banks as a way of getting blood products from normal donors and as a type of therapy for certain diseases. The procedure will take about 1-3 hours.

Before apheresis is done, your weight, pulse and blood pressure will be checked. The result of your blood test at Week 2 is one factor in whether you are eligible for apheresis at Week 4. You must also weigh at least 110 pounds on the day of apheresis. You will be asked questions about your general health and medical history. You will be asked to sign the Department of Transfusion Medicine’s consent form for the apheresis procedure on the day of apheresis. You will be asked to lie on a recliner or couch. The kits used to collect the apheresis products are sterilized, single-use, disposable sets that are not in contact with any person’s body fluids other than yours. No blood products are given to you during these procedures. A doctor from the Department of Transfusion Medicine will be in or near the apheresis donor area at all times.

If you do not wish to have apheresis or if you are not eligible for apheresis or if we cannot get an apheresis appointment for your Week 4 visit, then an 80 mL (about 1/3 cup) blood sample will be collected as usual in blood collection tubes using a needle.

MONITORING OF THE STUDY
This study will be monitored by a group of physicians and scientists at the Clinical Center. This group will review the information from the study and will pay close attention to harmful reactions.

HIV TESTING
As part of your participation in this study, it will be necessary to test your blood for the presence of antibodies to the Human Immunodeficiency Virus (HIV), the virus that causes Acquired Immune Deficiency Syndrome (AIDS). If antibodies are present, other tests will be done to determine if you have HIV infection. In order to perform the test, a small amount of blood (approximately 2 teaspoons) will be withdrawn from one of your arms with a needle. You may experience some slight discomfort at the needle entry site and there may be some bruising. In addition, there is a very small risk of you fainting or of infection at the needle entry site. If you are diagnosed as having HIV infection, you should be aware of the following Clinical Center HIV Testing Policy:

1. Your physician will notify you promptly of the HIV test results.

2. Your physician and/or the Clinical Center HIV counselor will offer you, and any current and/or ongoing sexual partner(s) (spouses are generally considered to be current or ongoing sexual partners) or needle-sharing partner(s) you identify, information on the meaning of the test results and how to prevent the spread of the infection.

3. Because the virus may be transmitted in several ways, it is important that you inform sexual and/or needle-sharing partner(s) that any, or all, of them may have been exposed to the HIV virus and encourage them to be tested. If you request it, staff at the Clinical Center will assist you in notifying your partner(s) and arrange counseling for them through an HIV counselor.

4. The results of your HIV test and/or documentation of the diagnosis of AIDS will become a part of your Clinical Center medical record and, as such, will be protected from unauthorized disclosure by the Federal Privacy Act of 1974. In general, access to your medical record will be restricted to those health care professionals directly involved in your care or in the conduct of ongoing biomedical research, and information is not usually released to other third parties without your permission or that of your designated representative. However, there are some particular routine uses of such information of which you should be aware.

   (a) If you are unwilling or unable to notify your partner(s), the Clinical Center is responsible for attempting to contact and inform them of their possible exposure to the virus. Reasonable attempts will be made to protect your identity including withholding your name when notifying any partner(s) of their possible exposure. Some notification or counseling of current and/or ongoing partners may be carried out through arrangements with, or referral to, local public health agencies.

   (b) At your request, a summary of your care at the Clinical Center will be provided to your primary health care provider.

   (c) The Clinical Center may report certain communicable diseases, such as HIV infection, to appropriate State and Federal government agencies.

      i. For Clinical Center patients who are Maryland residents, the Clinical Center reports by "Patient Unique Identifier Number" (rather than by name) newly obtained HIV-positive results from its laboratory to the Maryland Department of Health and Mental Hygiene. Patient Unique Identifier Number is: last four digits of social security number, birth month, birth day, birth year, race, and gender.

      ii. For Clinical Center patients who are Maryland residents, the Clinical Center reports by name new cases of AIDS to the Maryland Department of Health and Mental Hygiene.

iii. For Clinical Center patients who are not Maryland residents, the Clinical Center reports HIV-positive results and/or AIDS to the patient's primary care physician.

If you have any questions regarding the HIV testing or the information provided above, you are encouraged to discuss them with your physician or you may call a Clinical Center HIV counselor at 301-496-2381.

GENETIC TESTING

Some of the blood drawn from you as part of this study will be used for genetic tests. Some genetic tests can help researchers study how health or illness is passed on to you by your parents or from you to your children. In vaccine research some genetic tests are done to see if different types of immune response to a vaccine seem to be related to genetic differences in people. Some of the genetic tests will be done in a research lab from your stored samples. Genetic tests done in a research lab will not be in your medical record. Tests that are done in a research lab will not have your name on the sample given to the research lab. New genetic research tests to help understand if a vaccine might work may be done on stored samples in the future.

Some genetic tests are done in a regular medical laboratory. HLA type is a genetic test ordered through the NIH Clinical Center medical laboratory. HLA type results will be in your medical record at the NIH Clinical Center.

HLA Type and Other Data Collected in VRC 007

Information about you collected during your participation in VRC 007 may also be used to analyze results of VRC 010. This includes your HLA type. HLA type is a genetic test of markers of the immune system. It is usually used to match bone marrow or organ transplants. For research, HLA testing is sometimes used to try to identify factors associated with response to a vaccine, the progression of HIV disease or related conditions. Determining HLA type is necessary to be able to perform certain research studies.

Some HLA types have been associated with an increased risk of certain diseases like arthritis and other rheumatologic problems. Simply having those HLA types, however, doesn't mean you will develop these diseases.

Genetic testing can also be used to determine if people are directly related. These tests sometimes show that people were adopted or that their biological parent is someone other than their legal parent. If these facts were not known previously they could be troubling. Additional genetics counseling and advice is available from the National Institutes of Health to help you understand the nature and implications of genetic findings about you and your family.

It is our policy to not discuss such information unless it has direct medical or reproductive implications for you or your family. By agreeing to participate in this study, you do not waive any rights that you may have regarding access to and disclosure of your records. For further information on those rights, you can contact the principal investigator of this study.

Any genetic information collected or discovered about you or your family will be confidential. Results of HLA testing will become a part of your medical record at NIH. Medical records containing this information are maintained in a secure manner. Genetic information about you will not be revealed to others, including your relatives, without your permission. We will not release any information about you or your family to any insurance company or employer unless you sign a document allowing release of information. Instances are known in which genetic information has been obtained or requested when a person applies for health insurance or a job.

STORED SAMPLES

During your participation on this study blood samples will be collected from you, as already explained. We will store these samples for future research to learn more about HIV, HIV vaccines and other medical conditions.

The results from the research done with your stored samples will not be given to your health care provider and will not be put in your medical record. This is because the test results, unlike routine medical testing, will be experimental or preliminary. The relevance of these tests to your care is unknown. At your request however, the results of any research tests will be discussed with you or your physician.

Labeling of Stored Samples

Your stored samples will be labeled by a code (such as a number) that only the study team can link to you. Any identifying information about you will be kept confidential to the extent permitted by law.

Risks from Stored Samples

The greatest risk is the unplanned release of information from your medical records. The chance that this information will be given to an unauthorized person without your permission is very small. Possible problems with the unplanned release of information include discrimination when applying for insurance and employment. Similar problems may occur if you disclose information yourself or agree to have your medical records released.

Future studies

In the future, other investigators (at NIH or outside of NIH) may wish to study your stored sample. When the study team shares your materials, they may share it with no identifying information or with a code). Some information about you, such as your gender, age, health history, or ethnicity may also be shared with other investigators. Any future research studies using your samples will be reviewed by the investigator’s Institutional Review Board (IRB), a special committee that oversees medical research studies to protect the rights and welfare of human subject volunteers.

Your stored materials will be used only for research and will not be sold. The research done with your materials may be used to develop new products in the future but you will not receive payment for such products.

POSSIBLE STUDY RISKS

Injection Risks

You may have some side effects from the injection. We will be using a standard needle and syringe to give the injection. You may have mild discomfort from the injection. There may be stinging, arm discomfort, pain, soreness, redness, and swelling. There is a risk of fainting. There is a very small chance of infection at the injection site. You will be asked to record and report any side effects you have. You may need to make extra visits to the clinic if you have side effects. You may use over-the-counter (nonprescription) medications for pain or fever, if needed. Examples of over-the-counter pain medications include Tylenol (and other generic brands containing acetaminophen) and Motrin or Advil (and other generic brands containing ibuprofen). Call the study nurse if you have questions about over-the-counter medications.

Apheresis Procedure Risks

Apheresis donations are generally safe and side effects are rare. Pain, bruising or discomfort at the needle placement site may occur. Sometimes apheresis causes a tingling sensation around the mouth or in the fingers. It may cause chills, nausea, heartburn or mild muscle cramps. This can usually be relieved by slowing or temporarily interrupting the apheresis or taking a calcium containing antacid, such as Tums®. Other possible side effects are anxiety, vomiting and lightheadedness. Temporary lowering of the blood pressure may develop. There is the rare possibility of infection, fainting or seizure. Very rarely a nerve problem at the needle placement site may occur. Also, very rarely, a machine malfunction may occur, resulting in the loss of about one unit (one pint) of blood.

There are theoretical risks from re-infusion of the blood after processing by the machine such as infection or an adverse reaction to the blood components. However, these risks must be exceedingly rare if they occur, since they have not been seen in many thousands of volunteers who have undergone this or similar procedures to date.

There may be additional risks of apheresis that are unknown at this time. Any new information that may affect your willingness to participate in this study will be disclosed to you.

Vaccine Risks

General Risks of Vaccines: The possible risks for vaccines in general include fever, chills, rash, aches and pains, nausea, headache, dizziness, and fatigue. We know these side effects can occur with this vaccine too. The side effects don't usually last long. As with all vaccines or drugs, you could have an immediate allergic reaction, including a rash, hives, or difficulty breathing. Allergic reactions can be life threatening. The clinic staff will watch you for at least 30 minutes after the immunization. There may be other side effects, even serious ones that we don't know about yet. It is important that you report any side effects to the clinic staff as soon as they occur.

Risks of the rAd study vaccine: This is one of three studies in which the adenoviral vector vaccine will be given to people who have already received an experimental DNA vaccine. There is also a study in which the study vaccine alone has been given to people. All of the possible risks or side effects are not known. In previous studies, some people have had a flu-like

condition with fever, headache, muscle aches, tired feeling and chills starting about 12-16 hours after the vaccination and lasting a few hours. A few subjects have had nausea. Some subjects have injection site pain or discomfort in the first few days after a vaccination. The flu-like symptoms and injection site pain or discomfort may be treated with an over-the-counter medicine for pain and fever. It is possible that there will be different or worse reactions than have been seen in previous studies.

The following is a list of conditions possibly related to study vaccination:

- One person had a white blood cell count that was moderately below normal shortly after vaccination. This returned to normal without any symptoms of illness.
- One person had diarrhea for a day shortly after vaccination.
- One person had a mild change in a blood test for liver function that was done 25 days after vaccination. When this mild change did not return to normal, more evaluation was done and the person was found to have a "fatty liver." The condition may have existed before enrollment in the study. In this person other factors related to fatty liver may be increased alcohol use and recent weight gain.
- One person with a history of a single seizure about 3 years before enrolling in the study had a seizure 64 days after the study vaccination. The seizure was considered unlikely to be related to the study vaccine because it happened more than 2 months after vaccination in a person with a history of having a seizure.
- Other people have had mild temporary changes in blood or urine tests.

It is not known if these problems were due to the vaccine or due to other causes or due to a combination of the vaccine with other causes.

Animals have tolerated a vaccination schedule with a DNA vaccine given first followed by the adenoviral vector vaccine. The adenoviral vector vaccine alone was tested in rabbits. A DNA vaccine similar to the one you got in VRC 007, followed by the adenoviral vector vaccine was also tested in rabbits. In both schedules some rabbits had a fever the day after adenoviral vaccine injection. Some rabbits ate less food than usual for 1-2 days after adenoviral vaccine injection. In some rabbits there was inflammation in the tissue near the injection site. Three blood tests showed a brief change from normal in some rabbits. These were cholesterol, triglycerides and a muscle enzyme (creatine phosphokinase). These blood tests returned to normal.

<u>Other adenoviral vector risks</u>: Other adenoviral vector vaccines and products have been given to humans in other studies that tested treatment of cancer and inherited conditions. In one study a volunteer died from a reaction to an adenovirus vector after he was given a large dose directly into the main liver blood vessel for the purpose of treating an inherited condition. In the study in which you will be participating much lower doses are used and the injection is into your arm muscle.

During the study, regular blood tests and check-ups will be performed to monitor these possible side effects. Some blood will be stored during the study in case additional safety tests are needed.

Antibody to adenovirus: You may develop antibodies to adenovirus type 5 from the experimental vaccine. It is possible you would not be able to receive (or have a reduced response to) future products that used an adenoviral vector. Currently there are no products approved by the FDA that use an adenoviral vector.

HIV Infection: You will be counseled about HIV exposure during the study. If you have questions, please ask the clinical staff. Taking this test vaccine may mean that you may not be able to take other experimental HIV vaccines later. It is also possible that receiving the experimental HIV vaccine may alter your response to future HIV vaccines and may make them either more or less effective. If you are exposed to HIV through sex or drug use after receiving the study injection, your risk of becoming HIV infected is unknown. Please do not do anything that might expose you to HIV.

Risks from Blood Drawing

Blood drawing may cause pain and bruising and, rarely, infection at the place where the blood is taken. Sometimes drawing blood causes people to feel lightheaded or even faint.

Risks of a "False Positive" HIV Antibody Test

At the time you enroll in the study you may have a negative HIV antibody test or you may have a positive HIV antibody test from your participation in VRC 007. An HIV antibody test (called an ELISA, EIA or Western Blot) is the usual way to test for HIV infection. After injection of an HIV vaccine, you may test positive for HIV antibody from the vaccine. However, it will be possible, by using tests for the presence of HIV virus (called PCR or viral load testing), to show when a positive result on the HIV antibody test is NOT because of an HIV infection. A positive antibody test in a person who is not HIV infected is called a "false positive" test. If you do have a false positive HIV antibody test caused by experimental HIV vaccine, it is unknown how long the test will be positive. Antibodies resulting from a vaccine do not always continue to be present long term. If you have a false positive antibody test at the end of the study you will be offered the chance to be retested once per year for five years so that you can find out if it changes back to negative. You may be subjected to the social risks of having your HIV test appear positive.

Any time you have a positive HIV antibody test in the future you must also have an HIV viral load test. Otherwise you will not know if the positive HIV antibody test is from the study vaccine or from HIV infection.

You will not be able to donate blood while you are participating in the study and for at least one year after the study injection. You may not be able to donate blood ever again if you have a false positive HIV antibody test when you try to donate blood. Please be sure you have a negative HIV antibody test before trying to donate blood.

If you have a false positive antibody response on HIV tests, you may also have difficulties with:

- Health insurance

- Life Insurance
- Medical or dental care
- Travel to other countries or immigration
- Employment
- Education
- Housing
- Military services or other government agencies
- Personal relationships

If you have problems like these, the staff at the VRC clinic will try to help you work through them. If your blood tests look HIV positive because of study vaccinations you will be offered a letter that shows you joined this study and that describes the antibody response caused by the vaccine. Even so, this letter or other help offered by the VRC clinic may not solve a social problem caused by a false positive HIV antibody test.

It is also possible that others may learn that you are taking part in this study and assume that you are at risk of HIV infection because of sexual behavior or drug use. This may result in some people treating you differently.

Risks from Pregnancy

We do not know the possible effects that the vaccine given in this study will have on the fetus or nursing infant. Therefore, women who are able to become pregnant must have a negative pregnancy test before the study injection. Women who are heterosexually active must also agree to practice adequate birth control beginning at least 21 days prior to receiving the first injection until the last study visit, or not be able to have children. Adequate methods of birth control include: condoms, male or female, with or without a spermicide; diaphragm or cervical cap with spermicide; intrauterine device; contraceptive pills or patch, Norplant, or Depo-Provera or other FDA-approved contraceptive; or having a male partner who has previously undergone a vasectomy for which there is documentation. If you are pregnant, breast-feeding or want to become pregnant during the next 6 months, you cannot participate. You must notify the clinic staff immediately upon learning that you have become pregnant during this study. If you become pregnant, you will be asked to continue with study follow-up visits through the end of the study and to report the outcome of the pregnancy.

Other Risks

The safety and toxicity of the study vaccine are unknown. There is a risk of serious side effects. It is possible that new and unexpected side effects may develop in humans that were not observed in animal testing or the other people who have received the study vaccine. You will be told of significant health effects of the study vaccine or serious side effects if any occur in other people who get the study vaccine.

POSSIBLE BENEFITS

This study will be of no direct benefit to you because no one knows if this vaccine helps to prevent HIV. You and others may benefit in the future from the information that will be learned from the study.

COSTS TO YOU FOR YOUR PARTICIPATION

You do not have to pay for the study vaccine, research clinic visits, examinations or laboratory tests that are part of this study. All other medical costs outside this study will be paid by you or your health insurance carrier (if you have insurance).

PAYMENT TO YOU FOR YOUR PARTICIPATION

You will be compensated $100 for the clinic visit that includes the injection, $140 for a clinic visit that includes apheresis and $70 for a clinic visit that does not include an injection. The approximate total compensation is about $450 if an apheresis is not done and $520 if an apheresis is done. The actual amount will be based on the number of study visits you attend, whether the study injection is received and whether an apheresis is done. You will be paid throughout the study after each reimbursable visit.

REASONS FOR REMOVING YOU FROM THE STUDY WITHOUT YOUR CONSENT

You may be asked to leave the study for several different reasons, including:

- You don't keep appointments or follow study procedures.
- The study sponsor or study doctor decides to stop or cancel the study.
- The regulatory agencies, such as the institutional review board or the FDA feels that the study should be stopped.

If you agree to take part in this study, it is important for you to keep all your appointments. However, if you don't want to stay in the study, you can leave at any time. You will not lose any benefits that you would have if you had not joined the study. Once you get the study vaccination on the day of enrollment, you are advised to complete all the safety evaluations that are part of the study.

EARLY WITHDRAWAL FROM THE STUDY

If you decide to withdraw from the study early, please tell a study doctor or study nurse so that you can be advised about suggestions for monitoring your health. If you withdraw from the study early you may not learn whether you are having any side effects from the vaccine because you will not have the follow-up health check-ups included in the study.

ALTERNATIVES

This study is not designed to treat any disease that you have. You may choose not to participate.

OTHER PERTINENT INFORMATION

1. Confidentiality. When results of an NIH research study are reported in medical journals or at scientific meetings, the people who take part are not named and identified. In most cases, the NIH will not release any information about your research involvement without your written permission. However, if you sign a release of information form, for example, for an insurance company, the NIH will give the insurance company information from your medical record. This information might affect (either favorably or unfavorably) the willingness of the insurance company to sell you insurance.

The Federal Privacy Act protects the confidentiality of your NIH medical records. However, you should know that the Act allows release of some information from your medical record without your permission, for example, if it is required by the Food and Drug Administration (FDA), members of Congress, law enforcement officials, or other authorized people.

2. Policy Regarding Research-Related Injuries. The Clinical Center will provide short-term medical care for any injury resulting from your participation in research here. In general, no long-term medical care or financial compensation for research-related injuries will be provided by the National Institutes of Health, the Clinical Center, or the Federal Government. However, you have the right to pursue legal remedy if you believe that your injury justifies such action.

3. Payments. The amount of payment to research volunteers is guided by the National Institutes of Health policies.

4. Problems or Questions. If you have any problems or questions about this study or about any research-related injury, contact the Principal Investigator, Andrew Catanzaro at 301-402-8604. You may also call Barney S. Graham, M.D., Ph.D at 301-594-8468 or Steven Rucker, RN, at 301-451-8715. The 24-hour pager number for the study is 1-800-NIH-BEEP ext 8459. If you have any questions about your rights as a research subject, you may call the Clinical Center Patient Representative at 301-496-2626.

5. Consent Document. Please keep a copy of this document in case you want to read it again.

COMPLETE APPROPRIATE ITEM(S) BELOW:	
**A. Adult Study Participant's Consent**<br>I have read the explanation about this study and have been given the opportunity to discuss it and to ask questions. I hereby consent to take part in this study.<br>Time<br>Signature of Adult Participant/Legal Representative Date	**B. Parent's Permission for Minor Participant.**<br>I have read the explanation about this study and have been given the opportunity to discuss it and to ask questions. I hereby give permission for my child to take part in this study.<br>(Attach NIH 2514-2, Minor's Assent, if applicable.)<br>Signature of Parent(s)/Guardian Date
**THIS CONSENT DOCUMENT HAS BEEN APPROVED FOR USE FROM XXXXXX THROUGH XXXXXX.**	
Time:<br>Signature of Investigator/Person Obtaining Consent Date	Time:<br>Signature of Witness Date

APPENDIX II
CONTACT INFORMATION

**Principal Investigator**:<br>Andrew Catanzaro, M.D. 301-402-8604<br>Vaccine Research Center, NIAID, NIH<br>40 Convent Drive, MSC 3017<br>Bethesda, MD 20892-3017<br>1-800-NIH-BEEP ext 5757<br><br>**Subinvestigators**:<br>Barney S. Graham, M.D., Ph.D. 301-594-8468<br>Joseph Casazza, M.D., Ph.D. 301-594-8627<br>Julie Martin, D.O. 301-594-8559<br>Janie Parrino, M.D. 301-402-8832<br><br>**Study Coordinators**<br>Steven Rucker, RN 301-451-8715<br>VRC 010 Study Coordinator<br>Building 10, 12 West Outpatient Clinic<br>Bethesda, MD 20892<br><br>Pamela Edmonds, RN, BSN, MSA<br>Ingelise Gordon, RN<br>Lasonji Holman, CFNP<br>Sarah A. Hubka, RN, MSN, NP<br>Brenda Larkin, RN, BSN, CCRC<br>Margaret McCluskey, RN, MPH, CCRC<br>Laura Novik, RN, MA, CCRC<br><br>**DAIDS Medical Officer:**<br>Chuen-Yen Lau, M.D. 301-451-2779<br>6700 B Rockledge Dr., Bethesda, MD 20892<br><br>**Protocol Statistician**<br>Martha Nason, Ph.D. 301-451-5134<br>Biostatistical Research Branch, NIAID<br><br>**NIH Apheresis Clinic**<br>Susan Leitman, M.D. 301-496-9703<br>Department of Transfusion Medicine<br>10 Center Drive, Building 10-MSC 1184<br>Bethesda, MD 20892-1184<br><br>**Data Coordinating Center**:<br>Vaccine Research Center, NIAID, NIH and<br>EMMES Corporation, Rockville, MD<br><br>**Site And Data Monitoring:**<br>PPD Development<br>Wilmington, NC	**Scientific and Laboratory Collaborators**:<br>Gary Nabel, M.D., Ph.D.<br>Vaccine Research Center, NIAID, NIH<br>40 Convent Drive, MSC 3017<br>Bethesda, MD 20892<br><br>Laboratory of Immunology<br>VRC/NIAID/NIH:<br><br>Robert Bailer, Ph.D.<br>301-594-8481<br><br>Daniel Douek, M.D, Ph.D.<br>301-594-8484<br><br>Richard Koup, M.D.<br>301-594-8585<br><br>John Mascola, M.D.<br>301-594-8490<br><br>Mario Roederer, Ph.D.<br>301-594-8491<br><br>**Vaccine Manufacturer**:<br>Victoria Haque 240-632-0740<br>GenVec, 65 Watkins Mill Road<br>Gaithersburg, MD 20878<br><br>**Pharmacy Affairs Branch:**<br>Judith Starling, R.Ph., Hope DeCederfelt, R.Ph.<br>Pharmaceutical Development Section<br>Clinical Center, Building 10/1N257<br>Bethesda, MD 20892<br>301-496-4363<br><br>**VRC Production and Regulatory Affairs:**<br>Phillip Gomez III, Ph.D., 301-594-8485<br>Judy Stein, MPH, MBA, 734-763-7753<br>Charla Andrews, Sc.M., 301-594-8488<br><br>**VRC Protocol Section:**<br>Mary E. Enama, M.A., PA-C, 301-594-8501<br>Richard Jones, 301-451-8543<br><br>**DAIDS, Regulatory Compliance Center:**<br>SAE Phone: 1-800-537-9979 or 301-897-1709<br>SAE Fax: 1-800-275-7619 or 301-897-1710<br>SAE e-mail: RCCSafetyOffice@tech-res.com

APPENDIX III
SCHEDULE OF EVALUATIONS

		**VRC 000**	**VRC 010**						
Visit		*01	02	02B	02C	02D	03	04	05
Week of Study		- 4 to 0	Wk 0	W 1	W2	W 4	W 6	W 12	W 24
[1]Day of Study		-28 to 0	Day 0	D 2	D14	D 28	D 42	D 84	D 168
**Clinical**	Tube								
VRC 010 Assessment of Understanding; VRC 010 Study Consent			X						
[2] Physical exam		X	[X]		[X]	[X]	[X]	[X]	[X]
Vital Signs; lymph node assess.		X	X		X	X	X	X	X
Complete medical history or interim medical history		X	X		X	X	X	X	X
Study Injection			X						
Phone evaluation (clinic visit, if needed)				X					
5-day Diary Card			Card x 5d		Return card				
Social Impact Assessment									X
[3] Counseling on HIV; pregnancy		X	X		X	X	X	X	X
Urinalysis		*X	X		X		X	X	X
CBC, differential, platelets	Lavender	*3	3		3		3	3	3
[4] Pregnancy test: urine (or serum)		X	*X						X
Chemistry: creatinine, ALT	SST	*4	4		4		4	4	4
HBsAg, Anti-HCV, RPR	SST	*8							
ELISA/Western Blot	SST	*4	4				4		4
HIV PCR	Lavender	3	3				3		3
T Cell FACS	Lavender	3	3						3
**Research**									
[5]Adenovirus Serology	SST		[5]use SS			[5]use SS			[5]use SS
Neutralizing Antibody Titers and vaccine antigen-specific ELISA	SST		16			16			16
Cell Immunology Assays (ICS and ELISPOT); PBMC storage and Plasma for Storage	EDTA or ACD		80		60	[6]Apheresis & 10 mL or 80 mL	60	80	80
Serum Storage	SST		8		8	16	8	24	16
**Daily Volume (mL)**		**25**	**121**		**75**	**112**	**82**	**111**	**129**
**Max. Cumulative Volume (mL)**		**25**	**146**		**221**	**333**	**415**	**526**	**655**

* Screening evaluations must be no more than 28 days prior to Day 0 to be used for eligibility. Results of pregnancy test on Day 0 must be used for eligibility. If clinical assessment on Day 0 suggests significant changes may have occurred since the screening visit, then a physical examination, hematology tests, blood chemistries and urinalysis done on Day 0 must be used for eligibility.

[1] Day 0=day of enrollment and injection; Day 0 evaluations prior to first injection are the baseline for assessing adverse events subsequently; Visit 02A is the 30-45 minute post-injection evaluation. Schedule Visit 02B for 1 or 2 days after injection, visit 02C for 14±3 day after the injection, visit 02D for 28±3 days after the injection. Schedule visit 03 with a ±3 days window. Schedule visits 04 and 05 with a ±14 days window from the target day shown.

[2] The Screening visit includes a physical exam; during other visits a physical exam is done only if indicated by interim history or laboratory test results (shown as [X] in the table).

[3] Counseling at Screening and on Day 0 and offered on each subsequent visit

[4] May be urine or serum test; serum test can be done from the same tube drawn for blood chemistries.

[5] Use Serum Storage (SS) at Week 4 and Week 24 to repeat the Adenovirus Serology testing; a separate SST tube is collected for this purpose on Day 0.

[6] At 02D, PBMC will be collected by apheresis whenever possible along with plasma from one 10 mL blood sample; otherwise collect 80 mL blood by phlebotomy to obtain the PBMC and plasma.

APPENDIX IV
TABLE FOR GRADING SEVERITY OF ADVERSE EVENTS

The Division of AIDS Table for Grading the Severity of Adult and Pediatric Adverse Events, Version 1.0, Dec 2004

The table for Grading Severity of Adverse Events in this protocol is found on the Division of AIDS Regulatory Compliance Center (RCC) website:

http://rcc.tech-res-intl.com/eae.htm

A complete copy of this 20 page table will be provided to the IRB for reference with initial review of the protocol. The table cannot be changed except by the IND Sponsor, DAIDS.

The full text of the table will also be included in the Protocol Manual for reference by the study clinicians who are assessing adverse events.
